# Pathway selection in the self-assembly of Rh_4_L_4_ coordination squares under kinetic control

**DOI:** 10.1038/s42004-023-01053-7

**Published:** 2023-11-15

**Authors:** Atsushi Okazawa, Naoki Sanada, Satoshi Takahashi, Hirofumi Sato, Shuichi Hiraoka

**Affiliations:** 1 Department of Electrical Engineering and Bioscience, Waseda University, Tokyo, 169-8555 Japan; 2 Department of Basic Science, Graduate School of Arts and Sciences, The University of Tokyo, Tokyo, 153-8902 Japan; 3 Department of Molecular Engineering, Kyoto University, Kyoto, 615-8510 Japan; 4 Fukui Institute for Fundamental Chemistry, Kyoto University, Kyoto, 606-8103 Japan

**Keywords:** Self-assembly, Computational chemistry, Coordination chemistry

## Abstract

Pathway selection principles in reversible reaction networks such as molecular self-assembly have not been established yet, because achieving kinetic control in reversible reaction networks is more complicated than in irreversible ones. In this study, we discovered that coordination squares consisting of *cis*-protected dinuclear rhodium(II) corner complexes and linear ditopic ligands are assembled under kinetic control, perfectly preventing the corresponding triangles, by modulating their energy landscapes with a weak monotopic carboxylate ligand (2,6-dichlorobenzoate: dcb^–^) as the leaving ligand. Experimental and numerical approaches revealed the self-assembly pathway where the cyclization step to form the triangular complex is blocked by dcb^–^. It was also found that one of the molecular squares assembled into a dimeric structure owing to the solvophobic effect, which was characterized by nuclear magnetic resonance spectroscopy and single-crystal X-ray analysis.

## Abstract (web summary)

Achieving kinetic control over reversible self-assembly processes remains challenging. Here, the authors assemble Rh_4_L_4_ coordination squares consisting of *cis*-protected Rh(II) corner sites and linear ditopic ligands under kinetic control, preventing the formation of triangles by modulating the energy landscape with use of a leaving ligand.

## Introduction

Pathway-dependent molecular self-assembly has often been seen in the realm of nature, especially in the multicomponent self-assembly of viruses^1^ and proteasomes^2^. One of the reasons why such a sequential assembly process is adopted is because the formation of undesired assemblies whose structures are far from the desired one is efficiently prevented in the assembly process. If there exist some other assemblies with similar thermodynamic stabilities, selective assembly of the desired one under thermodynamic control is impossible. In such cases, pathway selection under kinetic control is inevitable, but the general principles of pathway selection in complicated reversible reaction networks have yet to be fully understood^3^.

Molecular square is one of the most representative molecular architectures in coordination self-assembly^4–24^. Thanks to the L–M–L bond angle of 90° around transition metal ions with octahedral and square planer coordination spheres, upon complexation with linear ditopic ligands, square complexes are expected to be thermodynamically most stable over other polygonal structures. However, in many cases triangular complexes were coproduced in solution even using rigid linear ditopic ligands as the side of the polygons, although triangles are geometrically largely different from squares^25–45^. Therefore, the selective formation of molecular squares and the understanding of their origin remain challenging in supramolecular chemistry^46^.

Under the condition where the energy barriers between square and triangular compounds are relatively high, there is a chance to produce the square in a higher yield than that obtained at equilibrium, kinetically preventing the triangle. Such a kinetic approach is quite limited in artificial molecular self-assembly, except for unexpected observations of kinetic traps^47–55^. Our understanding of the energy landscapes of molecular self-assembly is not as thorough as we can rationally design them, and to the best of our knowledge, there is no report on the selective formation of a triangular or square complex under kinetic control.

**Fig. 1:**
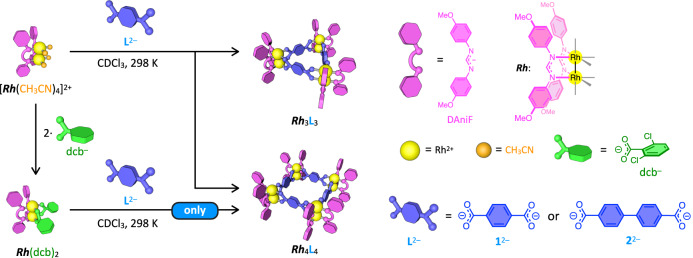
Self-assembly of the *Rh*_4_L_4_ squares in this study. Schematic representation of kinetically controlled self-assembly of the ***Rh***_4_L_4_ squares from *cis*-protected dinuclear Rh(II) complex (***Rh***^2+^: [Rh_2_(DAniF)_2_]^2+^) and linear dicarboxylate ligand (L^2–^: **1**^2–^ or **2**^2–^) in solution. When the metal source is [***Rh***(CH_3_CN)_4_]^2+^, where CH_3_CN is the leaving ligand, ***Rh***_3_L_3_ triangle and ***Rh***_4_L_4_ square complexes were produced in a 1:2 ratio. In contrast, when the self-assembly was carried out using ***Rh***(dcb)_2_ as the metal source (dcb^–^: 2,6-dichlorobenzoate), dcb^–^ modulates the energy landscape of the self-assembly so that the formation of the ***Rh***_3_L_3_ triangle is prevented, producing the ***Rh***_4_L_4_ square only. The axial ligands on the dinuclear Rh(II) centers are omitted for clarity.

Here, we report the self-assembly of the ***Rh***_4_L_4_ squares in solution under kinetic control, perfectly preventing the formation of the ***Rh***_3_L_3_ triangle using a designed monotopic carboxylate as the leaving ligand (dcb^–^ in Fig. 1). Experimental and numerical analyses of the self-assembly mechanism indicate that the free energy landscape of the self-assembly was properly modulated so that the pathways to the triangular complex were blocked. Furthermore, the monocarboxylate facilitates ligand exchanges, resulting in the conversion of the kinetically trapped triangles into the squares under mild condition. It was also found that one of the ***Rh***_4_L_4_ squares aggregates to form a structurally well-defined dimer in solution by the solvophobic effect, owing to the electronically neutral nature of the Rh(II) complexes.

## Results and discussion

### Self-assembly of Rh(II)-triangle and square with CH_3_CN as the leaving ligand

Cotton and his co-workers and others reported that molecular squares composed of *cis*-protected dinuclear Rh(II) complexes (***Rh***^2+^: [Rh_2_(DAniF)_2_]^2+^^56^ and [Rh_2_(O_2_C-R-CO_2_)]^2+^^57^) and linear dicarboxylates (L^2–^) were selectively obtained in crystalline states^56,57^. As Rh(II)–carboxylate coordination bonds in dinuclear Rh(II) complexes are relatively strong, the reversibility of the coordination bond is low^58^, which would allow the self-assembly of the ***Rh***_4_L_4_ squares to proceed under kinetic control. Thus, we were interested in why the ***Rh***_4_L_4_ squares were selectively produced in the solid state in the previous research.

**Fig. 2:**
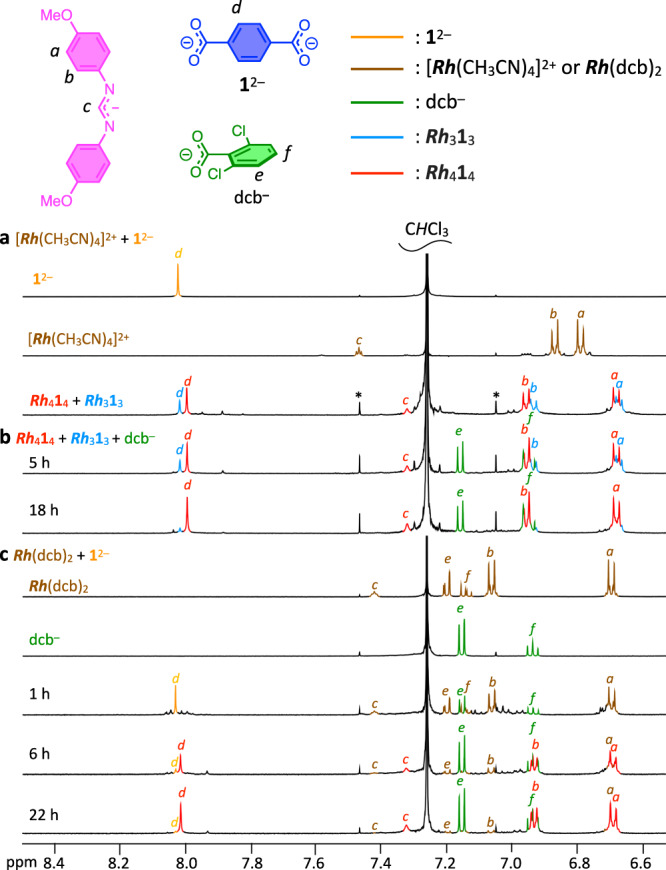
^1^H NMR spectra of the self-assembly of the *Rh*_4_1_4_ square. ^1^H NMR spectra (500 MHz, CDCl_3_, 298 K, aromatic region) of the self-assembly of the ***Rh***_4_L_4_ squares under various conditions ([**1**^2–^] = [***Rh***^2+^] = 1 mM). **a** Self-assembly from [***Rh***(CH_3_CN)_4_](BF_4_)_2_ and **1**^2–^ in CDCl_3_ at 298 K, giving the ***Rh***_3_**1**_3_ triangle and the ***Rh***_4_**1**_4_ square in 23% and 39% yields, respectively. Asterisks indicate the carbon satellite of CHCl_3_. **b** Addition of *n*-Bu_4_N·dcb in a mixture of the ***Rh***_3_**1**_3_ triangle and the ***Rh***_4_**1**_4_ square obtained from the self-assembly of [***Rh***(CH_3_CN)_4_](BF_4_)_2_ and **1**^2–^ in CDCl_3_ after convergence. The ***Rh***_3_**1**_3_ triangle was converted into the ***Rh***_4_**1**_4_ square at 298 K assisted by dcb^–^, although heating at 100 °C for 2 days is necessary without dcb^–^ (Supplementary Fig. 14). **c** Self-assembly of the ***Rh***_4_**1**_4_ square from ***Rh***(dcb)_2_ and **1**^2–^ in CDCl_3_ at 298 K to produce the ***Rh***_4_**1**_4_ square in a 65% yield without formation of the ***Rh***_3_**1**_3_ triangle during self-assembly. The yields were determined based on the internal standard.

We first performed the self-assembly from [***Rh***(CH_3_CN)_4_](BF_4_)_2_ and **1**^2–^ in CH_3_CN ([**1**^2–^] = 10 mM) according to the literature^56^. Dark red solid precipitated in 1 day. Its ^1^H NMR spectrum in CDCl_3_ showed two prominent singlet signals of H^*d*^ (aromatic protons of **1**^2–^ as shown in Fig. 2) in a 1:3 integral ratio, suggesting the formation of two highly symmetric cyclic structures (Supplementary Fig. 1). Crystallization of this mixture in CHCl_3_ gave single crystals of ***Rh***_4_**1**_4_ as was reported in the literature^56^. The major ^1^H NMR signals are the same as those of ***Rh***_4_**1**_4_ (Supplementary Fig. 2), so the minor signals are expected to correspond to the ***Rh***_3_**1**_3_ triangle, which is supported by ^1^H DOSY spectroscopy (Supplementary Fig. 3), ESI-TOF mass spectrometry (Supplementary Fig. 4a), and other experiments (details are shown in Supplementary Fig. 2). Therefore, the ***Rh***_4_**1**_4_ square and the ***Rh***_3_**1**_3_ triangle were produced in CH_3_CN.

To investigate the solution process, self-assembly was performed in CDCl_3_ at 298 K ([**1**^2–^] = 1.0 mM), where precipitation did not occur during the self-assembly, resulting in a mixture of ***Rh***_3_**1**_3_ and ***Rh***_4_**1**_4_ in a 1:2 ratio (Fig. 2a). Similarly, self-assembly of [***Rh***(CH_3_CN)_4_](BF_4_)_2_ and another ditopic ligand (**2**^2–^) in CDCl_3_ gave the triangular and square complexes in a 1:2 ratio (Supplementary Figs. 2, 4c, 5, and 6). Therefore, the self-assembly of [***Rh***(CH_3_CN)_4_]^2+^ and the linear ditopic ligands (L^2–^) at room temperature gave a mixture of a molecular square (major) and a triangle (minor) in solution, and the ***Rh***_4_**1**_4_ square was selectively crystallized from the mixture.

### Kinetic self-assembly of Rh(II)-squares

We then investigated the kinetic effect of the leaving ligand on ***Rh***-based self-assembly. 2,6-Dichlorobenzoate (dcb^–^) was chosen as the leaving ligand (Fig. 1). Because the p*K*_a_ value of Hdcb in H_2_O (1.82)^59,60^ is lower than that of benzoic acid (4.21), the coordination ability of dcb^–^ is lower than those of the ditopic ligands (L^2–^), suggesting that dcb^–^ can act as a leaving ligand. To test the potential of dcb^–^ as a leaving ligand, the ligand exchange of dcb^–^ in ***Rh***(dcb)_2_ with *p*-toluate (tol^–^) in CDCl_3_ was monitored by ^1^H NMR spectroscopy (Supplementary Fig. 7a). The ligand exchange proceeded at 298 K to produce ***Rh***(tol)_2_ in 82% yield (Supplementary Fig. 7b), indicating that dcb^–^ can be used as a leaving ligand in ***Rh***-based self-assembly at room temperature.

The self-assembly of ***Rh***(dcb)_2_ and L^2–^ (**1**^2–^ and **2**^2–^) in CDCl_3_ at 298 K was monitored by ^1^H NMR spectroscopy (Fig. 2c and Supplementary Fig. 8). Surprisingly, the ^1^H NMR signals of the ***Rh***_3_L_3_ triangle did not appear at all during the self-assembly for **1**^2–^ and **2**^2–^. These results indicate that the energy landscape of the self-assembly was dramatically altered by dcb^–^, kinetically producing only the ***Rh***_4_L_4_ squares.

### Self-assembly mechanism of Rh(II)-squares

We were interested in why the ***Rh***_4_L_4_ squares were selectively produced with dcb^–^ as the leaving ligand under kinetic control, so the self-assembly processes were investigated by QASAP (quantitative analysis of self-assembly process)^61–63^. Basically, it is difficult to obtain information about intermediates in molecular self-assembly, because most of intermediates are unobservable. Indeed, only small minor signals for intermediates were observed by ^1^H NMR spectroscopy during the self-assembly of the ***Rh***_4_L_4_ squares (Fig. 2c and Supplementary Fig. 8). In such cases, QASAP provides valuable insight into the self-assembly pathway from time-development of the average composition of all intermediates even when the intermediates cannot be observed. We have applied QASAP to Pd(II)- and Pt(II)-based coordination self-assemblies so far to determine the major self-assembly pathway(s) and the rate-determining step. QASAP of the ***Rh***_4_L_4_ squares is the first example of QASAP for metal-organic assemblies with transition metals other than Pd(II) and Pt(II) ions.

**Fig. 3:**
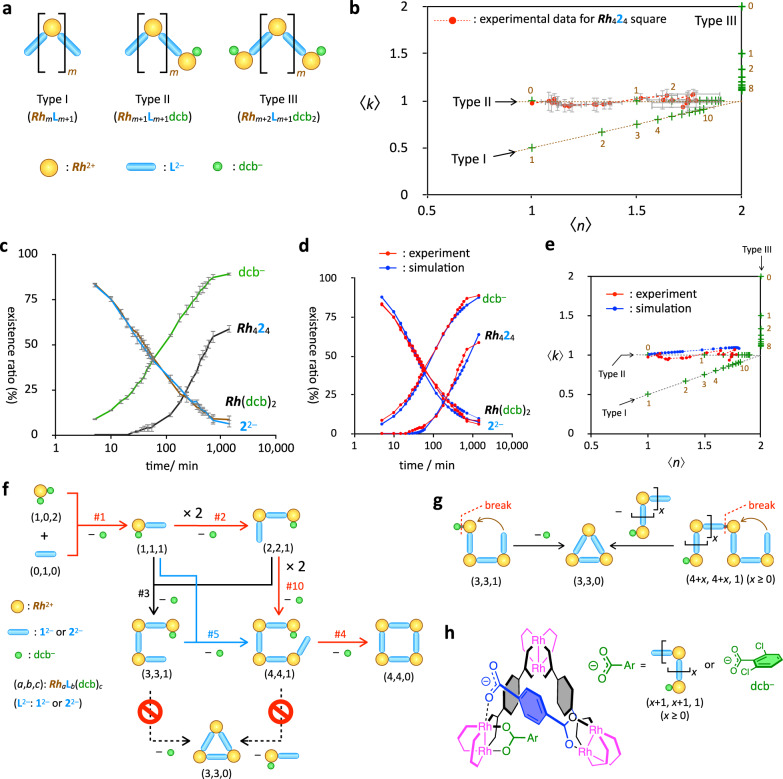
Self-assembly mechanism of the *Rh*_4_2_4_ square under kinetic control. **a** Three types of possible chain intermediates in the self-assembly of M_4_L_4_ square. Type I: M_*m*_L_*m*+1_, Type II: M_*m*+1_L_*m*+1_(dcb), Type III: M_*m*+2_L_*m*+1_(dcb)_2_. **b** Plots of the existence ratios of the substrates and products in the self-assembly of the ***Rh***_4_**2**_4_ square from ***Rh***(dcb)_2_ and **2**^2–^ in CDCl_3_ at 298 K. [***Rh***]_0_ = [**2**^2–^]_0_ = 0.86 mM. **c** Plots of the (〈*n*〉, 〈*k*〉) values in the *n-k* map of the ***Rh***_4_**2**_4_ square (red filled circles). Green crosshairs indicate the (*n*, *k*) values of the chain intermediates. The three types of chain intermediates, Types I, II, and III, are plotted on each straight line. The brown number indicates *m* in each type of oligomer in **a**. The definitions of 〈*n*〉 and 〈*k*〉 values are shown in the main text (Eqs. 1 and 2). The data in (**b**) and (**c**) are shown as the average of the three runs of QASAP with standard errors. **d** Comparison of the existence ratio of the substrates and the products between QASAP (red) and NASAP (blue). **e** Comparison of the *n*-*k* plot between QASAP (red) and NASAP (blue). Both (**d**) and (**e**) indicate that the numerical simulation results reproduce the experimental counterparts well. **f** Dominant self-assembly pathways of the ***Rh***_4_**2**_4_ square. Red arrows indicate the major self-assembly pathway. (*a,b,c*) indicates ***Rh***_*a*_**2**_*b*_(dcb)_*c*_. The numbers above the reaction arrows indicate the elementary reactions with high net reaction frequency listed in Supplementary Table 4. Longer Type II oligomers than (4,4,1), such as (5,5,1) and (6,6,1), are produced during the self-assembly, although they are not involved in the major self-assembly pathway (Supplementary Fig. 13). **g** Two possible pathways to produce triangle (3,3,0) from Type II intermediate (3,3,1) (left) and from Type II oligomers with more than three ***Rh***^2+^ units (4+*x*, 4+*x*, 1) (*x* ≥ 0) (right). The green sphere indicates the leaving ligand(s) (dcb^–^ or two molecules of CH_3_CN). **h** A plausible key structure in the triangular formation through associative ligand exchange process. Steric repulsion caused by ArCO_2_^–^ (in green) in the cyclic intermediate and transition state of the triangular formation process would prevent the cyclization.

The substrates (***Rh***(dcb)_2_ and L^2–^) and the products (***Rh***_4_L_4_ and dcb^–^) were quantified by ^1^H NMR spectroscopy (Fig. 3c), and the change in the average composition of all intermediates with time was plotted in a 2D map (*n-k* map) (Fig. 3b). The species regarding the self-assembly are expressed by ***Rh***_*a*_L_*b*_(dcb)_*c*_ (*a*–*c* are 0 or positive integer), which is indicated as (*a*,*b*,*c*) for simplicity. The (*n*, *k*) values of ***Rh***_*a*_L_*b*_(dcb)_*c*_ are defined as *n* = (2*a* – *c*)/*b* and *k* = *a*/*b*^61–63^. The *n* value indicates the average number of ***Rh***^2+^ bound to a single ditopic ligand, L^2– ^. The *k* value indicates the ratio of ***Rh***^2+^ against L^2–^. The experimentally obtained (*n*, *k*) values, which is indicated as (〈*n*〉, 〈*k*〉), are calculated for the average composition of all intermediates (***Rh***_〈*a*〉_L_〈*b*〉_(dcb)_〈*c*〉_), whose time-development was used for the discussion on the self-assembly process (Fig. 3b).

There are three types of chain intermediates classified according to the difference in the terminals (Types I–III) (Fig. 3a)^64,65^. The (*n*, *k*) values of the three types of oligomers are distinctly plotted on different straight lines in the *n*-*k* map (Fig. 3b). With an increase in the degree of oligomerization (*m* in Fig. 3a), the (*n*, *k*) values become close to (2, 1), which is the (*n*, *k*) value of the cyclic structures (triangle and square). Thus, the (*n*, *k*) plot enables us to discuss which type of oligomers was mainly produced and how large the chain-like oligomers grew during self-assembly.

With regard to QASAP for the ***Rh***_4_**1**_4_ square, the 〈*n*〉 value was smaller than 1 at an early stage (Supplementary Fig. 9 and Supplementary Table 1), which suggests that ***Rh***^2+^ units in the intermediates have more than two carboxylate ligands (**1**^2–^ and/or dcb^–^), so further analysis could not be performed (for detailed discussion, see Supplementary Fig. 9). Such a strange behavior was not found for the self-assembly of the ***Rh***_4_**2**_4_ square (Fig. 3b and Supplementary Fig. 10 and Supplementary Table 2). The 〈*n*〉 value increased with an almost constant 〈*k*〉 value of 1, indicating the formation of Type II oligomers as main intermediates, whose *m* value finally reached 3 or 4, corresponding to 4 or 5 ***Rh***^2+^ units in the oligomers.

### Numerical analysis of self-assembly pathway

The major self-assembly pathway was determined by numerical analysis (NASAP: numerical analysis of self-assembly process)^66^. In NASAP, the QASAP data were numerically analyzed based on the reaction network model where possible intermediates and their elementary reactions were considered. We prepared a reaction network model consisting of 34 species with a maximum number of ***Rh***^2+^ of 10 and 1694 elementary reactions between them (Supplementary Fig. 11). The elementary reactions in the network were classified into 10 groups according to the reaction type (Supplementary Fig. 12). Then, the forward and backward rate constants for the elementary reactions were used as variable parameters to fit the QASAP data. No good dataset was found unless the rate constants of the triangular formations (*k*_8_ and *k*_9_) were set to almost zero, which is consistent with the experimental result that the ^1^H NMR signals of ***Rh***_3_**2**_3_ did not appear during the self-assembly (Supplementary Fig. 8). A set of rate constants that reproduced the experimental results well (Fig. 3d, e) was found (Supplementary Table 3).

With the dataset of the rate constants thus obtained, the self-assembly of ***Rh***_4_**2**_4_ was numerically simulated in the reaction network, which enabled us to determine the major self-assembly pathway. The top 10 elementary reactions with high net frequencies are listed in Supplementary Table 4. Net frequency represents the difference between the occurrence in the forward direction and that in the backward direction for an elementary reaction; thus, the net frequency indicates the actual degree of progress of the elementary reaction^67^. Connecting the elementary reactions with the highest net reaction frequency from ***Rh***_4_**2**_4_ to the substrates resulted in the dominant self-assembly pathways (Fig. 3f). As expected from the *n-k* analysis, ***Rh***_4_**2**_4_ was assembled, producing Type II species as the major intermediates, and the largest intermediate in the major pathway is ***Rh***_4_**2**_4_(dcb)_1_, which is indicated as (4,4,1) (Fig. 3f).

The change in the existence ratios of the intermediates in the major self-assembly pathway shows that the existence of (4,4,1) is much smaller than that of the other intermediates (Supplementary Fig. 13), indicating that the cyclization of (4,4,1) occurs quickly and that the formation of (4,4,1) from (2,2,1) and from (3,3,1) is the rate-determining steps (Fig. 3f)^68^.

### Reason for the selective formation of Rh(II)-squares under kinetic control

Next, we discuss why the pathways to the ***Rh***_3_L_3_ triangles were blocked with dcb^–^ as the leaving ligand. There are two types of reactions that produce ***Rh***_3_L_3_ by the cyclization of Type II intermediates: (i) the cyclization of (3,3,1) to form (3,3,0) by breaking the coordination bond between Rh(II) and the leaving ligand (CH_3_CN or dcb^–^) (Fig. 3g, left) and (ii) the cyclization of oligomers longer than (3,3,1), (4+*x*, 4+*x*, 1) (*x* ≥ 0), by breaking a Rh–L coordination bond (Fig. 3g, right). Note that the latter reaction is not affected by the leaving ligand. Therefore, the formation of ***Rh***_3_L_3_ triangles from (4+*x*, 4+*x*, 1) (*x* ≥ 0) should be prevented either using ***Rh***(dcb)_2_ or [***Rh***(CH_3_CN)_4_]^2+^. In addition, when dcb^–^ is the leaving ligand, the cyclization of (3,3,1) to form (3,3,0) should be prevented. In other words, the cyclization of ***Rh***_3_L_3_(CH_3_CN)_2_ occurs, while that of ***Rh***_3_L_3_(dcb) is kinetically blocked.

Considering the associative ligand exchange mechanism of Rh(II)–carboxylate bonds^69,70^, triangular formation should take place by the attack of the terminal non-coordinated carboxylate group of L to the axial site of a Rh(II) center in Type II intermediates (Fig. 3h). The instability of a resultant cyclic intermediate or a transition state with dcb^–^ would be the reason for the suppression of the cyclization of ***Rh***_3_L_3_(dcb).

### Conversion of Rh(II)-triangles into Rh(II)-squares assisted by dcb^–^

The conversion between the triangular and square complexes was very slow due to the inertness of Rh–carboxylate bonds. Heating a 1:2 mixture of ***Rh***_3_**1**_3_ and ***Rh***_4_**1**_4_ in CDCl_3_ at 60 °C did not show any change of their ratio (Supplementary Fig. 14a). The conversion of ***Rh***_3_**1**_3_ into ***Rh***_4_**1**_4_ was realized by heating the mixture in dimethylacetamide (DMA) at 100 °C for 2 days (Supplementary Fig. 14b).

Considering the associative ligand exchange mechanism of Rh(II)-carboxylate bonds, the ligand exchange is expected to be facilitated by monotopic carboxylate with weak coordination ability such as dcb^–^. Thus, *n*-Bu_4_N·dcb was added to a mixture of ***Rh***_3_**1**_3_ and ***Rh***_4_**1**_4_ in CDCl_3_, and the reaction at 298 K was monitored by ^1^H NMR spectroscopy (Fig. 2b and Supplementary Table 5). The signals assigned to ***Rh***_3_**1**_3_ slowly decreased with time and almost disappeared after 18 h. The conversion of ***Rh***_3_**1**_3_ into ***Rh***_4_**1**_4_ was also confirmed by ESI-TOF mass spectrometry (Supplementary Fig. 4b). Likewise, the conversion of ***Rh***_3_**2**_3_ into ***Rh***_4_**2**_4_ also occurred at 298 K in 13 h with dcb^–^ (Supplementary Figs. 4d and 15 and Supplementary Table 6). These results indicate that dcb^–^ greatly decreased the energy barriers of the conversion of ***Rh***_3_L_3_ triangles to ***Rh***_4_L_4_ squares as a catalyst. However, the time scale of the conversion of the triangle into the square using the catalyst is much slower than that of the assembly of the square from the substrates (***Rh***_2_(dcb)_2_ and the ditopic ligand). The idea that the selective self-assembly of the ***Rh***_4_L_4_ squares took place by the conversion of the ***Rh***_3_L_3_ triangles into the ***Rh***_4_L_4_ squares by the catalytic effect of dcb^–^ during the self-assembly is ruled out by the fact that the signals of the ***Rh***_3_L_3_ triangles were not observed during the self-assembly (Fig. 2c and Supplementary Fig. 8).

### Supramolecular dimerization of Rh(II)-square by solvophobic effect

**Fig. 4:**
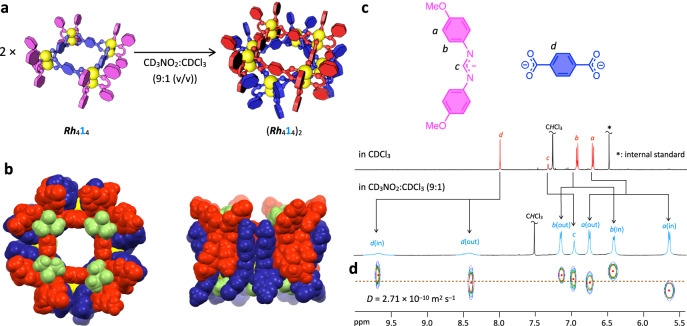
Dimerization of the *Rh*_4_1_4_ square in solution. **a** Supramolecular dimer formation of the ***Rh***_4_**1**_4_ square by the solvophobic effect. **b** Crystal structure of [***Rh***_4_**1**_4_(dmso-*S*)_4_]_2_. Two ***Rh***_4_**1**_4_ squares engaged each other are shown in red and blue. DMSO molecules axially coordinating to the Rh(II) centers are colored in green. **c** ^1^H NMR spectra (500 MHz, 298 K, aromatic region) of (***Rh***_4_**1**_4_)_2_ in CD_3_NO_2_/CDCl_3_ (9:1 (v/v)) and ***Rh***_4_**1**_4_ in CDCl_3_. **d** ^1^H DOSY spectrum of (***Rh***_4_**1**_4_)_2_ in CD_3_NO_2_/CDCl_3_ (9:1 (v/v)).

The dinuclear Rh(II)-based assemblies are neutral molecules, which is largely different from other positively or negatively charged coordination assemblies with counter ions. One of the advantages of neutral coordination assemblies is that they can be assembled into higher order structure without electrostatic repulsion between themselves, leading to a closely packed structure in the crystalline state and in solution. A new set of signals appeared when ***Rh***_4_**1**_4_ was dissolved in CD_3_NO_2_/CDCl_3_ (9:1 (v/v)) (Fig. 4c). A similar spectral change was also observed in acetone-*d*_6_ and CD_3_CN with 10 volume% of CDCl_3_ (Supplementary Fig. 16). A significant up-field shift of aromatic protons (H^*a*^ and H^*b*^) suggests the aggregation of the square (Fig. 4a). All signals except for H^*c*^ were observed as two sets with a 1:1 integral ratio, which is consistent with the crystal structure of the [***Rh***_4_**1**_4_(dmso-*S*)_4_]_2_ dimer (Fig. 4b) obtained from acetone/DMSO.

The (***Rh***_4_**1**_4_)_2_ dimer in CD_3_NO_2_/CDCl_3_ (9:1 (v/v)) was characterized by ^1^H DOSY, (H, H)-COSY, and (H, H)-NOESY spectroscopy (Fig. 4d and Supplementary Figs. 17 and 18). A cross peak was found between H^*d*(out)^ and H^*d*(in)^ protons in the NOESY spectrum (Supplementary Fig. 18), indicating the chemical exchange of the two proton signals caused by the rotation of the 1,4-phenylene ring in **1**^2–^, which would be the reason for the broadening of the two H^*d*^ signals (Fig. 4c). The equilibrium of the monomer (***Rh***_4_**1**_4_) and the dimer ((***Rh***_4_**1**_4_)_2_) shifted toward the dimer with an increase in the composition ratio of CD_3_NO_2_/CDCl_3_ (Supplementary Fig. 19), indicating the dimerization of the ***Rh***_4_**1**_4_ square due to the solvophobic effect. The ^1^H NMR signals of the monomer did not appear even at a low concentration of [***Rh***_4_**1**_4_]_0_ = 4.6 μM (Supplementary Fig. 20). Further dilution caused the NMR spectrum to significantly broaden with a low S/N ratio. According to these results, the dimerization constant for (***Rh***_4_**1**_4_)_2_ is estimated to be higher than 10^7 ^M^–1^ under the assumption that 10% monomer exists (Supplementary Fig. 20).

## Conclusions

In conclusion, the self-assembly of the ***Rh***_4_L_4_ squares was kinetically controlled to produce ***Rh***_4_L_4_ squares selectively assisted by monotopic carboxylate ligand (dcb^–^), which prevents the cyclization of the ***Rh***_3_L_3_(dcb) chain intermediate. This is the first example of the selective formation of coordination squares under kinetic control in solution. The metastable yet kinetically highly stabilized ***Rh***_3_L_3_ triangles can be transformed into the ***Rh***_4_L_4_ squares with dcb^–^ under very mild conditions. These results indicate that the energy landscape of the dinuclear Rh(II)-based coordination self-assembly can be tuned by the leaving ligand. This basic knowledge can be applied to a wide range of Rh(II)-based metal-organic polyhedra (MOPs)^71–73^, whose self-assembly is limited compared with MOPs composed of other transition metal ions owing to the relatively inert equatorial Rh(II)–carboxylate bonds. Because Rh(II)-based MOPs possess high thermal and chemical stabilities and catalytic activity, they are expected as a platform for new materials, so exploring the novel structures of Rh(II)-based MOPs and their efficient self-assembly by modulating the energy landscapes will make a great progress in this field.

## Methods

### General information

^1^H NMR spectra were recorded using a Bruker AV-500 (500 MHz) spectrometer. All ^1^H NMR spectra were referenced using a residual solvent peak, CDCl_3_ (δ 7.26), acetone-*d*_6_ (δ 2.05), CD_3_NO_2_ (δ 4.33). Electrospray ionization time-of-flight (ESI-TOF) mass spectra were obtained using a Waters Xevo G2-S Tof mass spectrometer.

### Materials

Unless otherwise noted, all solvents and reagents were obtained from commercial suppliers (TCI Co., Ltd., WAKO Pure Chemical Industries Ltd., KANTO Chemical Co., Inc., and Sigma-Aldrich Co.) and were used as received. Deuterated solvents were used after dehydration with Molecular Sieves 4 Å. [***Rh***(CH_3_CN)_4_](BF_4_)_2_ (***Rh***: Rh_2_(DAniF)_2_, DAniF: *N*,*N*′-bis(4-methoxyphenyl)formamidinate)^74^ was prepared according to the literature. The ^1^H and ^13^C{^1^H} NMR spectra of all substrates are available in Supplementary Data 1. ***Rh***(dcb)_2_ was prepared by mixing [***Rh***(CH_3_CN)_4_](BF_4_)_2_ and *n*-Bu_4_N·dcb in a 1:2 ration in NMR tube at 298 K and were used for the self-assembly after the quantification based on the internal standard ([2.2]paracyclophane). See more detail in section “Monitoring of the self-assembly of the ***Rh***_4_L_4_ squares”.

### QASAP of the *Rh*_4_L_4_ squares

#### Monitoring of the self-assembly of the Rh_4_L_4_ squares

A 2.4 mM solution of [2.2]paracyclophane in CHCl_3_ (125 μL), which was used as an internal standard, was added to two NMR tubes (tubes **I** and **II**) and the solvent was removed in vacuo. A CDCl_3_ solution of ***Rh***(dcb)_2_ was prepared as solution **A** (10 mM). Solution **A** (50 μL) and CDCl_3_ (400 μL) were added to tube **I**. The exact concentration of ***Rh***(dcb)_2_ in solution **A** was determined through the comparison of the signal intensity with [2.2]paracyclophane by ^1^H NMR. A solution of ditopic ligand (*n*-Bu_4_N)_2_·L (L = **1**^2–^ or **2**^2–^; 2.5 mM) in CDCl_3_ (200 μL) and CDCl_3_ (250 μL) were added to tube **II** and the exact amount of L^2–^ in tube **II** was determined through the comparison of the signal intensity with [2.2]paracyclophane by ^1^H NMR. Then, 1.0 eq. (against the amount of ligand L^2–^ in tube **II**) of solution **A** (*ca*. 50 μL; the exact amount was determined based on the exact concentrations of solution **A** and of L^2–^ in tube **II**) was added to tube **II**. The self-assembly of the ***Rh***_4_L_4_ square was monitored at 298 K by ^1^H NMR spectroscopy. The selected ^1^H NMR spectra measure during the self-assembly are shown in Fig. 2c and Supplementary Fig. 8 ([**1**^2–^]_0_ = 1.0 mM and [**2**^2–^]_0_ = 0.86 mM). The amounts and the existence ratios of L^2–^, ***Rh***(dcb)_2_, the ***Rh***_4_L_4_ square, and dcb^–^ were quantified by the integral value of each ^1^H NMR signal against the signal of the internal standard ([2.2]paracyclophane) according to the determination methodology described in the section “Determination of the existence ratios of each species”. The data, the average values of the existence ratios, and the (〈*n*〉, 〈*k*〉) values with standard errors are listed in Supplementary Tables 1 and 2 for the self-assembly of the ***Rh***_4_**1**_4_ and ***Rh***_4_**2**_4_ squares, respectively.

#### Determination of the existence ratio of each species

The relative integral value of each ^1^H NMR signal against the internal standard [2.2]paracyclophane is used as the integral value in this description. We define the integral values of the signal for the substrates and the products at each time *t* as follows:

#### QASAP for the Rh_4_1_4_ square

*I*_L_(*t*): 1/4 of the integral value of the *d* proton in free ligand **1**^2–^

*I*_M_(*t*): 1/2 of the integral value of the *e* proton of dcb^–^ in ***Rh***(dcb)_2_

*I*_440_(*t*): 1/4 of the integral value of the *d* proton in the ***Rh***_4_**1**_4_ square

*I*_dcb_(*t*): the integral value of the *f* proton of free dcb^–^ (before 1 h) or 1/2 of the integral value of the *e* proton of free dcb^–^ (after 1 h)

#### QASAP for the Rh_4_2_4_ square

*I*_L_(*t*): 1/4 of the integral value of the *e* proton in free ligand **2**^2–^

*I*_M_(*t*): 1/2 of the integral value of the *f* proton of dcb^–^ in ***Rh***(dcb)_2_

*I*_440_(*t*): 1/4 of the integral value of the *d* proton in the ***Rh***_4_**2**_4_ square

*I*_dcb_(*t*): the integral value of the *g* proton of free dcb^–^

*I*_M_(0) was determined based on the exact concentration of solution **A** determined by ^1^H NMR and the exact volume of solution **A** added into tube **II**.

*I*_L_(0) was determined by ^1^H NMR measurement before the addition of solution **A** into tube **II**.

### Existence ratio of *Rh*(dcb)_2_

As the total (initial) amount of ***Rh***(dcb)_2_ corresponds to *I*_M_(0), the existence ratio of ***Rh***(dcb)_2_ at *t* is expressed by *I*_M_(*t*)/*I*_M_(0).

### Existence ratio of L^2–^

As the total amount of free ligand L^2–^ corresponds to *I*_L_(0), the existence ratio of L^2–^ at *t* is expressed by *I*_L_(*t*)/*I*_L_(0).

### Existence ratio of the *Rh*_4_L_4_ square

As the total amount of the ***Rh***_4_L_4_ square is quantified based on L^2–^, the existence ratio of the ***Rh***_4_L_4_ square at *t* is expressed by *I*_440_(*t*)/*I*_L_(0).

### Existence ratio of dcb^–^

As the total amount of dcb^–^ corresponds to *I*_M_(0), the existence ratio of dcb^–^ at *t* is expressed by *I*_dcb_(*t*)/*I*_M_(0).

### Existence ratio of the total intermediates not observed by ^1^H NMR (Int)

The existence ratio of the total intermediates not observed by ^1^H NMR (Int) is determined based on the amount of L^2–^ in Int. Thus, the existence ratio of Int is calculated by subtracting the other species containing L^2–^ (free L^2–^, and the ***Rh***_4_L_4_ square) from the total amount of L^2–^ (*I*_L_(0)). The existence ratio of Int at *t* is expressed by (*I*_L_(0) – *I*_L_(*t*) – *I*_440_(*t*))/*I*_L_(0).

**〈** ***a*** **〉**

The initial amount of ***Rh*** centers corresponds to *I*_M_(0)/2.

The amount of ***Rh*** centers in ***Rh***(dcb)_2_ at *t* corresponds to *I*_M_(*t*)/2.

The amount of ***Rh*** centers in the ***Rh***_4_L_4_ square at *t* corresponds to *I*_440_(*t*).

The amount of ***Rh*** centers in Int at *t* is thus expressed by *I*_M_(0)/2 – *I*_M_(*t*)/2 – *I*_440_(*t*).

**〈** ***b*** **〉**

The initial amount of L^2–^ corresponds to *I*_L_(0).

The amount of free L^2–^ at *t* corresponds to *I*_L_(*t*).

The amount of L^2–^ in the ***Rh***_4_L_4_ square at *t* corresponds to *I*_440_(*t*).

The amount of L^2–^ in Int at *t* is thus expressed by *I*_L_(0) – *I*_L_(*t*) – *I*_440_(*t*).

**〈** ***c*** **〉**

The total amount of dcb^–^ corresponds to *I*_M_(0).

The amount of free dcb^–^ at *t* corresponds to *I*_dcb_(*t*).

The amount of dcb^–^ in ***Rh***(dcb)_2_ at *t* corresponds to *I*_M_(*t*).

The amount of dcb^–^ in Int at *t* is thus expressed by *I*_M_(0) – *I*_dcb_(*t*) – *I*_M_(*t*).

The 〈*n*〉 and 〈*k*〉 values are determined with these 〈*a*〉, 〈*b*〉, and 〈*c*〉 values by Eqs. (1) and (2).

1 $$\left\langle n\right\rangle =\frac{2\left\langle a\right\rangle -\left\langle c\right\rangle }{\left\langle b\right\rangle }$$

2 $$\left\langle k\right\rangle =\frac{\left\langle a\right\rangle }{\left\langle b\right\rangle }$$

### NASAP of the *Rh*_4_2_4_ square

#### The reaction network model and the classification of the elementary reactions

For the numerical analysis of self-assembly process (NASAP), a reaction network for the self-assembly of the ***Rh***_4_L_4_ square from ***Rh***X_2_ (X: the leaving ligand) and L^2–^ (**2**^2–^) is constructed as follows. Starting from the substrates, that is, ***Rh***X_2_ and L^2–^, the reaction path is traced to form the possible intermediate chemical species with up to ten ditopic ligands (L^2–^). In this network, the ***Rh***_3_L_3_ triangle is also considered as intermediate species. With these procedures taken, it is found that the total of 34 molecular species (including both the substrates and the product themselves) construct a reaction network composed of 1,698 elementary reactions. It should be noted that some of those reactions have the forward and backward processes (see the classification of the reaction type given below). All the intermediates considered in this network model and a simplified reaction network are shown in Supplementary Fig. 11.

This reaction network turns out to be so large that it is impossible to assign an individual rate constant to each reaction and to search for the parameter in such a vast parameter space to fit the experimental results best. Therefore, we divided the whole elementary reactions into ten classes possessing similar characteristics and defined rate constants as follows:

i. Incorporation of free L^2–^ by ***Rh***X_2_ with releasing a leaving ligand X.

*k*_1_ [min^–1^ M^–1^] and *k*_–1_ [min^–1^ M^–1^] for forward and backward reactions, respectively.

ii. Reaction of ***Rh***X_2_ and the second site of L^2–^, whose opposite site is already bonded with another ***Rh***.

*k*_2_ [min^–1^ M^–1^] and *k*_–2_ [min^–1^ M^–1^].

iii. Reaction of free L^2–^ and the second site of ***Rh***, which is already coordinated by another L^2–^.

*k*_3_ [min^–1^ M^–1^] and *k*_–3_ [min^–1^ M^–1^].

iv. Reaction at the second sites of L^2–^ and ***Rh***, both of whose other sites are already bonded with ***Rh*** and L^2–^, respectively.

*k*_4_ [min^–1^ M^–1^] and *k*_–4_ [min^–1^ M^–1^].

v. L-L exchange reaction. This reaction can lead to both the growth and the decomposition of oligomers, or produce no net change in the reaction system.

*k*_5_ [min^–1^ M^–1^].

vi. Cyclization to make a square via L-X exchange.

*k*_6_ [min^–1^] and *k*_–6_ [min^–1^ M^–1^].

vii. Cyclization to make a square via L-L exchange.

*k*_7_ [min^–1^] and *k*_–7_ [min^–1^ M^–1^].

viii. Cyclization to make a triangle via L-X exchange.

*k*_8_ [min^–1^] and *k*_–8_ [min^–1^ M^–1^].

ix. Cyclization to make a triangle via L-L exchange.

*k*_9_ [min^–1^] and *k*_–9_ [min^–1^ M^–1^].

x. X-X exchange, which practically produces no change in the reaction system.

*k*_10_ [min^–1^ M^–1^].

Typical examples of each type of elementary reaction are shown in Supplementary Fig. 12. Note that reactions in classes (i)–(v) and (x) are the intermolecular ligand exchanges and those in classes (iv)–(ix) are intramolecular ones. It should be noted here that each rate constant is defined per reaction site, based on the above modeling procedure. Therefore, the actual reaction rate for each reaction is estimated as the defined constant multiplied by the total number of available combinations. For example, for the reaction between ***Rh***X_2_ and L^2–^ to produce [***Rh***LX]^–^ and X, the rate constant is given as *k*_1_ times 2 (the number of ***Rh***–X bonds in ***Rh***X_2_) times 2 (the number of coordination sites in L^2–^), i.e.,

$${{{{{\boldsymbol{Rh}}}}}}{{{{{{\rm{X}}}}}}}_{2}+{{{{{{\rm{L}}}}}}}^{2{{{-}}}}\mathop{\to }\limits^{4{k}_{1}}{\left[{{{{{\boldsymbol{Rh}}}}}}{{{{{\rm{LX}}}}}}\right]}^{{{{-}}}}+{{{{{{\rm{X}}}}}}}^{{{{-}}}}$$

It was adopted this setting to explicitly distinguish the structural difference among the species with the same composition.

#### Numerical fitting of the rate constants

In order to numerically track the time evolution of the existence ratios for both reactants and products and the (〈*n*〉, 〈*k*〉) values, we have adopted a stochastic approach based on the chemical master equation, the so-called Gillespie algorithm. In this algorithm, for all the possible *N* chemical reactions including molecular species *S*_*ai*_, *S*_*bi*_, *S*_*ci*_, *…*,

$${S}_{{ai}}+{S}_{{bi}}+{{\cdot \cdot \cdot }}\to {S}_{{ci}}+{{{{{\rm{\cdot \cdot \cdot }}}}}}\left(i=1,\ldots ,N\right)$$

the total reaction rate *R*_tot_ is calculated as

$${R}_{{{{{{\rm{tot}}}}}}}={r}_{1}+{r}_{2}+{{\cdot \cdot \cdot }}+{r}_{N}\left(i=1,\ldots ,N\right)$$

$${r}_{i}={k}_{i}\left[{S}_{{ai}}\right]\left[{S}_{{bi}}\right]{{\cdot \cdot \cdot }}$$

Starting from the initial time *t* = 0, at each instant *t*, which one of the reactions to occur is determined with the uniform random number *s*_1_ ∈ (0,1) as

if ${s}_{1}\le {r}_{1}/{R}_{{{{{{\rm{tot}}}}}}},$ then reaction 1 occurs,

if ${r}_{1}/{R}_{{{{{{\rm{tot}}}}}}} < \, {s}_{1}\le ({r}_{1}+{r}_{2})/{R}_{{{{{{\rm{tot}}}}}}}$, then reaction 2 occurs,

if $({r}_{1}+...+{r}_{N-1})/{R}_{{{{{{\rm{tot}}}}}}} < \, {s}_{1}\le 1$, then reaction N occurs.

Another uniform random number *s*_2_ ∈ (0,1) is independently given to fix the time incremental *dt* as

$${dt}={{{{\mathrm{ln}}}}}(1/{s}_{2})/{R}_{{{{{{\rm{tot}}}}}}}$$

Time is updated as *t* = *t* + *dt*, together with the update of the numbers of corresponding molecular species, i.e., 〈*S*_*ai*_〉 → 〈*S*_*ai*_〉 – 1, 〈*S*_*bi*_〉 → 〈*S*_*bi*_〉 – 1, 〈*S*_*ci*_〉 → 〈*S*_*ci*_〉 + 1, …. The reason why this approach traces the chemical reactions and actually works well is given in the literature in detail, along with the practical way to implement it^75–78^.

The program for fixing the rate constant values is handmade for only this purpose. In order to obtain one of the best parameter sets, the following practical procedures are taken:

First, the rate constant sets are defined in the form of *k*_*i*_ = 10 ^*mi*^. Starting from the initial guess value for each *m*_*i*_, time evolutions of the global self-assembly event are traced with giving each *m*_*i*_ a stepwise increment or decrement, searching for smaller values of the residual sum of squares (RSS) for the experimental data at hand.

Initial guess sets are given in several different ways for exploring as broad parameter space as possible. An example is the uniform one like (*m*_1_, *m*_2_, *m*_3_, …) = (0, 0, 0, …). In another guess, rate constants for the oligomerization are given 10 to 100 times larger values than others by intention, for the reason that those reactions (especially, the inclusion of the free multitopic ligand L) generally occur very fast at the initial stage of the global self-assembly.

As a result of the global search, most of *k*_*i*_’s settle into almost definite values, with others floating within relatively wide numerical ranges. From our numerical experiences, the latter constants do not largely affect the global reproduction of the experimental self-assembly process and the dominant reaction pathways. Therefore, for those rate constant parameters, representative values are picked up within the scope of our chemical intuition.

We have to admit that at the final point an arbitrariness occurs. And as the parameter search is performed in this procedure for giving a good fit to the experimental counterpart, we cannot calculate the amounts of statistics such as the standard deviation for the obtained rate constant values.

With the initial conditions (numbers of species), 〈***Rh***X_2_〉_0_ = 12,900, 〈**2**^2–^〉_0_ = 12,900, 〈others〉_0_ = 0, rate constant search was performed in an eighteen-dimensional parameter space (*k*_1_, *k*_–1_, *k*_2_, *k*_–2_, *k*_3_, *k*_–3_, *k*_4_, *k*_–4_, *k*_5_, *k*_6_, *k*_–6_, *k*_7_, *k*_–7_, *k*_8_, *k*_–8_, *k*_9_, *k*_–9_, *k*_10_). The Avogadro number and the volume of the simulation box were set to be *N*_A_ = 6.0 × 10^23^ and *V* = 2.5 × 10^–17^ L, respectively, which give the same concentration as the experiments were carried out under [**2**^2–^]_0_ = [***Rh***]_0_ = 0.86 mM.

Because ^1^H NMR signals of the ***Rh***_3_L_3_ triangle were not observed during the self-assembly (Supplementary Figs. 8 and 10) and because the conversion of the ***Rh***_3_L_3_ triangle into the ***Rh***_4_L_4_ square was slow (Supplementary Fig. 12), the rate constant values concerning the trimerization (*k*_8_ and *k*_9_) should be zero. Indeed, any good parameter set of the rate constants with large *k*_8_ and *k*_9_ was not found.

After the rate constant search was finished, refined simulations were performed for some rate parameter sets that give existence ratios and the (〈*n*〉, 〈*k*〉) plot in good agreement with the experimental counterparts. The adequacy of the fitting to the experimental data was evaluated from the residual sum of squares (RSS) with the average of the experimental data, obtained from three runs. For all the time steps *t*_*i*_ at which the experimental data of existence ratios *R*_*ex*_^*S*^ and parameters *n*_ave_ and *k*_ave_ are available, RSS’s are calculated with the numerically obtained values *R*_*nu*_^*S*^ as (note that *S* = ***Rh***X_2_, **2**^2–^, ***Rh***_4_**2**_4_, or X),

$${{{{{\rm{RS}}}}}}{{{{{{\rm{S}}}}}}}_{1}=\mathop{\sum}\limits _{{t}_{i}} \mathop{\sum}\limits _{S}{\left({R}_{{nu},{t}_{i}}^{S}-{R}_{{ex},{t}_{i}}^{S}\right)}^{2}$$

$${{{{{\rm{RS}}}}}}{{{{{{\rm{S}}}}}}}_{2}=\mathop{\sum}\limits _{{t}_{i}}{\left({\left\langle n\right\rangle }_{{nu},{t}_{i}}-{\left\langle n\right\rangle }_{{ex},{t}_{i}}\right)}^{2}+\mathop{\sum}\limits _{{t}_{i}}{\left({\left\langle k\right\rangle }_{{nu},{t}_{i}}-{\left\langle k\right\rangle }_{{ex},{t}_{i}}\right)}^{2}$$

A representative numerical result and the corresponding rate constant set are shown in Figs. 3d and 3e and Supplementary Table 3, respectively. In the simulation to obtain Fig. 3d, e, the initial particles and the volume of the simulation box were set to be a thousand times larger than the rough parameter search, i.e., 〈***Rh***X_2_〉_0_ = 1,290,000, 〈**2**^2–^〉_0_ = 1,290,000, and *V* = 2.5 × 10^–15^ L. Although the numerical results of a single run are shown in Fig. 3d, e, a similar behavior was confirmed with several runs for the particle numbers given above.

#### Determination of the major self-assembly pathway

In the framework of the stochastic algorithm employed in this study, it is possible to count the number of occurrences (we call it as “frequency”) of each elementary reaction in the course of the self-assembly process. Therefore, the dominant reaction pathway as shown in Fig. 3f was determined by tracing back the reaction network from the objective product, ***Rh***_4_**2**_4_ in this case, to the substrates along the elementary reactions whose frequencies are larger than the others. For the elementary reaction with forward and backward processes, which direction to proceed is determined by the number difference between the forward and the backward reaction frequencies. Top 10 elementary reactions with high net frequency in the self-assembly of the ***Rh***_4_**2**_4_ square are listed in Supplementary Table 4.

The advantage of using the reaction frequency is that the main intermediates participating in the dominant self-assembly pathway are not missed with the reason of the existence ratio being small due to high reaction rate values associated with that species. For example, the existence ratio of (4,4,1) is very low compared with other Type II intermediates (Supplementary Fig. 13, so if the major self-assembly pathway is determined by connecting the species with large existence ratio, (4,4,1) is not involved in the major pathway. On the other hand, the net reaction frequency of the cyclization of (4,4,1) to form (4,4,0) is higher than those of the other elementary reactions to produce (4,4,0) (Supplementary Table 4), indicating that the square (4,4,0) is mainly produced by the cyclization of (4,4,1). The reason for the low existence ratio of (4,4,1) in the self-assembly is because of fast consumption of (4,4,1) by the cyclization. As this example shows, the determination of the major self-assembly pathway based on the existence ratios of the species leads to a wrong conclusion.

#### X-ray crystallographic structural analysis of the [Rh_4_1_4_(dmso-S)_4_]_2_ dimer

A single crystal was immersed in and coated with perfluoropolyalkylether (viscosity 80 cSt; abcr GmbH), and then mounted on a MicroMount^TM^ (MiteGen LLC). Diffraction data of the single crystal were collected on a VariMax Dual single crystal X-ray diffractometer with PILATUS 200 K detector (Rigaku Corporation) at 93(2) K, using Mo *K*α (λ = 0.71073 Å) radiation monochromated by multilayer mirror optics. Bragg spots were integrated using the CrysAlisPro program package (Rigaku Corporation). An empirical absorption correction based on the multi-scan method using spherical harmonics was implemented in the SCALE3 ABSPACK scaling algorithm. The structure was solved by an intrinsic phasing method on the SHELXT program^79^ and refined by a full-matrix least-squares minimization on *F*^2^ executed by the SHELXL program^80^, using Olex2 software package (OlexSys Ltd)^81^. Thermal displacement parameters were refined anisotropically for all non-hydrogen atoms. All the hydrogen atoms except for those on C70, C100, C137, C145 of terminal methoxy groups were located at calculated positions and the parameters were refined with a riding model. The data were corrected for disordered electron density of crystal solvents in void spaces by using the PLATON SQUEEZE method^82^. The crystal structure is shown in Fig. 4b. Crystallographic data of [***Rh***_4_**1**_4_(dmso-*S*)_4_]_2_ are summarized in Supplementary Table 7 and Supplementary Data 2.

### Supplementary information

Peer Review File

Supplementary Information

Description of additional supplementary files

Supplementary data 1

Supplementary data 2

## Data Availability

The data supporting the findings of this study are available within the article and its Supplementary Information and from the corresponding author upon reasonable request. The ^1^H and ^13^C{^1^H} NMR spectra of all substrates are available in Supplementary Data 1. The X-ray crystallographic coordinates for the structure reported in this Article have been deposited at the Cambridge Crystallographic Data Centre (CCDC), under deposition number CCDC 2223675 ([***Rh***_4_**1**_4_(dmso-*S*)_4_]_2_). The data can be obtained with reference to Supplementary Data 2 or free of charge from The Cambridge Crystallographic Data Centre via www.ccdc.cam.ac.uk/data_request/cif.

## Code availability

All the numerical results shown in the present article were obtained with homemade program codes written in Fortran90, whose essential parts except the elementary reactions in specific network structures have already been given as the supplementary^3^. Source codes and the associated data are always available upon reasonable request to one of the authors (S.T.).
