# Supplementary material for: Pathway selection in the self-assembly of Rh4L4 coordination squares under kinetic control
Source: Commun Chem. 2023 Nov 15;6:248. doi: 10.1038/s42004-023-01053-7 (PMC10651846; doi:10.1038/s42004-023-01053-7)
Supplement: Supplementary file 2 — Supplementary Information [file 42004_2023_1053_MOESM2_ESM.pdf]

Supplementary Information

Pathway selection in the self-assembly of Rh_4L_4 coordination squares under kinetic control

Atsushi Okazawa,[1†] Naoki Sanada,[2†] Satoshi Takahashi,[2] Hirofumi Sato,[3,4] and Shuichi Hiraoka[2,*]

[1]Department of Electrical Engineering and Bioscience, Waseda University, Tokyo 169-8555, Japan

[2]Department of Basic Science, Graduate School of Arts and Sciences, The University of Tokyo, Tokyo 153-8902, Japan

[3]Department of Molecular Engineering, Kyoto University, Kyoto 615-8510, Japan

[4]Fukui Institute for Fundamental Chemistry, Kyoto University, Kyoto 606-8103, Japan

†These authors contributed equally.

*Correspondence: hiraoka-s@g.ecc.u-tokyo.ac.jp

Contents

• Self-assembly from [***Rh***(CH_3CN)$_4$](BF_4)$_2$ and $\mathbf{1}^{2-}$ in CH_3CN ([$\mathbf{1}^{2-}$] = 10 mM) ······ 2
• Characterization of the ^{1}H NMR signals of the ***Rh***$_3$L$_3$ triangles and the ***Rh***$_4$L$_4$ squares ······ 3
• Mass spectrometry ······ 5
• Self-assembly from [***Rh***(CH_3CN)$_4$](BF_4)$_2$ and $\mathbf{2}^{2-}$ in CH_3CN or $CDCl_3$ ([$\mathbf{2}^{2-}$] = ca. 1 mM) ······ 7
• Model reaction of the ligand exchange of dcb$^-$ in ***Rh***(dcb)$_2$ with monotopic ligand ······ 9
• Self-assembly of the ***Rh***$_4$**2**$_4$ square from ***Rh***(dcb)$_2$ and $\mathbf{2}^{2-}$ in $CDCl_3$ ······ 10
  · QASAP for the ***Rh***$_4$**1**$_4$ square ······ 11
  · QASAP for the ***Rh***$_4$**2**$_4$ square ······ 14
• Numerical analysis of the self-assembly process (NASAP) of the ***Rh***$_4$**2**$_4$ square ······ 17
• Equilibration of a mixture of Rh(II)-triangular and square complexes by heat ······ 21
• Conversion of the ***Rh***$_3$**2**$_3$ triangle into the ***Rh***$_4$**2**$_4$ square assisted by dcb$^-$ ······ 22
• Supramolecular assembly of the ***Rh***$_4$**1**$_4$ square by solvophobic effect ······ 24
• X-ray crystallographic structural analysis of the [***Rh***$_4$**1**$_4$(dmso-*S*)$_4$]$_2$ dimer ······ 29

Self-assembly from [*Rh*($CH_3CN)_4](BF_4)_2$ and 1^{2-} in CH_3CN ([1^{2-}] = 10 mM)

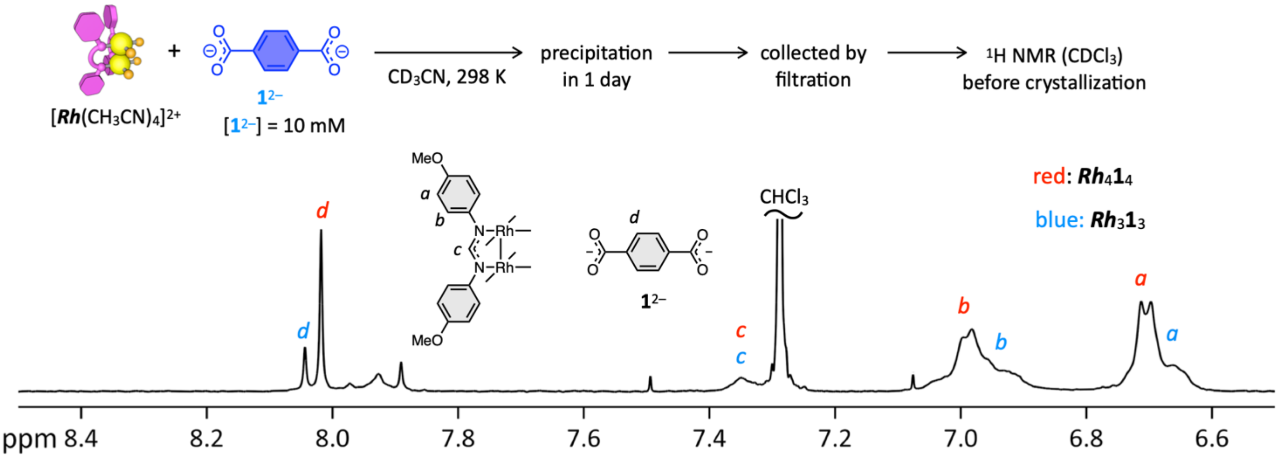

Supplementary Figure 1. A partial ^{1}H NMR spectrum (500 MHz, $CDCl_3$, 298 K, aromatic region) of the reaction mixture of the self-assembly from [*Rh*($CH_3CN)_4](BF_4)_2$ and **1**$^{2-}$ in CH_3CN ([**1**$^{2-}$] = 10 mM). Precipitation occurred in 1 day, then the solid material was collected by filtration and its ^{1}H NMR spectrum was measured in $CDCl_3$. The assignment of the signals of the ***Rh***$_3$**1**$_3$ triangle and the ***Rh***$_4$**1**$_4$ square was conducted by ^{1}H DOSY spectroscopy (Supplementary Figure 3) and by addition of pure ***Rh***$_4$**1**$_4$ square obtained by crystallization in a mixture of the triangle and the square in $CDCl_3$ (Supplementary Figure 2).

Characterization of the ¹H NMR signals of the *Rh*$_3$L$_3$ triangles and the *Rh*$_4$L$_4$ squares

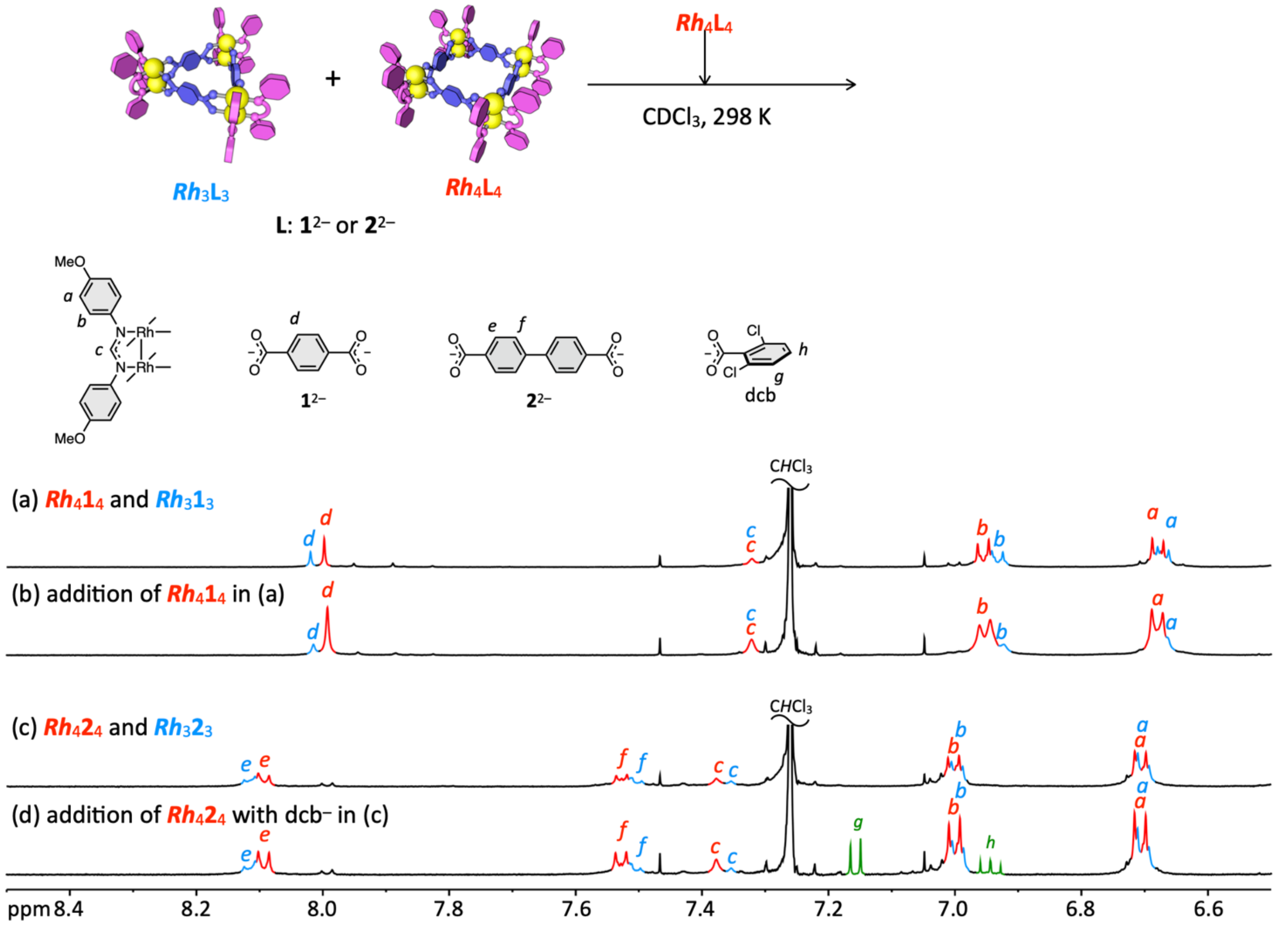

Supplementary Figure 2. Characterization of the signals of the triangle and the square by addition of pure square complex in a mixture of the triangle and the square. Because of the high symmetry of the ***Rh***$_3$L$_3$ triangle and the ***Rh***$_4$L$_4$ square, the signal patterns of the triangle and the square are the same. In addition, the chemical shift of the signals of the triangle and the square are affected by the coordination of the axial site of the Rh centers (solvent and/or the leaving ligand). To clearly assign the ^{1}H NMR signals in a mixture of the triangle and the square, a solution of the square, which was separately prepared (by crystallization for ***Rh***$_4$**1**$_4$ and by using dcb$^-$ as the leaving ligand for ***Rh***$_4$**2**$_4$), was added in a mixture of the triangle and the square. The ^{1}H NMR signals whose intensities were increased by addition of the square can be assigned to the square, while those without change can be assigned to the triangle. (a) ^{1}H NMR spectrum (500 MHz, CDCl$_3$, 298 K, aromatic region) of a mixture of the ***Rh***$_3$**1**$_3$ triangle and the ***Rh***$_4$**1**$_4$ square prepared from [***Rh***(CH$_3$CN)$_4$](BF$_4$)$_2$ and **1**$^{2-}$ in CDCl$_3$ at 298 K. (b) A CDCl$_3$ solution of crystals of the ***Rh***$_4$**1**$_4$ square was added in the solution of (a). The signals colored by red increased, which indicates that the signals colored in red and in blue are assigned to be the ***Rh***$_4$**1**$_4$ square and the ***Rh***$_3$**1**$_3$ triangle, respectively. This assignment of the signals is consistent with the ^{1}H DOSY spectrum of a mixture of the ***Rh***$_4$**1**$_4$ square and the ***Rh***$_3$**1**$_3$ triangle (Supplementary Figure 3). (c) ^{1}H NMR spectrum (500 MHz, CDCl$_3$, 298 K, aromatic region) of a mixture of the ***Rh***$_3$**2**$_3$ triangle and the ***Rh***$_4$**2**$_4$ square prepared from [***Rh***(CH$_3$CN)$_4$](BF$_4$)$_2$ and **2**$^{2-}$ in CDCl$_3$ at 298 K. (d) A CDCl$_3$ solution of the ***Rh***$_4$**2**$_4$ square and dcb$^-$ was added in the solution of (c). The assignment of the signals of the ***Rh***$_3$**2**$_3$ triangle and the ***Rh***$_4$**2**$_4$ square was conducted in the same way.

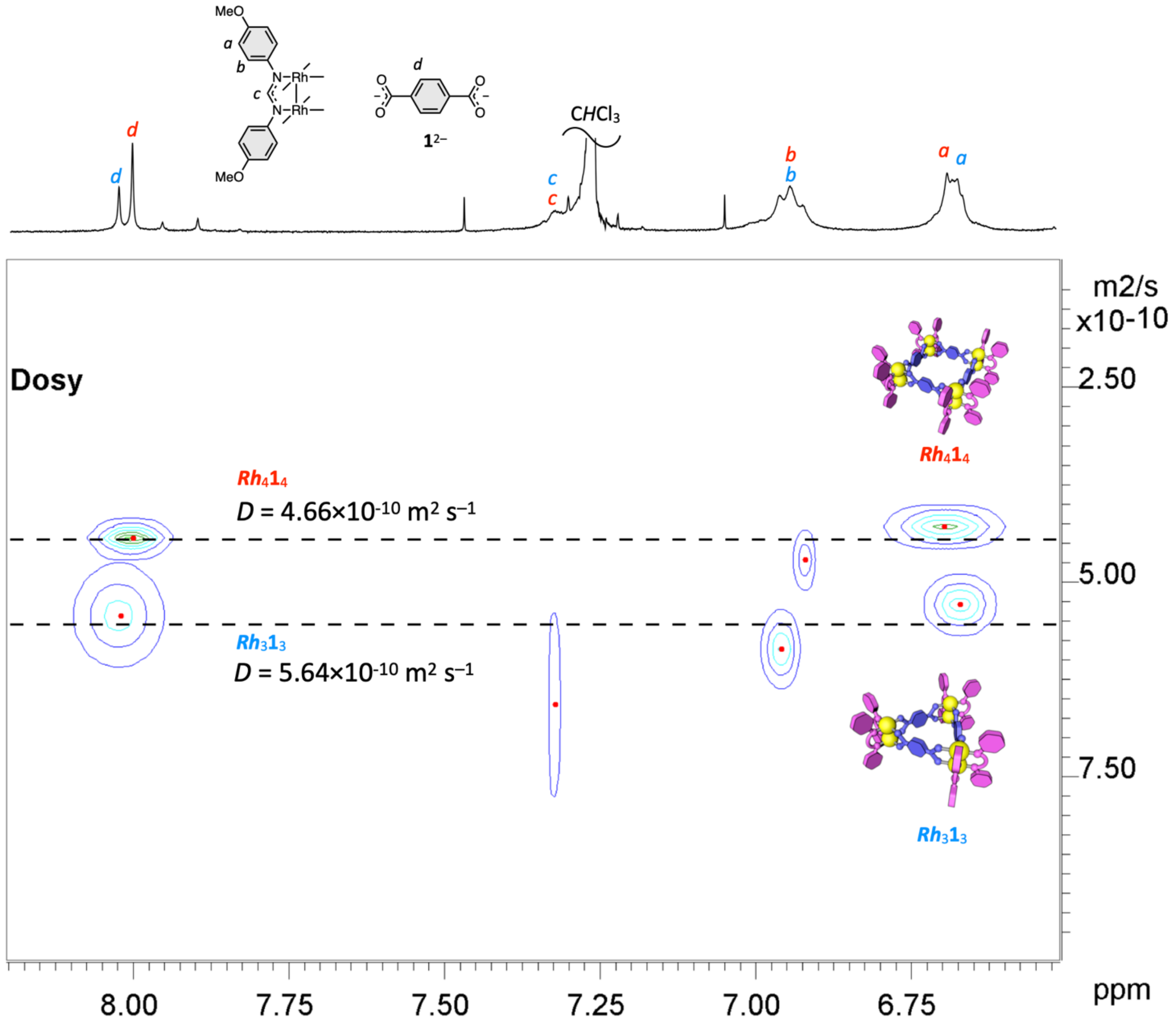

Supplementary Figure 3. ^{1}H DOSY spectrum (500 MHz, $CDCl_3$, 298 K, aromatic region) of a mixture of the ***Rh***$_3$**1**$_3$ triangle and the ***Rh***$_4$**1**$_4$ square prepared from [***Rh***(CH_3CN)$_4$](BF_4)$_2$ and **1**$^{2-}$ in $CDCl_3$. The assignment of the signals for the triangle and the square is consistent with the experimental result shown in Supplementary Figure 2a and 2b.

Mass spectrometry

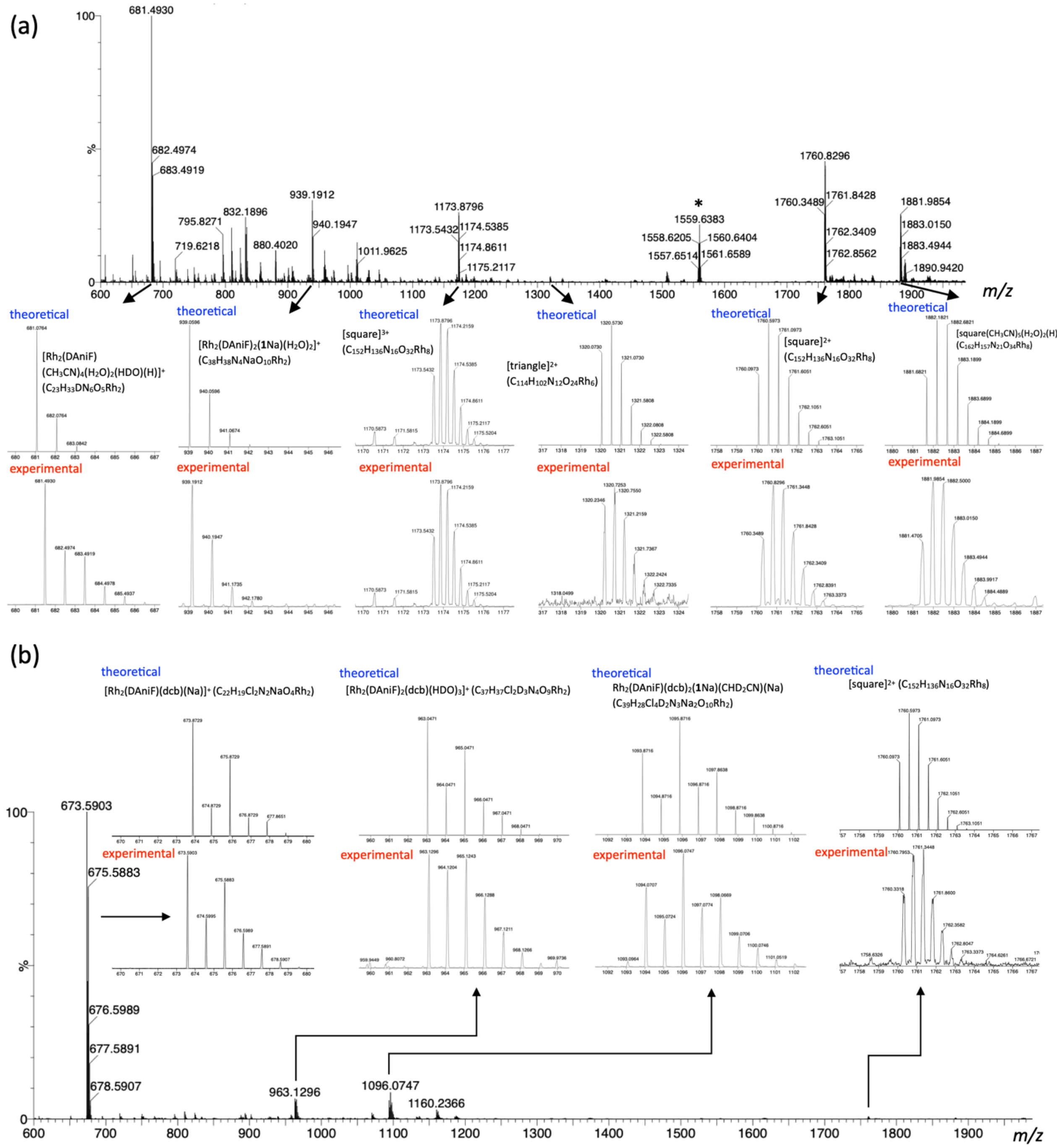

Supplementary Figure 4. ESI-TOF mass spectra of the Rh(II)-based self-assembly. (a) A solution of the self-assembly from [***Rh***$(CH_3CN)_4$]$(BF_4)_2$ and $\mathbf{1}^{2-}$ in $CDCl_3$ at 298 K after the convergence, whose ^{1}H NMR spectroscopy is shown in Figure 2a. The signals for the $\boldsymbol{Rh}_3\mathbf{1}_3$ triangle and the $\boldsymbol{Rh}_4\mathbf{1}_4$ square were detected. (b) A solution obtained after the conversion of $\boldsymbol{Rh}_3\mathbf{1}_3$ into $\boldsymbol{Rh}_4\mathbf{1}_4$ by dcb$^-$, whose ^{1}H NMR spectrum is shown in Figure 2b. Only the mass signal for $\boldsymbol{Rh}_4\mathbf{1}_4$ was detected. In all the measurements, the reaction mixture was diluted with $CHCl_3$ and CH_3CN (3:5, v/v) with addition of AcOH, filtered through a membrane filter (pore size: 0.20 μm), and injected in the mass spectrometer (Measurement condition: Capillary / 4.0 kV; Sampling Cone / 40 V; Source Offset / 80 V; Source / 80 °C; Desolvation / 150 °C; Cone Gas / 50 L h^{-1}; Desolvation Gas / 800 L h^{-1}) with 20.0 μL min^{-1} flow rate.

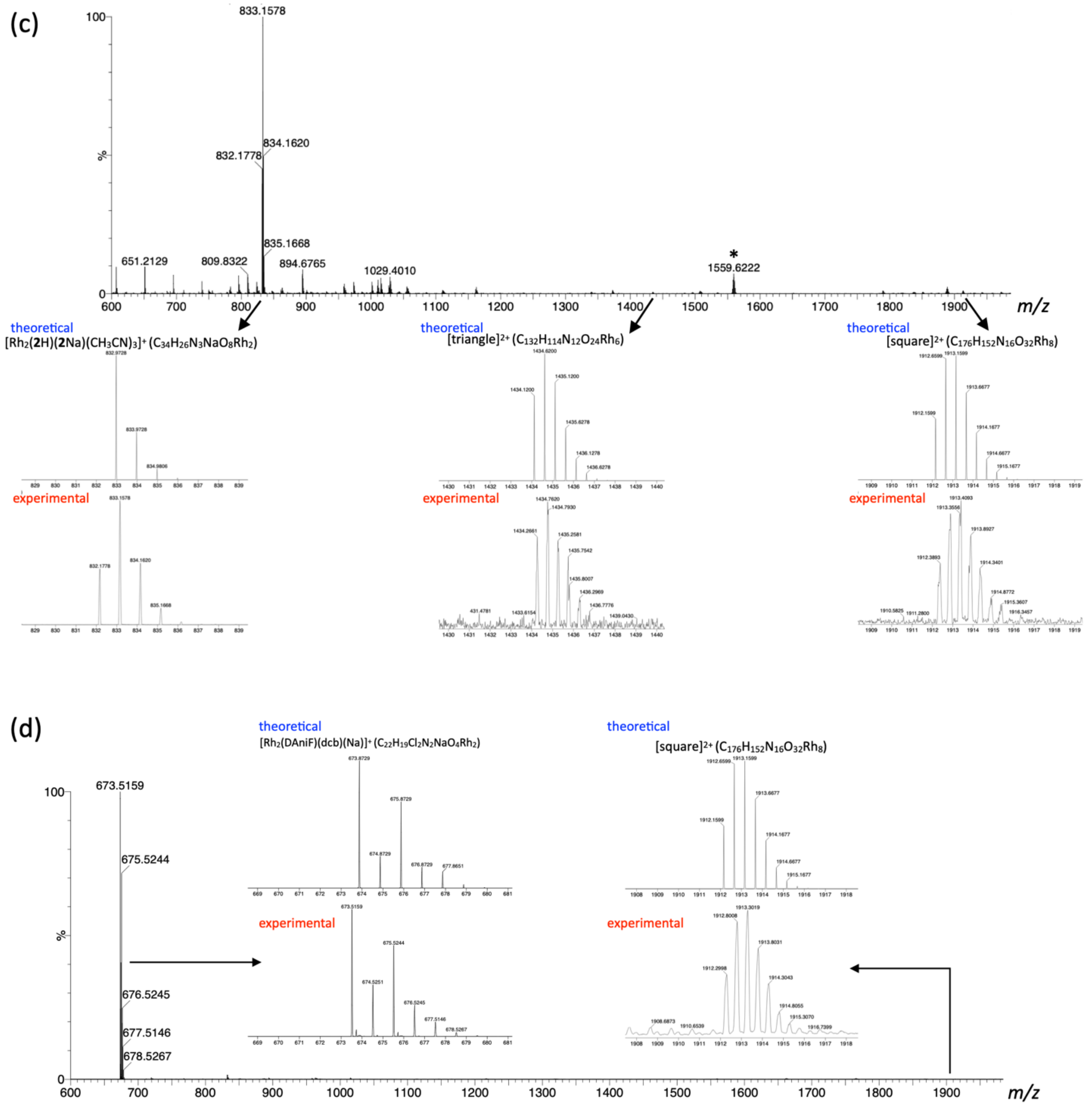

Supplementary Figure 4 (continued). (c) A solution of the self-assembly from [***Rh***(CH_3CN)$_4$](BF_4)$_2$ and **2**$^{2-}$ in $CDCl_3$ at 298 K after the convergence, whose ^{1}H NMR spectroscopy is shown in Supplementary Figure 5. The signals for the ***Rh***$_3$**2**$_3$ triangle and the ***Rh***$_4$**2**$_4$ square were detected. (d) A solution obtained after the conversion of ***Rh***$_3$**2**$_3$ into ***Rh***$_4$**2**$_4$ by dcb$^-$, whose ^{1}H NMR spectrum is shown in Supplementary Figure 15. Only the mass signal for ***Rh***$_4$**1**$_4$ was detected. The signals indicated by asterisks in (a) and (c), which have the same *m/z* and isotope pattern, appeared in different self-assembly from **1**$^{2-}$ and **2**$^{2-}$, indicating that this species does not contain the ditopic ligand.

Self-assembly from [Rh(CH_3CN)$_4$](BF_4)$_2$ and $2^{2–}$ in CH_3CN or $CDCl_3$ ([$2^{2–}$] = ca. 1 mM)

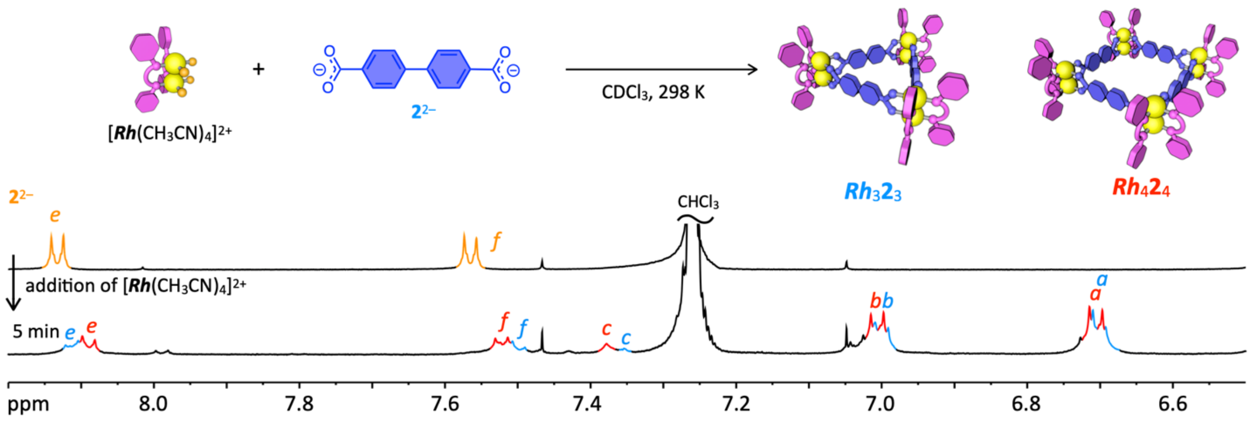

Supplementary Figure 5. The self-assembly from [***Rh***(CH_3CN)$_4$](BF_4)$_2$ and **2**$^{2–}$ in $CDCl_3$ at 298 K ([***Rh***]$_0$ = [**2**$^{2–}$]$_0$ = ca. 1 mM) monitored by ^{1}H NMR spectroscopy (500 MHz, $CDCl_3$, 298 K, aromatic region). The signals colored in red and blue indicate the ***Rh***$_3$**2**$_3$ triangle and the ***Rh***$_4$**2**$_4$ square, respectively. The ^{1}H NMR spectrum of **2**$^{2–}$ is shown for comparison.

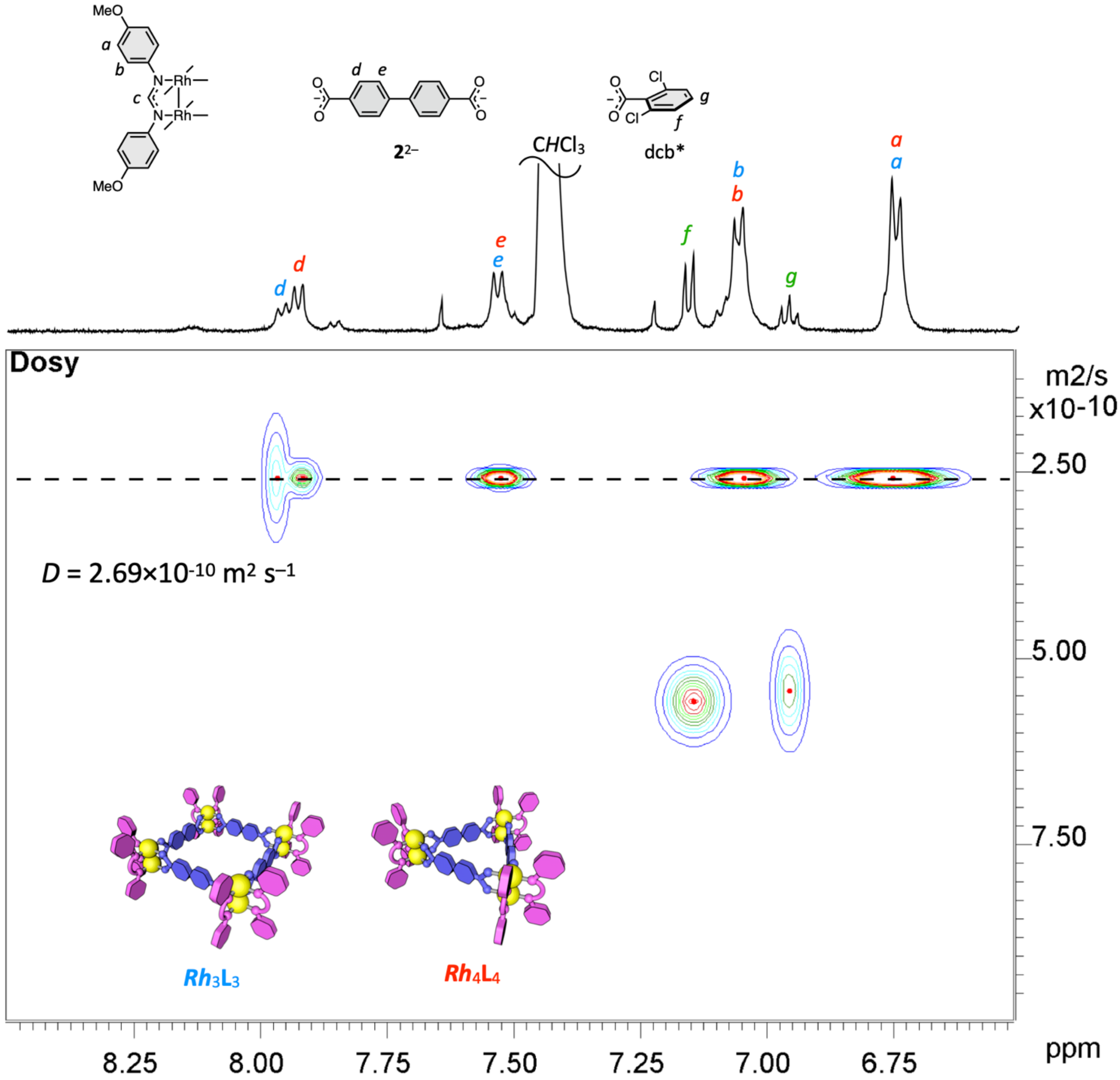

Supplementary Figure 6. ^{1}H DOSY spectrum (500 MHz, $CDCl_3$/DMSO-d_6 (9:1), 298 K, aromatic region) of a mixture of the ***Rh***$_3$**2**$_3$ triangle and the ***Rh***$_4$**2**$_4$ square prepared from [***Rh***(CH_3CN)$_4$](BF_4)$_2$ and **2**$^{2-}$ in $CDCl_3$/DMSO-d_6 (9:1) at 298 K. As the *D* values of the signals for the triangle and the square are very similar, which signals are assigned to the triangle and the square could not be determined by ^{1}H DOSY spectroscopy. Thus, we carried out the experiment where a solution of the square was added in a mixture of the triangle and the square (see Supplementary Figure 2c and 2d). The assignment of the ***Rh***$_3$**2**$_3$ triangle (blue) and the ***Rh***$_4$**2**$_4$ square (red) in the ^{1}H NMR spectrum (at around 8.0 ppm) is according to this experiment.

Model reaction of the ligand exchange of dcb⁻ in *Rh*(dcb)₂ with monotopic ligand

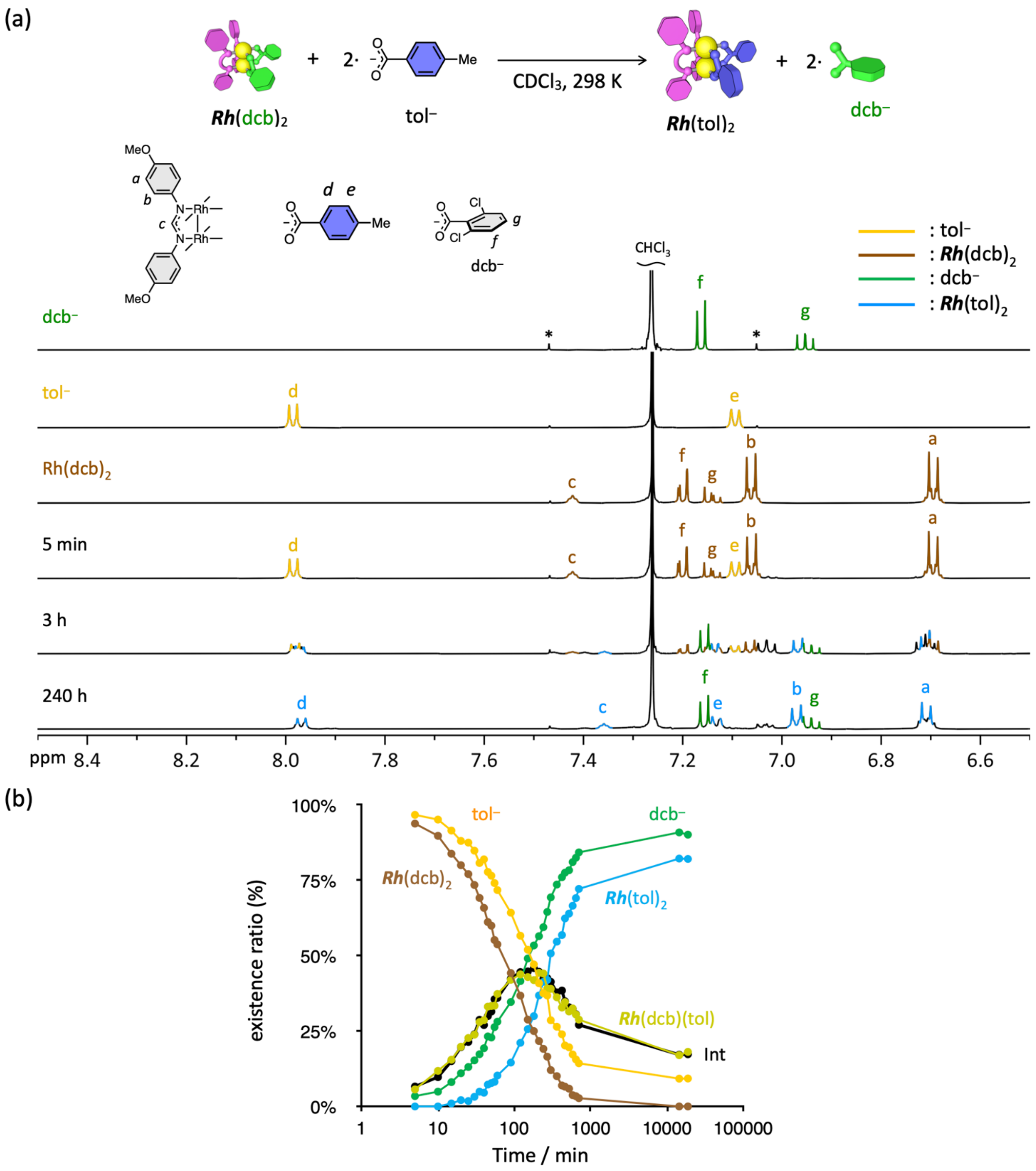

Supplementary Figure 7. (a) Monitoring of the ligand exchange of dcb⁻ in ***Rh***(dcb)$_2$ with *p*-toluate (tol⁻) in $CDCl_3$ at 298 K by ^{1}H NMR spectroscopy (500 MHz, $CDCl_3$, 298 K, aromatic region). Asterisks indicate the carbon satellite of $CHCl_3$. (b) The change in the existence ratios of the substrates, the products, and intermediates. Int indicates the total intermediate calculated by the difference between the existence ratios of ***Rh***(dcb)$_2$ and ***Rh***(tol)$_2$. As the existence ratio of Int and ***Rh***(dcb)(tol) are very similar, Int is mainly ***Rh***(dcb)(tol), indicating that reactions except the ligand exchanges did not occur.

Self-assembly of the Rh_4**2**$_4$ square from *Rh*(dcb)$_2$ and **2**$^{2-}$ in $CDCl_3$

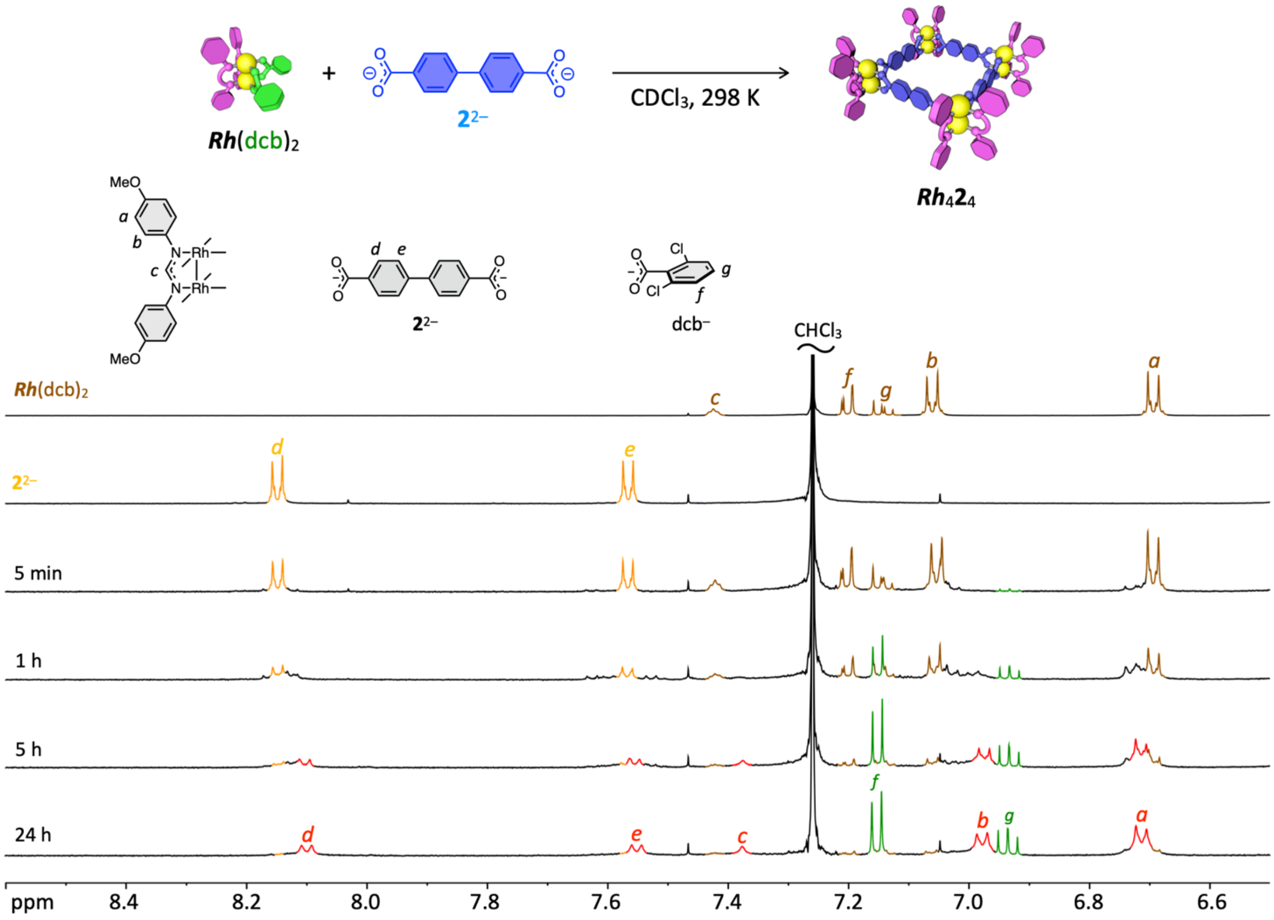

Supplementary Figure 8. Monitoring of the self-assembly of the ***Rh***$_4$**2**$_4$ square from ***Rh***(dcb)$_2$ and **2**$^{2-}$ in $CDCl_3$ at 298 K by ^{1}H NMR spectroscopy (500 MHz, $CDCl_3$, 298 K, aromatic region) ([***Rh***]$_0$ = [**2**$^{2-}$]$_0$ = 0.86 mM). The ^{1}H NMR spectra of ***Rh***(dcb)$_2$ and (n-Bu$_4$N)$_2$**2** are shown for comparison.

QASAP for the Rh_41_4 square

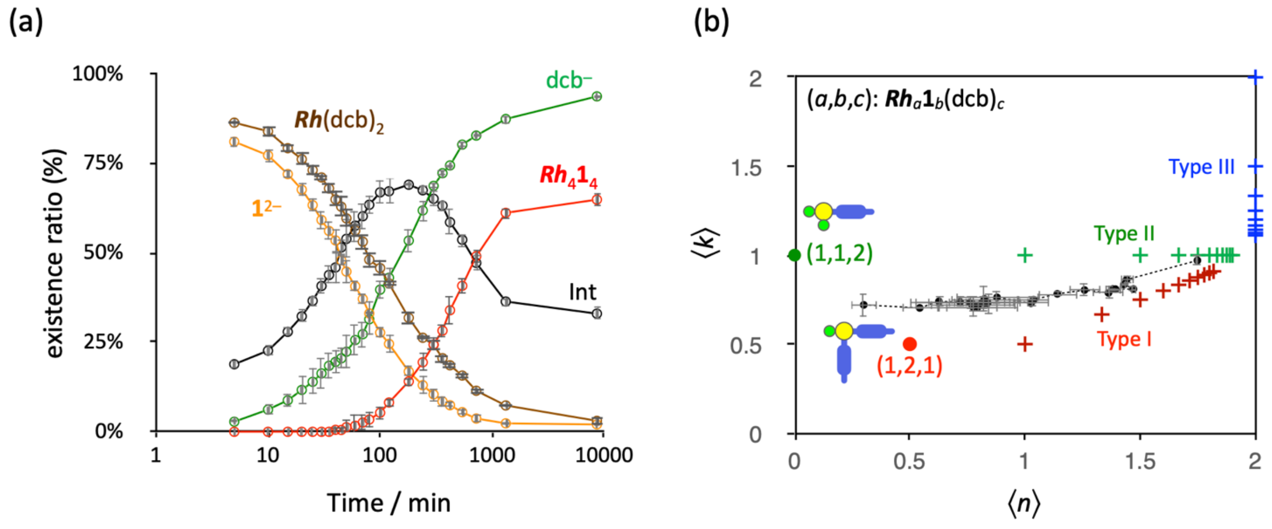

Supplementary Figure 9. QASAP data for the self-assembly of the ***Rh*$_4$1$_4$** square from ***Rh***(dcb)$_2$ and **1**$^{2-}$ in $CDCl_3$ at 298 K quantified by ^{1}H NMR spectroscopy ([***Rh***]$_0$ = [**1**$^{2-}$]$_0$ = 1.0 mM). (a) Existence ratios of the substrates (***Rh***(dcb)$_2$ and **1**$^{2-}$) and the products (the ***Rh*$_4$1$_4$** square and dcb$^-$). (b) An *n*-*k* plot. The data are shown as the average of the three runs of QASAP with standard errors. Red, green, and blue crosshairs indicate the (*n*, *k*) values of the Types I, II, and III chain intermediates, respectively. Red and green solid circles indicate (1,2,1) and (1,1,2), where the ***Rh***$^{2+}$ center has three carboxylate ligands two of which coordinate to a Rh(II) ion as a monodentate ligand. Basically, ***Rh***$^{2+}$ has two carboxylate ligands. In this case, the *n* value should be plotted in the range of $1 \leq n \leq 2$. However, the ⟨*n*⟩ values in the beginning of the self-assembly are smaller than 1. This is probably because more than two carboxylate ligands coordinate to the ***Rh***$^{2+}$. According to the (⟨*n*⟩, ⟨*k*⟩) values, (1,1,2) and (1,2,1), where one of the carboxylate ligands coordinates to a Rh center as a monodentate ligand to make a Rh–O bond, would be produced in the early stage of the self-assembly.

Supplementary Table 1. Time variation of $\mathbf{1}^{2-}$, ***Rh***(dcb)$_2$, the ***Rh***$_4$**1**$_4$ square, dcb$^-$, and Int; $\langle a \rangle$–$\langle c \rangle$ values of the average composition of the intermediates (***Rh***$_{\langle a \rangle}$**1**$_{\langle b \rangle}$(dcb)$_{\langle c \rangle}$); the ($\langle n \rangle$, $\langle k \rangle$) values for the self-assembly of the ***Rh***$_4$**1**$_4$ square from ***Rh***(dcb)$_2$ and $\mathbf{1}^{2-}$ ([***Rh***]$_0$ = [$\mathbf{1}^{2-}$]$_0$ = 1.0 mM) in $CDCl_3$ at 298 K.

Time / min	$\mathbf{1}^{2-}$ / %	***Rh***(dcb)$_2$ / %	***Rh***$_4$**1**$_4$ / %	dcb$^-$ / %	Int / %	$\langle a \rangle$	$\langle b \rangle$	$\langle c \rangle$	$\langle n \rangle$	$\langle k \rangle$
**The run 1**										
0	100	100	0	0	0	—	—	—	—	—
5	82.4	86.5	0	2.9	17.7	0.034	0.044	0.053	0.329	0.768
10	78.7	85.3	0	5.0	21.3	0.037	0.053	0.048	0.469	0.692
15	72.5	80.4	0	8.4	27.5	0.049	0.068	0.056	0.610	0.713
20	66.8	76.5	0	11.8	33.3	0.058	0.083	0.058	0.710	0.708
25	64.1	74.8	0	15.2	35.9	0.063	0.089	0.050	0.843	0.701
30	59.5	70.9	0	18.2	40.6	0.072	0.101	0.055	0.895	0.718
35	54.5	69.5	0	18.9	45.6	0.076	0.113	0.058	0.830	0.670
40	50.4	66.8	0	19.2	49.6	0.083	0.123	0.070	0.773	0.670
45	46.6	64.3	0	21.2	53.4	0.089	0.133	0.072	0.792	0.669
50	42.3	60.4	0	22.3	57.7	0.098	0.143	0.086	0.773	0.687
60	39.0	58.4	0	24.9	61.0	0.103	0.152	0.083	0.815	0.683
70	36.6	55.3	1.5	27.1	62.0	0.108	0.154	0.088	0.828	0.699
80	33.4	49.4	2.8	29.7	63.8	0.119	0.159	0.104	0.843	0.749
100	28.0	47.0	4.5	39.1	67.5	0.121	0.168	0.069	1.024	0.719
120	23.5	42.3	7.9	41.7	68.7	0.124	0.171	0.080	0.986	0.726
180	16.5	32.5	14.4	51.1	69.1	0.132	0.172	0.081	1.062	0.768
240	11.1	26.3	20.2	60.8	68.7	0.133	0.171	0.064	1.183	0.779
300	8.5	24.6	25.1	67.8	66.5	0.125	0.165	0.038	1.287	0.758
360	7.2	19.7	29.0	72.5	63.8	0.127	0.159	0.039	1.362	0.803
420	6.7	18.6	33.4	74.8	60.0	0.119	0.149	0.033	1.383	0.802
540	5.2	15.1	42.9	81.1	52.0	0.104	0.129	0.019	1.472	0.808
720	4.2	12.2	49.0	82.7	46.8	0.096	0.116	0.025	1.442	0.829
1320	2.1	7.0	62.7	88.6	35.2	0.075	0.088	0.022	1.470	0.861
8580	2.0	2.5	66.0	94.1	32.1	0.078	0.080	0.017	1.750	0.984
**The run 2**										
0	100	100	0	0	0	—	—	—	—	—
5	81.0	85.9	0	3.2	19.0	0.030	0.040	0.047	0.332	0.743
10	77.8	84.4	0	6.0	22.3	0.033	0.047	0.041	0.535	0.703
15	72.9	79.8	0	6.9	27.1	0.043	0.058	0.057	0.510	0.747
20	69.9	77.9	0	8.0	30.1	0.047	0.064	0.060	0.532	0.734
25	64.6	72.7	0	9.8	35.4	0.058	0.075	0.074	0.556	0.772
30	61.5	71.8	0	11.4	38.5	0.060	0.082	0.071	0.595	0.734
35	59.0	67.6	0	14.3	41.0	0.069	0.087	0.077	0.700	0.790
40	56.9	65.6	1.6	16.4	41.5	0.070	0.088	0.076	0.713	0.789
45	49.8	65.0	1.5	17.5	48.8	0.071	0.104	0.074	0.655	0.687

50	48.3	61.7	3.8	17.9	47.9	0.073	0.102	0.087	0.589	0.721
60	42.3	55.7	5.0	21.8	52.7	0.084	0.112	0.096	0.637	0.746
70	38.8	53.0	5.2	23.7	56.0	0.089	0.119	0.099	0.661	0.748
80	33.2	49.2	6.1	27.9	60.8	0.095	0.129	0.097	0.718	0.736
100	29.2	47.1	7.5	37.7	63.4	0.096	0.135	0.065	0.953	0.717
120	27.5	41.7	9.2	41.4	63.3	0.104	0.134	0.072	1.018	0.777
180	18.3	33.1	12.8	51.8	68.9	0.115	0.146	0.064	1.131	0.785
240	15.7	26.1	16.8	59.6	67.5	0.121	0.143	0.061	1.268	0.847
300	11.1	25.9	22.4	69.0	66.5	0.110	0.141	0.022	1.402	0.778
360	9.3	21.8	26.3	71.4	64.4	0.110	0.137	0.029	1.399	0.806
420	8.1	19.4	30.6	74.0	61.3	0.106	0.130	0.028	1.415	0.815
540	5.1	16.6	39.3	79.9	55.6	0.094	0.118	0.015	1.461	0.793
720	2.7	11.2	47.5	82.9	49.8	0.088	0.106	0.025	1.418	0.828
1320	2.3	7.9	61.0	87.2	36.8	0.066	0.078	0.021	1.430	0.847
8580	2.0	2.2	66.0	94.1	32.1	0.068	0.068	0.016	1.750	0.991
**The run 3**										
0	100	100	0	0	0	—	—	—	—	—
5	80.0	87.0	0	2.4	20.0	0.030	0.046	0.049	0.240	0.650
10	75.5	82.5	0	7.7	24.5	0.041	0.057	0.046	0.624	0.714
15	70.8	77.8	0	11.1	29.3	0.052	0.068	0.052	0.759	0.761
20	66.5	74.3	0	15.3	33.5	0.060	0.078	0.048	0.913	0.769
25	61.5	72.0	0	16.9	38.5	0.065	0.089	0.051	0.877	0.727
30	56.8	70.8	0	19.4	43.3	0.068	0.100	0.046	0.896	0.676
35	55.0	67.0	0	22.1	45.0	0.076	0.104	0.050	0.983	0.733
40	53.3	62.5	0	23.1	46.8	0.087	0.108	0.067	0.986	0.802
45	47.5	59.5	0	23.0	52.5	0.094	0.122	0.081	0.876	0.771
50	43.8	57.0	0	26.9	56.3	0.100	0.130	0.074	0.958	0.764
60	40.8	54.8	0	30.3	59.3	0.105	0.137	0.069	1.023	0.764
70	36.0	51.3	1.1	31.1	62.9	0.110	0.146	0.082	0.954	0.758
80	32.8	45.8	1.4	36.8	65.9	0.122	0.152	0.081	1.075	0.803
100	26.0	43.3	4.0	42.5	70.0	0.122	0.162	0.066	1.099	0.754
120	22.8	40.5	7.5	46.1	69.8	0.120	0.161	0.062	1.107	0.746
180	15.8	30.0	15.0	57.5	69.3	0.127	0.160	0.058	1.227	0.794
240	12.3	26.5	21.3	65.3	66.5	0.121	0.154	0.038	1.324	0.786
300	11.8	22.5	25.8	69.4	62.5	0.120	0.145	0.037	1.397	0.828
360	9.0	20.3	29.5	73.0	61.5	0.116	0.142	0.031	1.416	0.817
420	7.5	17.8	38.0	74.6	54.5	0.102	0.126	0.035	1.343	0.812
540	6.3	15.5	40.3	79.8	53.5	0.102	0.124	0.022	1.477	0.827
720	4.3	11.0	50.8	82.9	45.0	0.089	0.104	0.028	1.428	0.850
1320	2.8	7.3	60.0	86.5	37.3	0.076	0.086	0.029	1.423	0.879
8580	2.3	4.5	62.8	93.3	35.0	0.076	0.081	0.010	1.743	0.936

QASAP for the $Rh_4$2$_4$ square

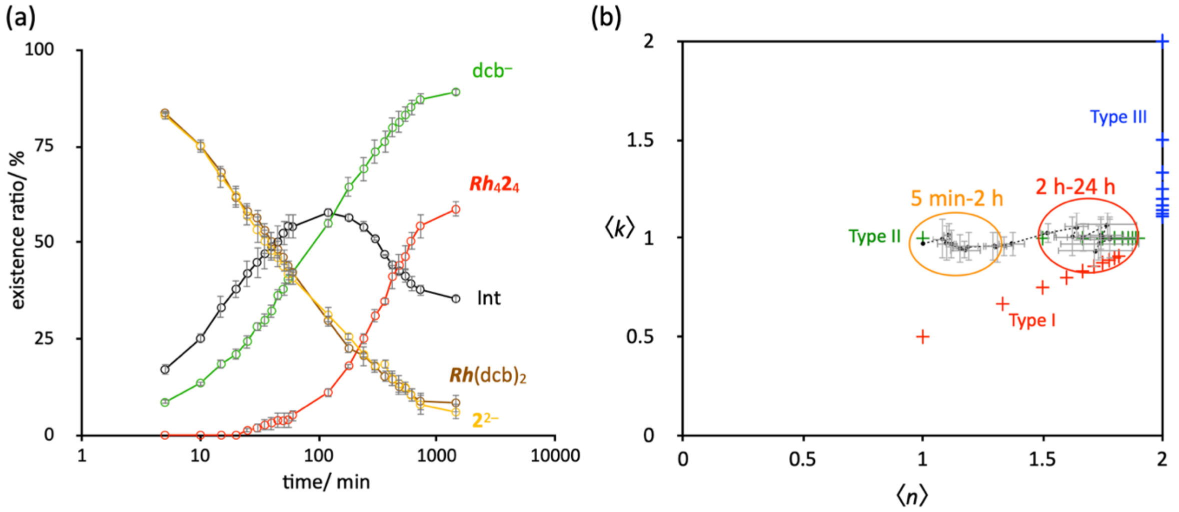

Supplementary Figure 10. QASAP data for the self-assembly of the ***Rh*$_4$2$_4$** square from ***Rh***(dcb)$_2$ and **2**$^{2-}$ in $CDCl_3$ at 298 K quantified by ^{1}H NMR spectroscopy ([***Rh***]$_0$ = [**2**$^{2-}$]$_0$ = 0.86 mM). (a) Existence ratios of the substrates (***Rh***(dcb)$_2$ and **2**$^{2-}$), the products (the ***Rh*$_4$2$_4$** square and dcb$^-$), and all the intermediates (Int). (b) An *n*-*k* plot. The data are shown as the average of the three runs of QASAP with standard errors. Red, green, and blue crosshairs indicate the (*n*, *k*) values of the Types I, II, and III chain intermediates, respectively.

Supplementary Table 2. Time variation of $\mathbf{2}^{2-}$, $\boldsymbol{Rh}$(dcb)$_2$, the $\boldsymbol{Rh}_4\mathbf{2}_4$ square, and dcb$^-$, Int; ⟨a⟩–⟨c⟩ values of the average composition of the intermediates ($\boldsymbol{Rh}_{\langle a\rangle}\mathbf{2}_{\langle b\rangle}(dcb)_{\langle c\rangle}$); the (⟨n⟩, ⟨k⟩) values for the self-assembly of the $\boldsymbol{Rh}_4\mathbf{2}_4$ square from $\boldsymbol{Rh}$(dcb)$_2$ and $\mathbf{2}^{2-}$ ([$\boldsymbol{Rh}$]$_0$ = [$\mathbf{2}^{2-}$]$_0$ = 0.86 mM) in $CDCl_3$ at 298 K.

Time / min	$\mathbf{2}^{2-}$ / %	$\boldsymbol{Rh}$(dcb)$_2$ / %	$\boldsymbol{Rh}_4\mathbf{2}_4$ / %	dcb$^-$ / %	Int / %	⟨a⟩	⟨b⟩	⟨c⟩	⟨n⟩	⟨k⟩
**The run 1**										
0	100	100	0	0	0	—	—	—	—	—
5	81.9	83.5	0.0	9.0	18.1	0.031	0.034	0.028	1.001	0.911
10	72.8	73.0	0.0	14.0	27.2	0.051	0.051	0.049	1.033	0.995
15	61.6	66.3	0.0	20.8	38.4	0.063	0.072	0.048	1.082	0.878
20	57.6	58.5	0.0	23.3	42.4	0.078	0.079	0.068	1.098	0.978
25	51.5	55.3	0.0	27.9	48.5	0.084	0.091	0.063	1.150	0.923
30	47.1	53.9	0.0	30.0	52.9	0.086	0.099	0.060	1.134	0.871
35	44.3	51.4	0.0	31.4	55.7	0.091	0.104	0.064	1.128	0.873
40	43.6	47.7	0.0	33.4	56.4	0.098	0.105	0.071	1.187	0.929
45	41.6	46.9	0.0	36.3	58.4	0.099	0.109	0.063	1.245	0.911
50	41.1	44.2	0.0	37.1	58.9	0.104	0.110	0.070	1.259	0.948
55	39.6	42.3	0.0	41.2	60.4	0.108	0.113	0.062	1.366	0.956
60	37.5	40.6	2.7	42.1	59.9	0.106	0.112	0.065	1.317	0.949
120	28.0	29.2	12.2	56.0	59.9	0.109	0.112	0.055	1.463	0.979
180	25.6	23.2	17.0	62.4	57.4	0.112	0.107	0.054	1.582	1.042
240	22.1	23.1	24.4	66.6	53.5	0.098	0.100	0.039	1.576	0.981
300	20.2	20.2	28.0	73.5	51.8	0.097	0.097	0.023	1.759	1.000
360	21.0	17.6	32.8	76.8	46.2	0.093	0.086	0.021	1.907	1.074
420	18.5	17.5	35.7	80.0	45.8	0.087	0.086	0.010	1.933	1.022
480	17.4	16.4	39.2	81.0	43.4	0.083	0.081	0.010	1.925	1.024
540	15.1	15.6	41.4	82.2	43.5	0.080	0.081	0.008	1.876	0.989
600	13.7	13.4	46.2	82.9	40.2	0.075	0.075	0.014	1.829	1.006
720	12.6	12.3	47.8	84.5	39.6	0.074	0.074	0.012	1.851	1.006
1440	8.2	12.1	55.3	87.6	36.5	0.061	0.068	0.001	1.766	0.892
**The run 2**										
0	100	100	0	0	0	—	—	—	—	—
5	81.5	83.2	0.0	9.0	18.5	0.030	0.033	0.028	0.974	0.908
10	75.2	78.0	0.0	14.1	24.8	0.039	0.044	0.028	1.139	0.886
15	68.0	69.9	0.0	17.9	32.0	0.054	0.057	0.043	1.121	0.943
20	63.0	66.3	0.0	20.5	37.0	0.060	0.066	0.047	1.107	0.909
25	59.3	62.0	1.9	22.6	38.8	0.064	0.069	0.055	1.070	0.931
30	54.5	60.4	3.1	28.7	42.4	0.065	0.075	0.039	1.209	0.860
35	52.1	56.6	4.5	31.3	43.4	0.069	0.077	0.043	1.234	0.896
40	49.0	54.6	5.5	34.1	45.5	0.071	0.081	0.040	1.260	0.876
45	47.2	52.1	6.1	38.8	46.7	0.074	0.083	0.032	1.398	0.894
50	44.4	49.9	6.4	40.8	49.2	0.078	0.087	0.033	1.399	0.888

55	41.0	46.9	6.8	42.8	52.2	0.082	0.093	0.037	1.377	0.887
60	40.4	44.2	7.5	43.8	52.1	0.086	0.092	0.042	1.395	0.927
120	30.7	32.1	12.5	56.1	56.8	0.098	0.101	0.042	1.536	0.975
180	24.2	24.5	18.7	69.2	57.0	0.101	0.101	0.022	1.771	0.995
240	20.3	23.1	23.2	75.1	56.5	0.095	0.100	0.006	1.837	0.950
300	17.5	18.7	31.5	78.6	51.0	0.088	0.091	0.009	1.846	0.975
360	17.0	15.5	35.9	80.8	47.0	0.086	0.084	0.013	1.910	1.033
420	11.8	15.1	46.5	84.1	41.7	0.068	0.074	0.003	1.807	0.921
480	9.6	11.9	49.7	86.3	40.7	0.068	0.072	0.007	1.795	0.944
540	10.3	11.9	52.0	87.0	37.7	0.064	0.067	0.004	1.854	0.958
600	9.7	10.8	54.6	89.0	35.6	0.061	0.063	0.001	1.929	0.971
720	7.6	9.4	57.3	89.4	35.1	0.059	0.062	0.004	1.828	0.949
1440	7.9	9.0	58.0	88.8	34.1	0.059	0.061	0.008	1.808	0.968
**The run 3**										
0	100	100	0	0	0	—	—	—	—	—
5	85.5	84.0	0.0	7.5	14.5	0.033	0.030	0.035	1.027	1.099
10	76.8	74.3	0.0	12.5	23.2	0.054	0.048	0.055	1.074	1.109
15	71.2	68.5	0.0	16.7	28.8	0.066	0.060	0.062	1.158	1.092
20	65.7	60.1	0.0	19.1	34.3	0.083	0.071	0.087	1.114	1.165
25	59.6	56.9	1.9	22.5	38.5	0.086	0.080	0.086	1.072	1.072
30	58.3	54.8	2.5	25.8	39.2	0.089	0.082	0.081	1.187	1.089
35	54.4	51.3	3.4	26.8	42.1	0.094	0.088	0.091	1.108	1.074
40	51.1	48.3	4.1	29.2	44.8	0.099	0.093	0.094	1.119	1.062
45	49.3	45.7	5.3	33.7	45.4	0.102	0.094	0.086	1.249	1.079
50	46.2	44.5	4.8	35.8	49.1	0.106	0.102	0.082	1.263	1.034
55	44.7	42.4	5.1	37.2	50.2	0.109	0.104	0.085	1.281	1.045
60	44.1	41.8	5.5	40.8	50.4	0.110	0.105	0.072	1.400	1.045
120	34.9	27.8	8.6	52.7	56.5	0.132	0.118	0.081	1.561	1.126
180	26.9	19.9	18.3	61.5	54.8	0.129	0.114	0.077	1.576	1.127
240	20.4	15.6	27.6	65.6	51.9	0.118	0.108	0.078	1.462	1.093
300	16.5	14.4	33.4	68.4	50.1	0.108	0.104	0.071	1.397	1.041
360	17.1	12.7	35.5	70.7	47.5	0.108	0.099	0.069	1.487	1.092
420	13.6	10.8	41.2	75.2	45.2	0.100	0.094	0.058	1.502	1.061
480	13.0	9.5	43.3	76.3	43.8	0.098	0.091	0.059	1.510	1.080
540	11.8	9.2	45.7	80.0	42.5	0.094	0.089	0.045	1.616	1.062
600	7.8	7.4	50.1	83.4	42.1	0.088	0.088	0.038	1.582	1.009
720	3.6	4.7	57.8	87.4	38.6	0.078	0.080	0.033	1.531	0.971
1440	1.9	4.1	62.5	90.7	35.6	0.069	0.074	0.022	1.585	0.939

Numerical analysis of the self-assembly process (NASAP) of the *Rh*$_4$2$_4$ square

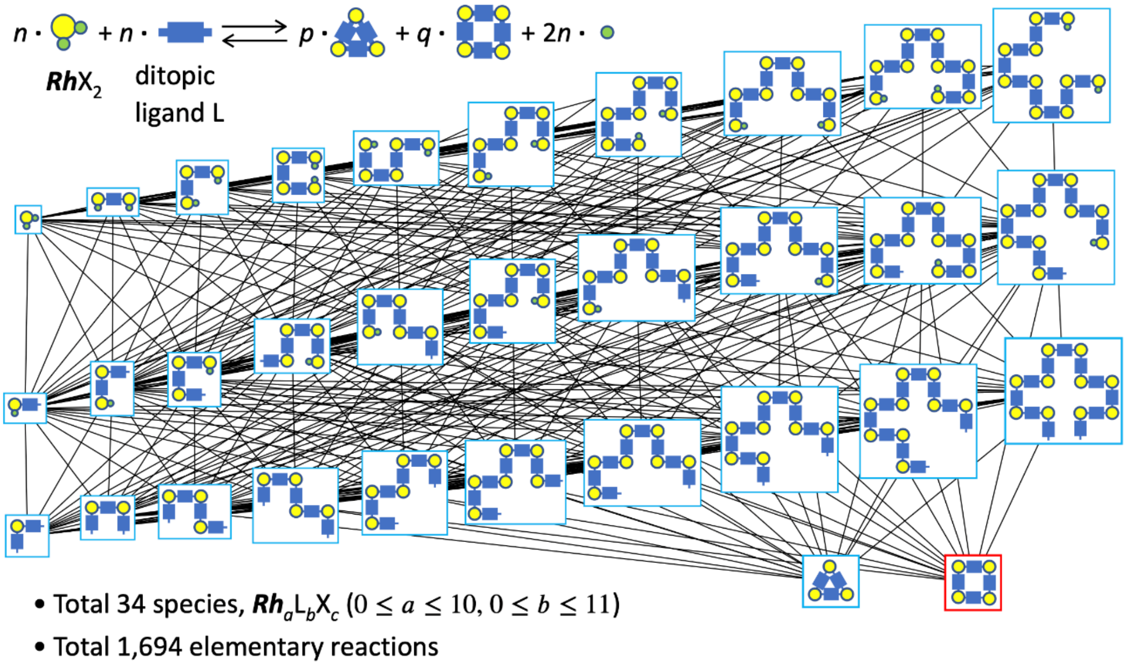

Supplementary Figure 11. Reaction network model of the self-assembly for the ***Rh***$_4$L$_4$ squares used in NASAP.

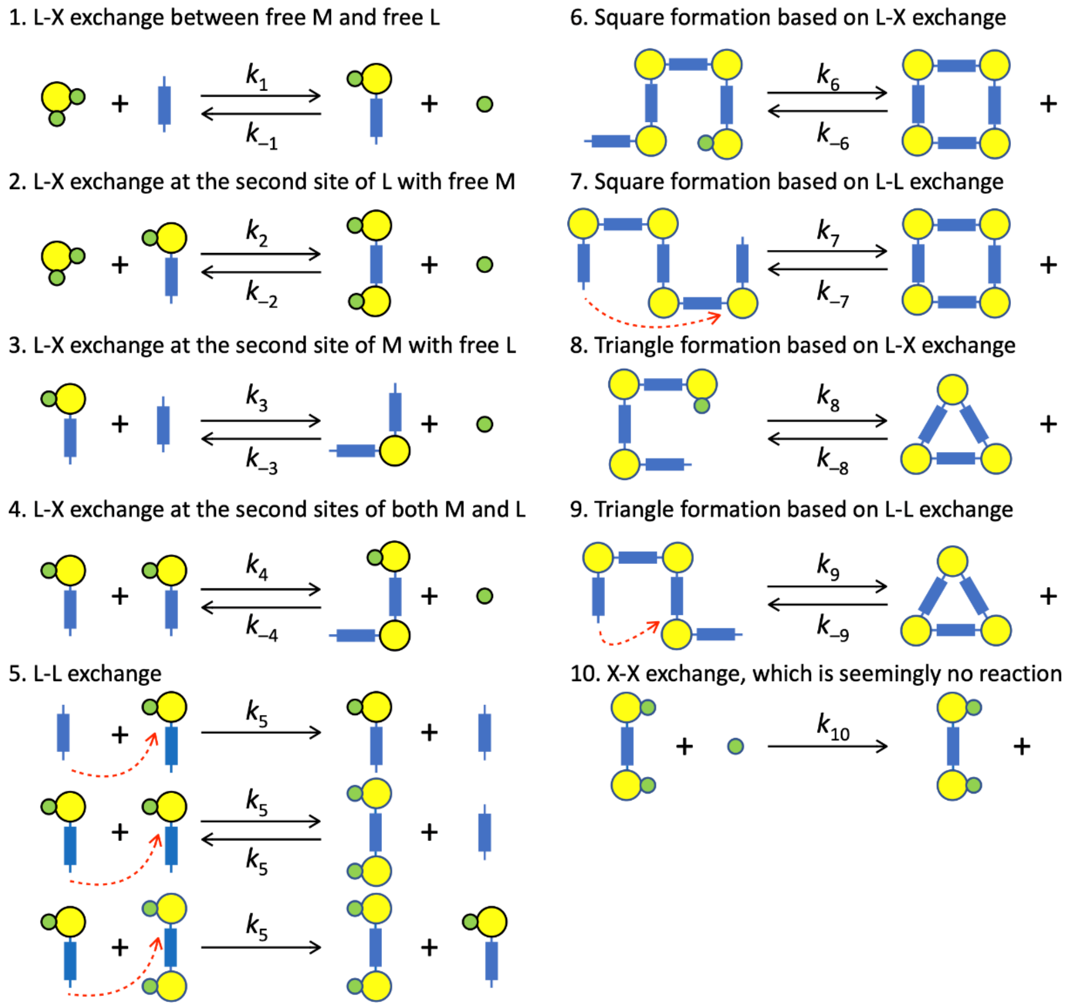

Supplementary Figure 12. Classification of the elementary reactions in the reaction network model shown in Supplementary Figure 11.

Supplementary Table 3. A list of the rate constants determined by NASAP.

Rate constant	Parameter value	unit
k_1	$10^{0.9}$	M^{-1} min^{-1}
k_{-1}	$10^{0.9}$	M^{-1} min^{-1}
k_2	$10^{0.2}$	M^{-1} min^{-1}
k_{-2}	$10^{-0.6}$	M^{-1} min^{-1}
k_3	$10^{0.1}$	M^{-1} min^{-1}
k_{-3}	$10^{-1.8}$	M^{-1} min^{-1}
k_4	$10^{1.5}$	M^{-1} min^{-1}
k_{-4}	$10^{-3.1}$	M^{-1} min^{-1}
k_5	$10^{-1.7}$	M^{-1} min^{-1}
k_6	$10^{-0.8}$	min^{-1}
k_{-6}	0.0	M^{-1} min^{-1}
k_7	$10^{-2.6}$	min^{-1}
k_{-7}	0.0	M^{-1} min^{-1}
k_8	0.0	min^{-1}
k_{-8}	0.0	M^{-1} min^{-1}
k_9	0.0	min^{-1}
k_{-9}	0.0	M^{-1} min^{-1}
k_{10}	$10^{-0.6}$	M^{-1} min^{-1}

Supplementary Table 4. Top 10 elementary reactions with high net frequency in the self-assembly of the ***Rh***$_4$**2**$_4$ square.

entry	elementary reaction[a]	net frequency[b]
1	(1,0,2) + (0,1,0) ⟶ (1,1,1) + (0,0,1)	1,105,451
2	(1,1,1) + (1,1,1) ⟶ (2,2,1) + (0,0,1)	283,880
3	(2,2,1) + (1,1,1) ⟶ (3,3,1) + (0,0,1)	138,249
4	(4,4,1) ⟶ (4,4,0) + (0,0,1)	100,291
5	(3,3,1) + (1,1,1) ⟶ (4,4,1) + (0,0,1)	63,843
6	(1,1,1) + (1,0,2) ⟶ (2,1,2) + (0,0,1)	60,579
7	(1,1,1) + (0,1,0) ⟶ (1,2,0) + (0,0,1)	50,494
8	(2,1,2) + (1,1,1) ⟶ (3,2,2) + (0,0,1)	28,386
9	(1,1,1) + (1,2,0) ⟶ (2,3,0) + (0,0,1)	27,498
10	(2,2,1) + (2,2,1) ⟶ (4,4,1) + (0,0,1)	21,205

[a](a,b,c) indicates ***Rh***$_a$**2**$_b$(dcb)$_c$.

[b]Net frequency is defined as (the total number of forward reactions) – (the total number of backward reactions) in the self-assembly.

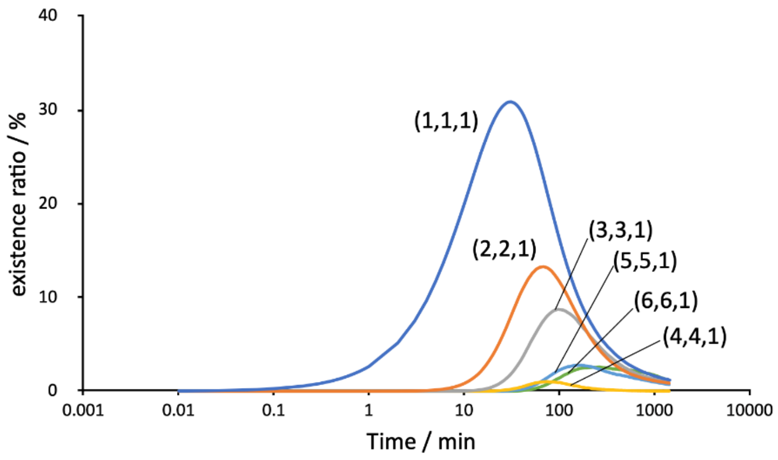

Supplementary Figure 13. Time-development of the existence ratios of the species in the major self-assembly pathway of the ***Rh***$_4$**2**$_4$ square. The change in the existence ratios of (5,5,1) and (6,6,1) is also indicated for comparison, though they are not in the major self-assembly pathway of the ***Rh***$_4$**2**$_4$ square (Figure 3f).

Equilibration of a mixture of Rh(II)-triangular and square complexes by heat

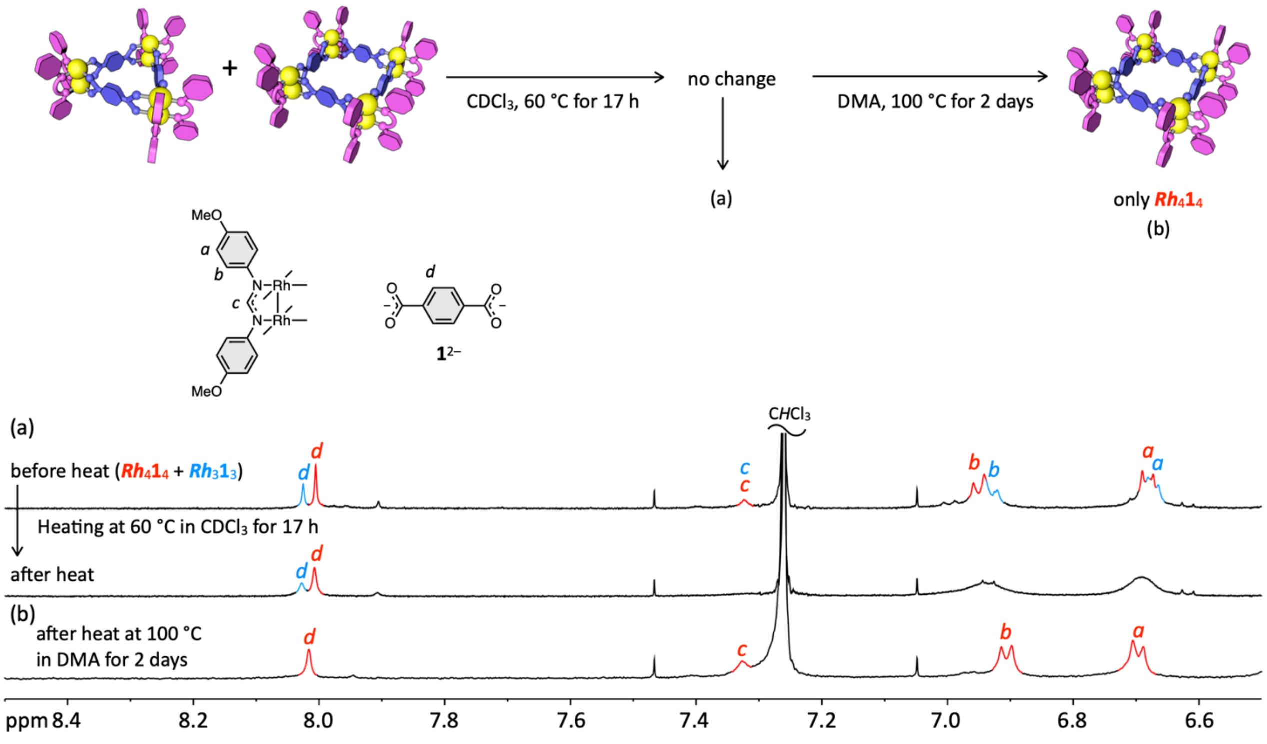

Supplementary Figure 14. Conversion of the ***Rh***$_3$**1**$_3$ triangle into the ***Rh***$_4$**1**$_4$ square by heating. (a) ^{1}H NMR spectrum of a mixture of the ***Rh***$_3$**1**$_3$ triangle and the ***Rh***$_4$**1**$_4$ square (500 MHz, $CDCl_3$, 298 K, aromatic region) after the self-assembly from [***Rh***(CH_3CN)$_4$](BF_4)$_2$ and **1**$^{2-}$ in $CDCl_3$ at 298 K and the one obtained after heating in $CDCl_3$ at 60 °C for 17 h. (b) The solvent of the mixture of the ***Rh***$_3$**1**$_3$ triangle and the ***Rh***$_4$***1***$_4$ square of (a) was replaced with dimethylacetamide (DMA) and the resulting solution was heated at 100 °C for 2 days. Then, solvent was replaced with $CDCl_3$ and its ^{1}H NMR spectrum was measured (500 MHz, $CDCl_3$, 298 K, aromatic region) to show only signals of the ***Rh***$_4$**1**$_4$ square. These results indicate that heating at 100 °C for 2 days is needed for the conversion and that the ***Rh***$_4$**1**$_4$ square is thermodynamically most stable.

Conversion of the *Rh*$_3$2$_3$ triangle into the *Rh*$_4$2$_4$ square assisted by dcb$^-$

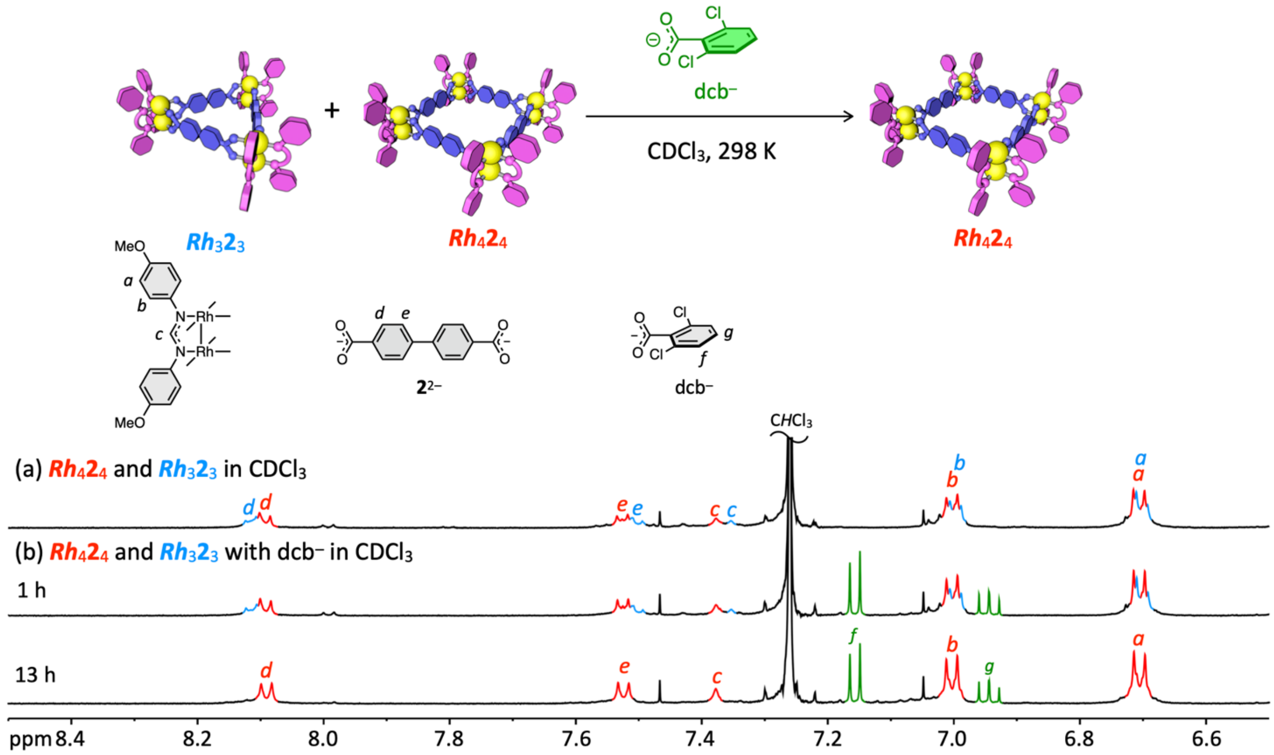

Supplementary Figure 15. The conversion of the ***Rh***$_3$**2**$_3$ triangle into the ***Rh***$_4$**2**$_4$ square by dcb$^-$ in $CDCl_3$. The reaction was monitored by ^{1}H NMR spectroscopy (500 MHz, $CDCl_3$, 298 K, aromatic region). (a) A mixture of the ***Rh***$_3$**2**$_3$ triangle into the ***Rh***$_4$**2**$_4$ square prepared from [***Rh***(CH_3CN)$_4$](BF_4)$_2$ and **2**$^{2-}$ in $CDCl_3$. (b) 2eq. of *n*-Bu_4N·dcb against ***Rh***$^{2+}$ was added in the solution of (a). The ***Rh***$_3$**2**$_3$ triangle was completely converted into the ***Rh***$_4$**2**$_4$ square in 13 h.

Supplementary Table 5. The change in the ratios of ***Rh***$_3$**1**$_3$ and ***Rh***$_4$**1**$_4$ in the conversion process by dcb$^-$.[a]

time (min)	ratio (%)[b] *Rh*$_3$**1**$_3$	*Rh*$_4$**1**$_4$
0	38	62
5	37	63
15	35	65
30	33	67
60	32	68
300	26	74
1080	16	84
2880	4	96

[a]: Experimental condition is shown in Figure 2b.

[b]: determined by ^{1}H NMR spectroscopy based on the internal standard.

Supplementary Table 6. The change in the ratios of ***Rh***$_3$**2**$_3$ and ***Rh***$_4$**2**$_4$ in the conversion process by dcb$^-$.[a]

Time (min)	ratio (%)[b] *Rh*$_3$**2**$_3$	*Rh*$_4$**2**$_4$
0	44	56
5	42	58
15	40	60
30	40	60
60	39	61
780	22	78
2160	6	94

[a]: Experimental condition is shown in Supplementary Figure 15.

[b]: determined by ^{1}H NMR spectroscopy based on the internal standard.

Supramolecular assembly of the *Rh*$_4$1$_4$ square by solvophobic effect

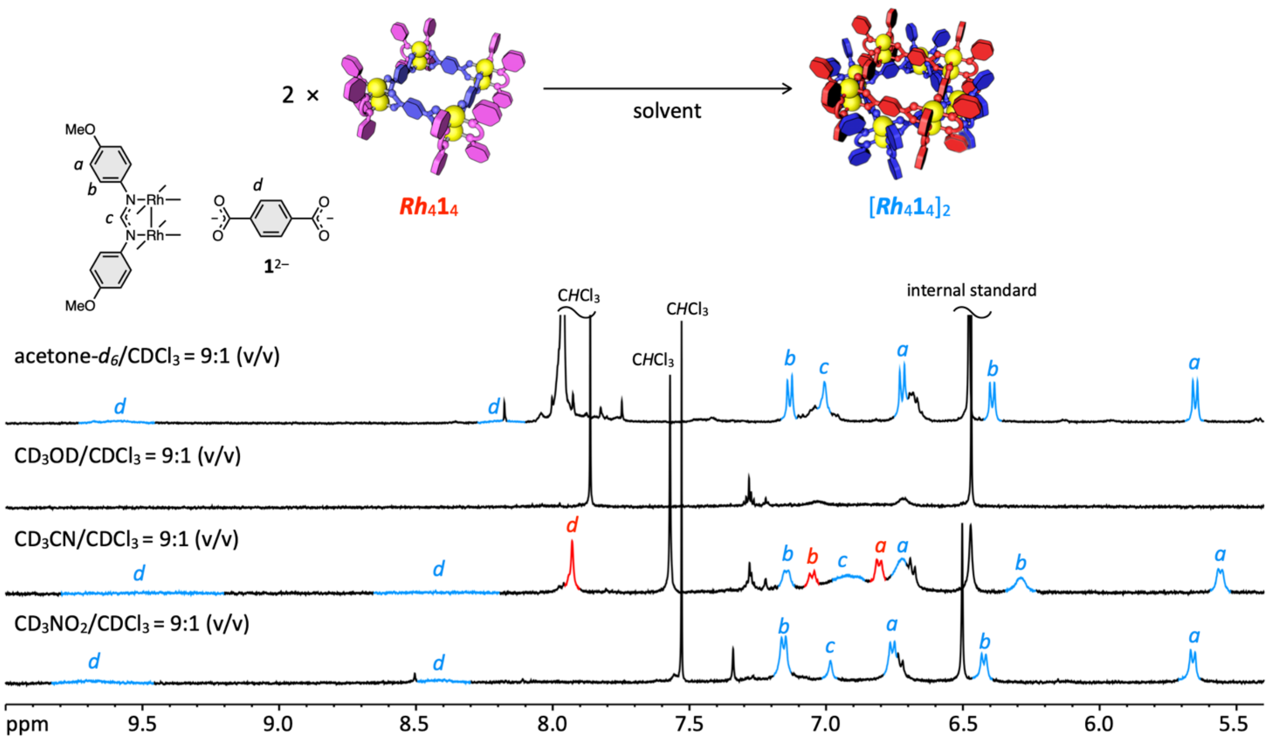

Supplementary Figure 16. ^{1}H NMR spectra (500 MHz, 298 K, aromatic region) of the ***Rh***$_2$**1**$_4$ square measured in various solvents. Because of insolubility of the ***Rh***$_4$**1**$_4$ square, $CDCl_3$ was added in each solvent in 10 volume%. The signals colored in blue and red are assigned to the (***Rh***$_4$**1**$_4$)$_2$ dimer and ***Rh***$_4$**1**$_4$ square, respectively.

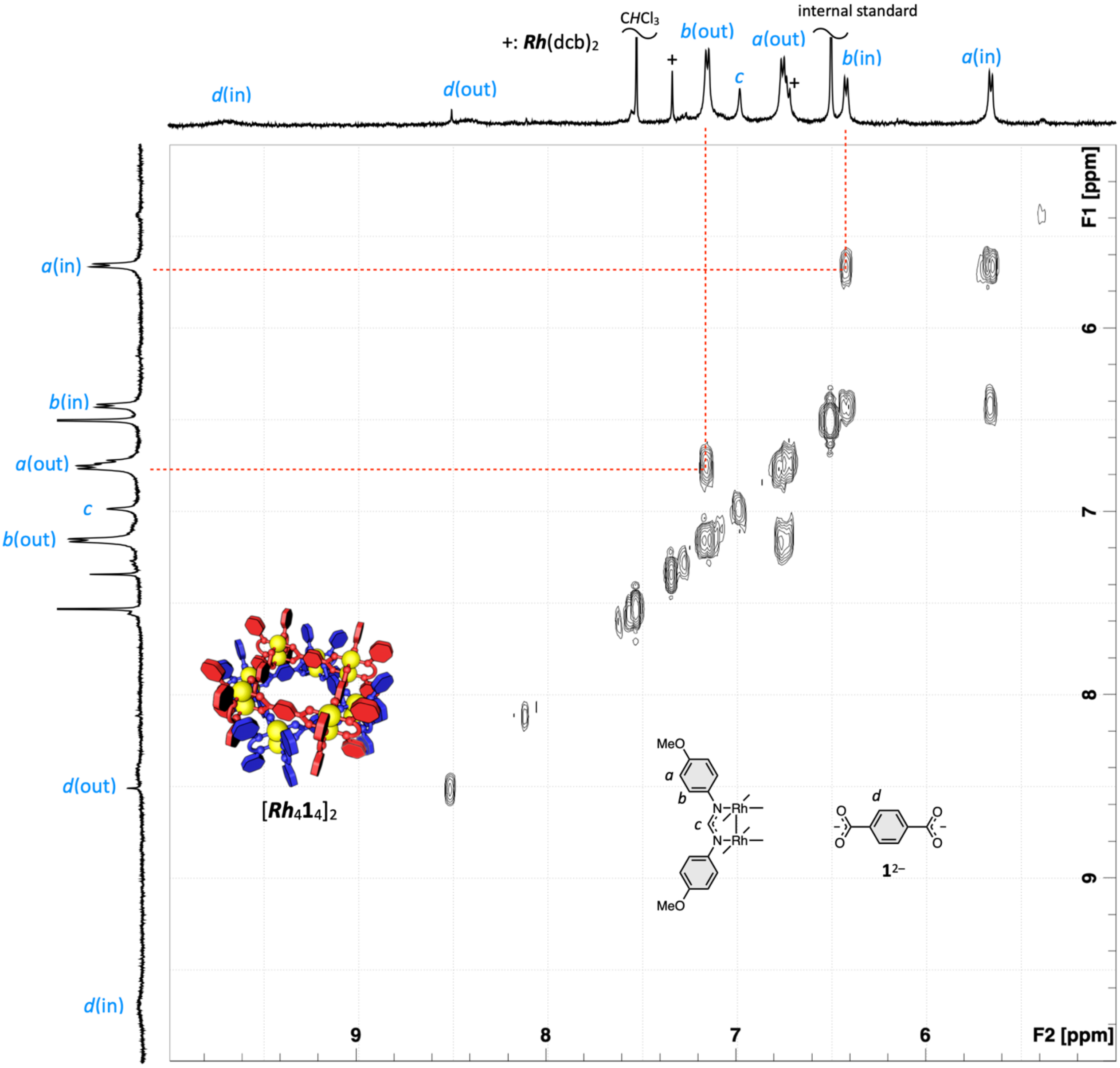

Supplementary Figure 17. (H, H)-COSY spectrum (500 MHz, CD_3NO_2:$CDCl_3$ = 9:1 (v/v), 298 K, aromatic region) of the (***Rh***$_2$**1**$_4$)$_2$ dimer.

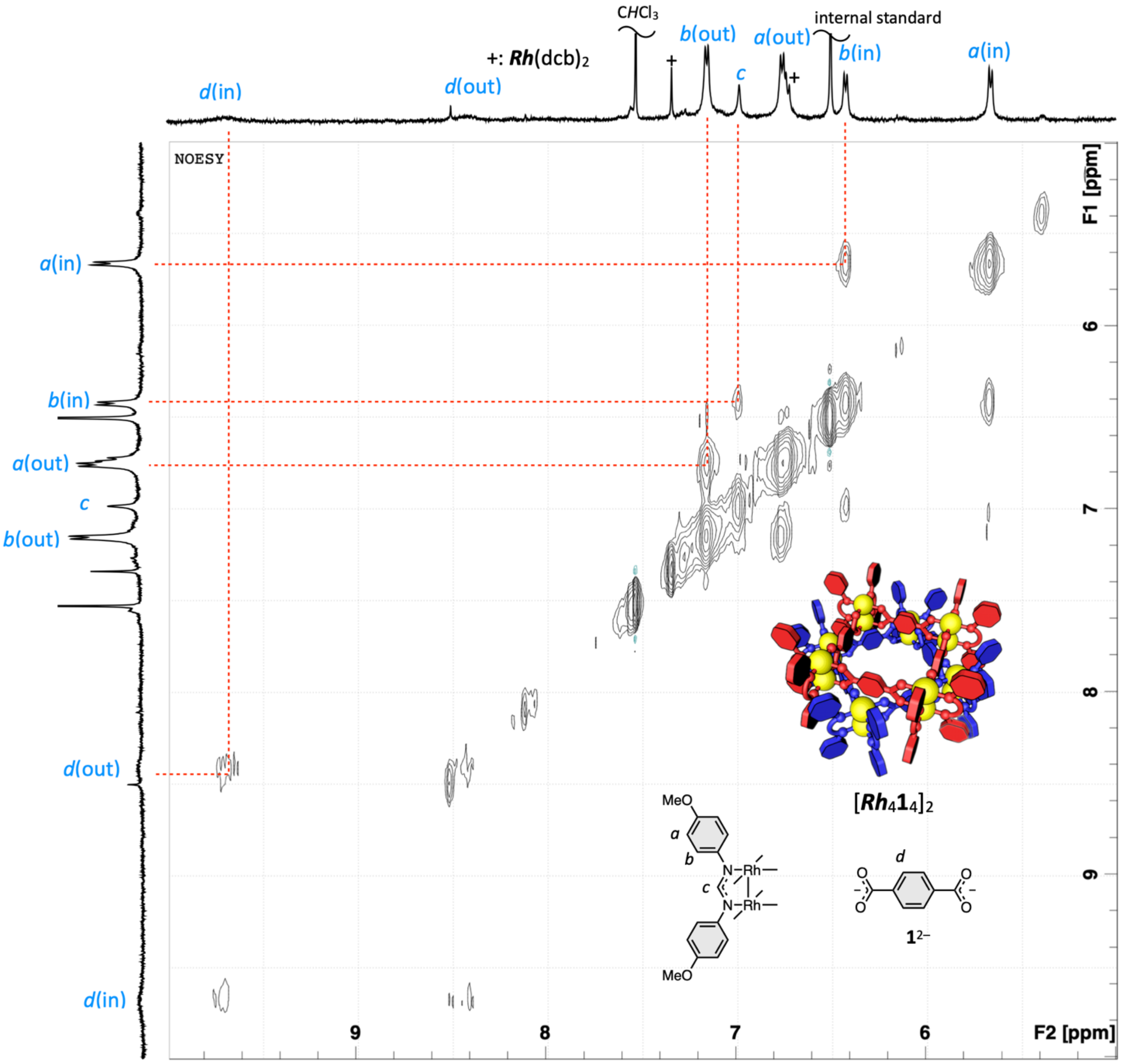

Supplementary Figure 18. (H, H)-NOESY spectrum (500 MHz, CD_3NO_2:$CDCl_3$ = 9:1 (v/v), 298 K, aromatic region) of the (***Rh***$_2$**1**$_4$)$_2$ dimer.

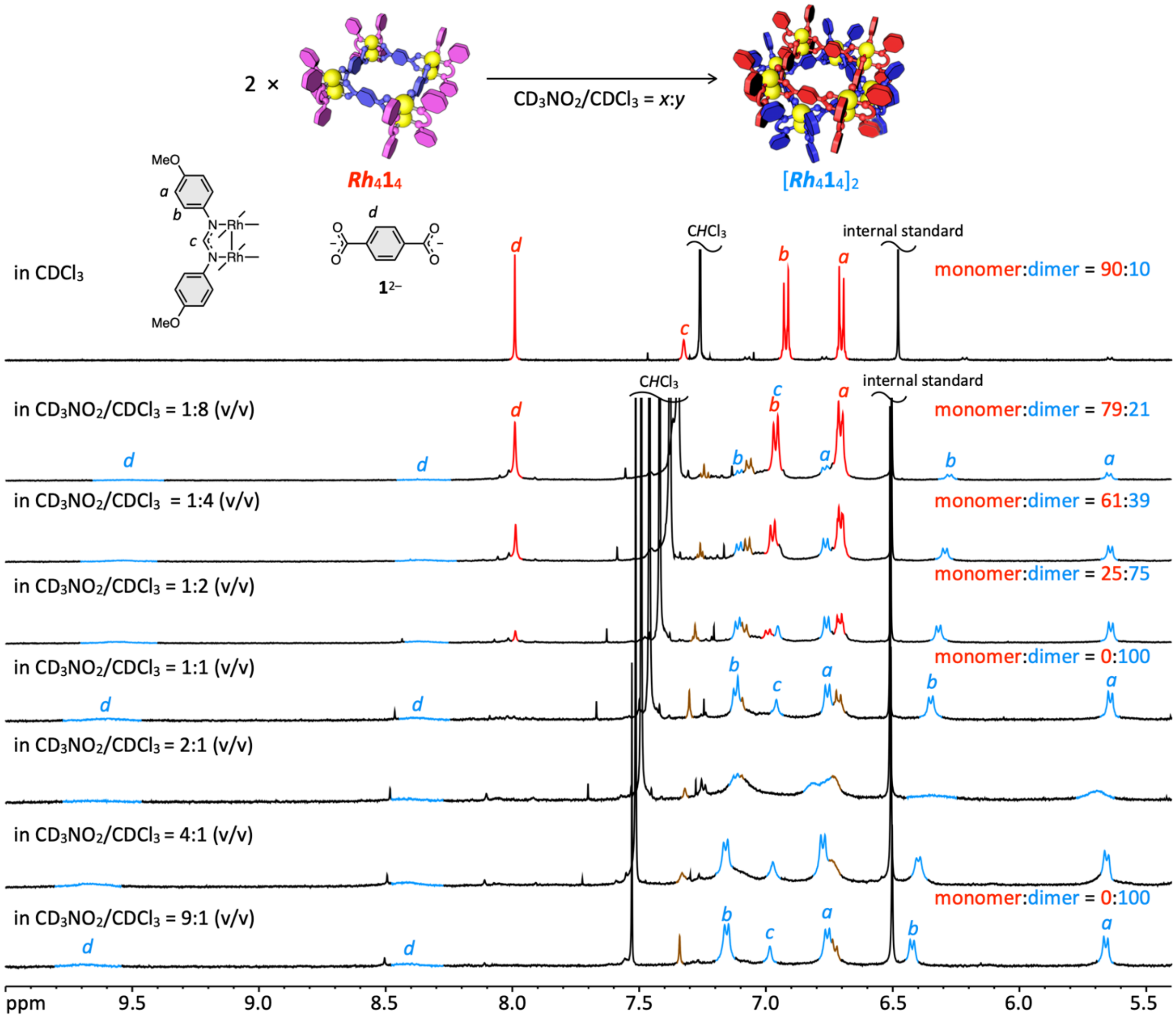

Supplementary Figure 19. ^{1}H NMR spectra (500 MHz, 298 K, aromatic region) of the $\boldsymbol{Rh_2 1_4}$ square in different composition ratios of $CDCl_3$ and CD_3NO_2. The signals colored in blue and red are assigned to the ($\boldsymbol{Rh_4 1_4}$)$_2$ dimer and $\boldsymbol{Rh_4 1_4}$ square, respectively. With increase in the $CD_3NO_2/CDCl_3$ ratio, the signals of the ($\boldsymbol{Rh_4 1_4}$)$_2$ dimer (colored in blue) increased, indicating that the dimer formation in solution is due to the solvophobic effect of CD_3NO_2.

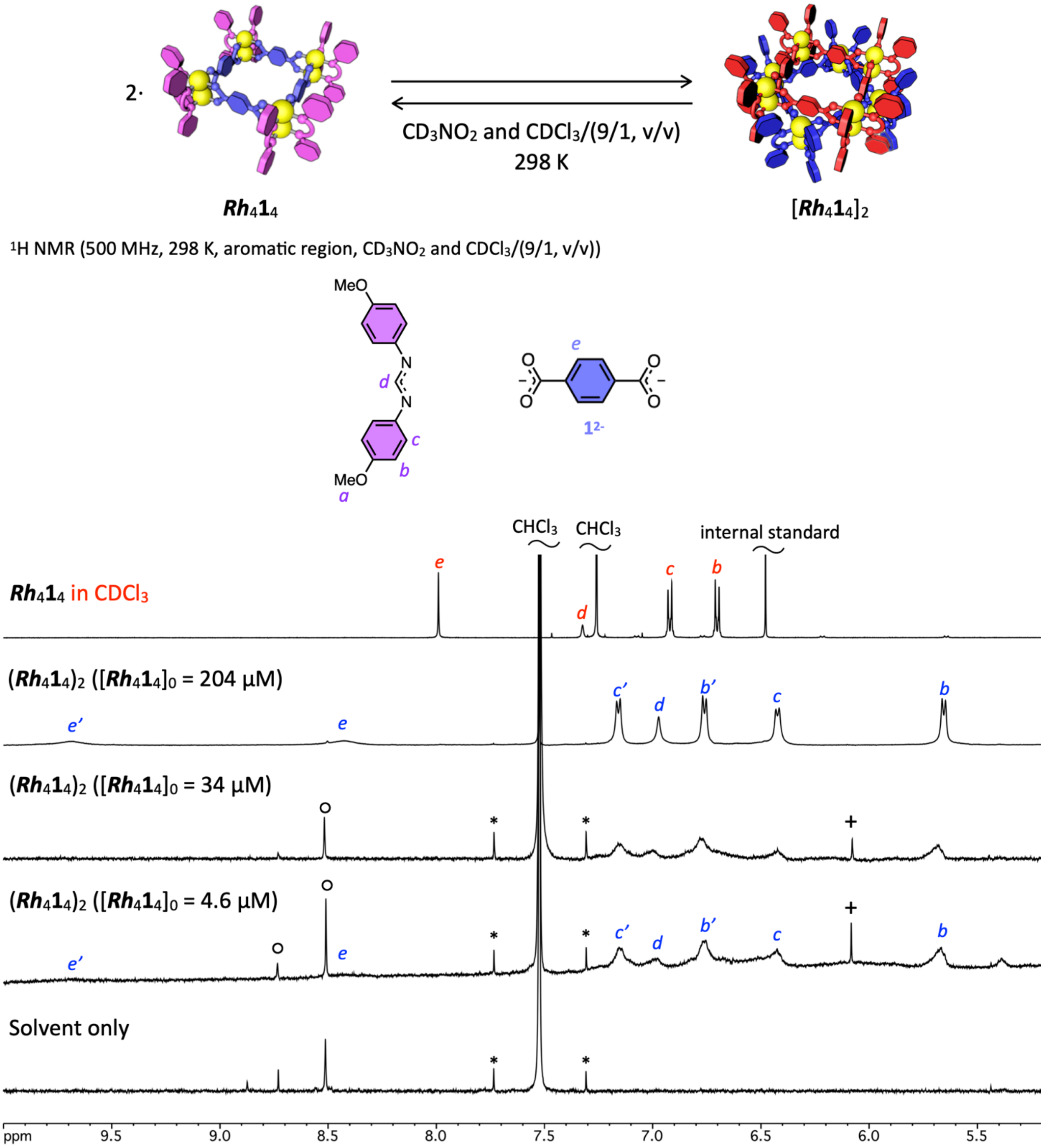

Supplementary Figure 20. ^{1}H NMR spectra (500 MHz, 298 K, aromatic region, CD_3NO_2 and $CDCl_3$ (9:1 (v/v)) of the (***Rh***$_4$**1**$_4$)$_2$ dimer at different concentrations. When the concentration of the ***Rh***$_4$**1**$_4$ square is 4.6 μM, the signals of the monomer were not found. Under the assumption that 10% of monomer exists in the solution of [***Rh***$_4$**1**$_4$]$_0$ = 4.6 μM, the dimerization constant K for the equilibrium (2·***Rh***$_4$**1**$_4$ ⇄ (***Rh***$_4$**1**$_4$)$_2$) is estimated to be K = [(***Rh***$_4$**1**$_4$)$_2$]/([***Rh***$_4$**1**$_4$]^2) = 9.8 × 10^6 M^{-1}. The asterisks indicate the carbon satellite of $CHCl_3$. Open circles indicate species derived from the solvent. The signal indicated by "+" could not be identified.

X-ray crystallographic structural analysis of the [Rh_4**1**$_4$(dmso-*S*)$_4$]$_2$ dimer

Supplementary Table 7. X-ray crystallographic data for [$\boldsymbol{Rh_4}$**1**$_4$(dmso-*S*)$_4$]$_2$.

Compound	[$Rh_4$1$_4$(dmso-S)$_4$]$_2$
Formula	$C_{160}H_{148}N_{16}O_{36}Rh_8S_4$
Formula weight	3822.46
Habit	green, plate
Crystal size /mm^3	0.15 × 0.15 × 0.03
T/K	93
Crystal system	monoclinic
Space group	$C2/c$
a/ Å	44.9500(10)
b/ Å	26.3640(5)
c/ Å	37.4107(9)
α/º	90
β/º	99.818(2)
γ/º	90
V/ Å^3	43684.7(17)
Z	8
d_{calc}/g cm^{-3}	1.162
F(000)	15456
μ(Mo $K\alpha$)/mm^{-1}	0.686
GOF	1.037
No. of reflns	162809
Unique data	44330
R_{int}	0.0668
$R_1{}^a$ ($F^2 > 2\sigma(F^2)$)	0.0697
$wR_2{}^b$ (all data)	0.1781

^{a} $R_1 = \Sigma||F_o| - ||F_c||/\Sigma|F_o|$. ^{b} $wR_2 = [\Sigma w(F_o{}^2 - F_c{}^2)^2/\Sigma w(F_o{}^2)^2]^{1/2}$.
