# Supplementary material for: Pathway selection in the self-assembly of Rh4L4 coordination squares under kinetic control
Source: Commun Chem. 2023 Nov 15;6:248. doi: 10.1038/s42004-023-01053-7 (PMC10651846; doi:10.1038/s42004-023-01053-7)
Supplement: Supplementary file 3 — Description of additional supplementary files [file 42004_2023_1053_MOESM3_ESM.pdf]

Description of Additional Supplementary Files

File Name: Supplementary Data 1

Description: NMR spectra of substrates

File Name: Supplementary Data 2

Description: Crystallographic information file of [$\boldsymbol{Rh}_4\mathbf{1}_4$(dmso-$S$)$_4$]$_2$
