# Supplementary material for: Pathway selection in the self-assembly of Rh4L4 coordination squares under kinetic control
Source: Commun Chem. 2023 Nov 15;6:248. doi: 10.1038/s42004-023-01053-7 (PMC10651846; doi:10.1038/s42004-023-01053-7)

^{1}H NMR spectrum of [***Rh***(CH_3CN)$_6$](BF_4)$_2$ (500 MHz, CD_3CN)

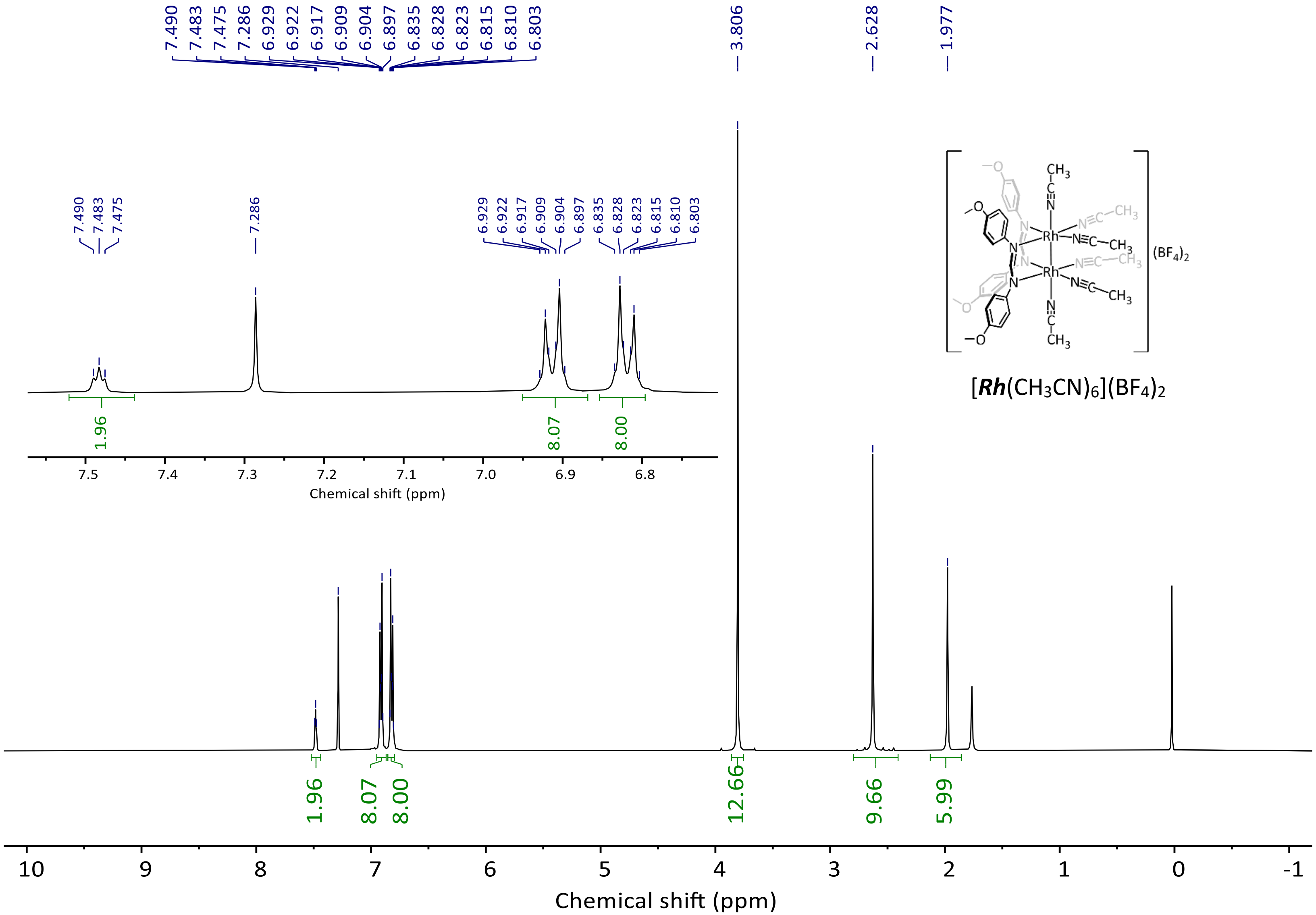

^{13}C{^{1}H} NMR spectrum of [***Rh***($CH_3CN)_6](BF_4)_2$ (126 MHz, $CDCl_3$)

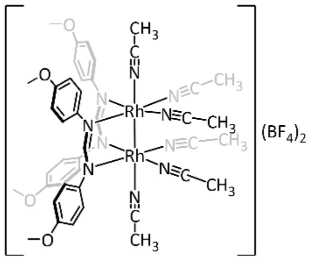

[***Rh***($CH_3CN)_6](BF_4)_2$

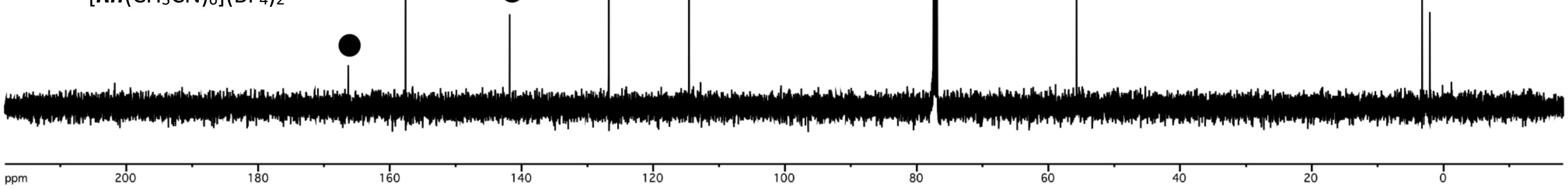

^{1}H NMR spectrum of HDAniF (500 MHz, CD_3Cl)

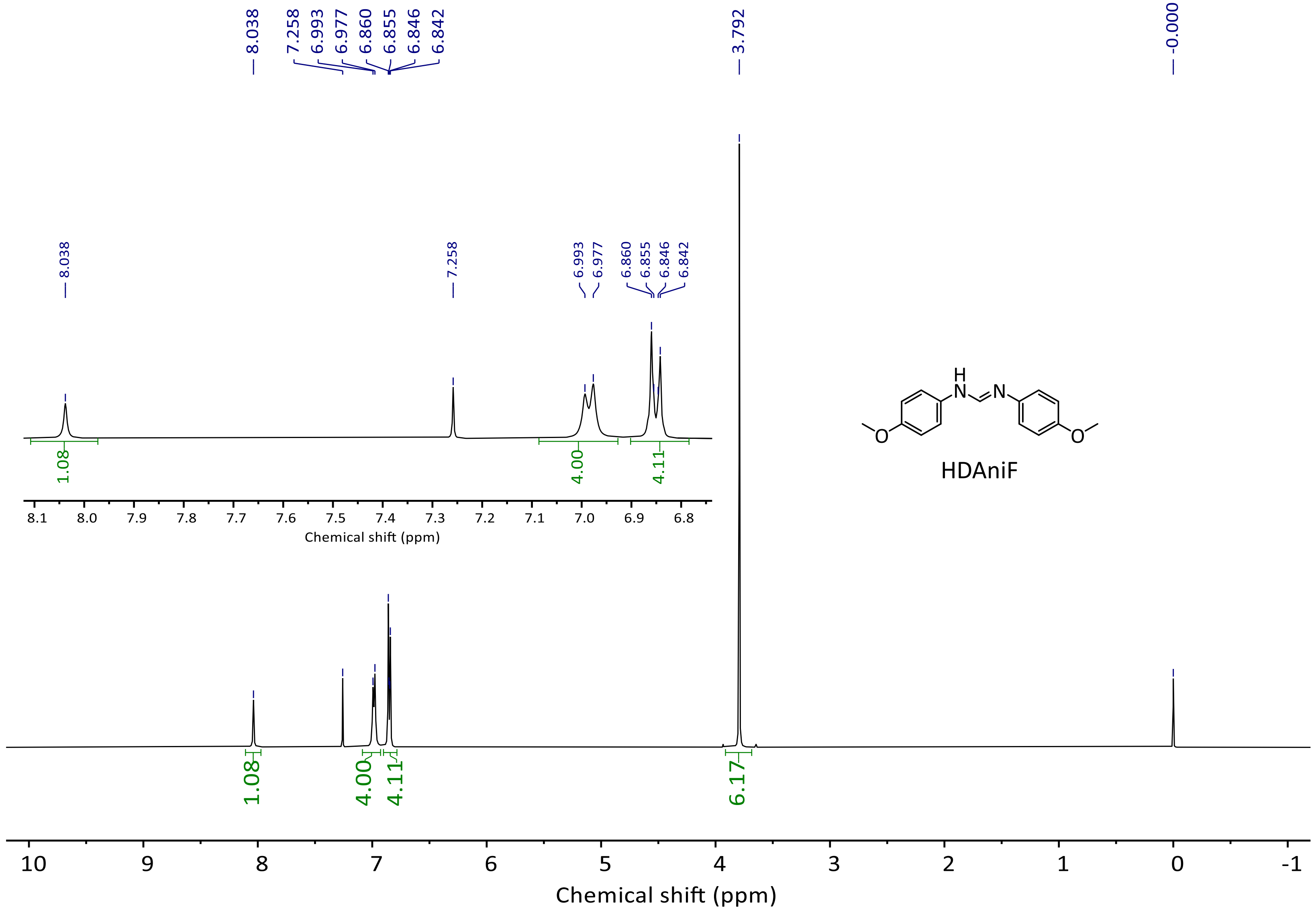

$^{13}C\{^{1}H\}$ NMR spectrum of HDAniF (126 MHz, $CDCl_3$)

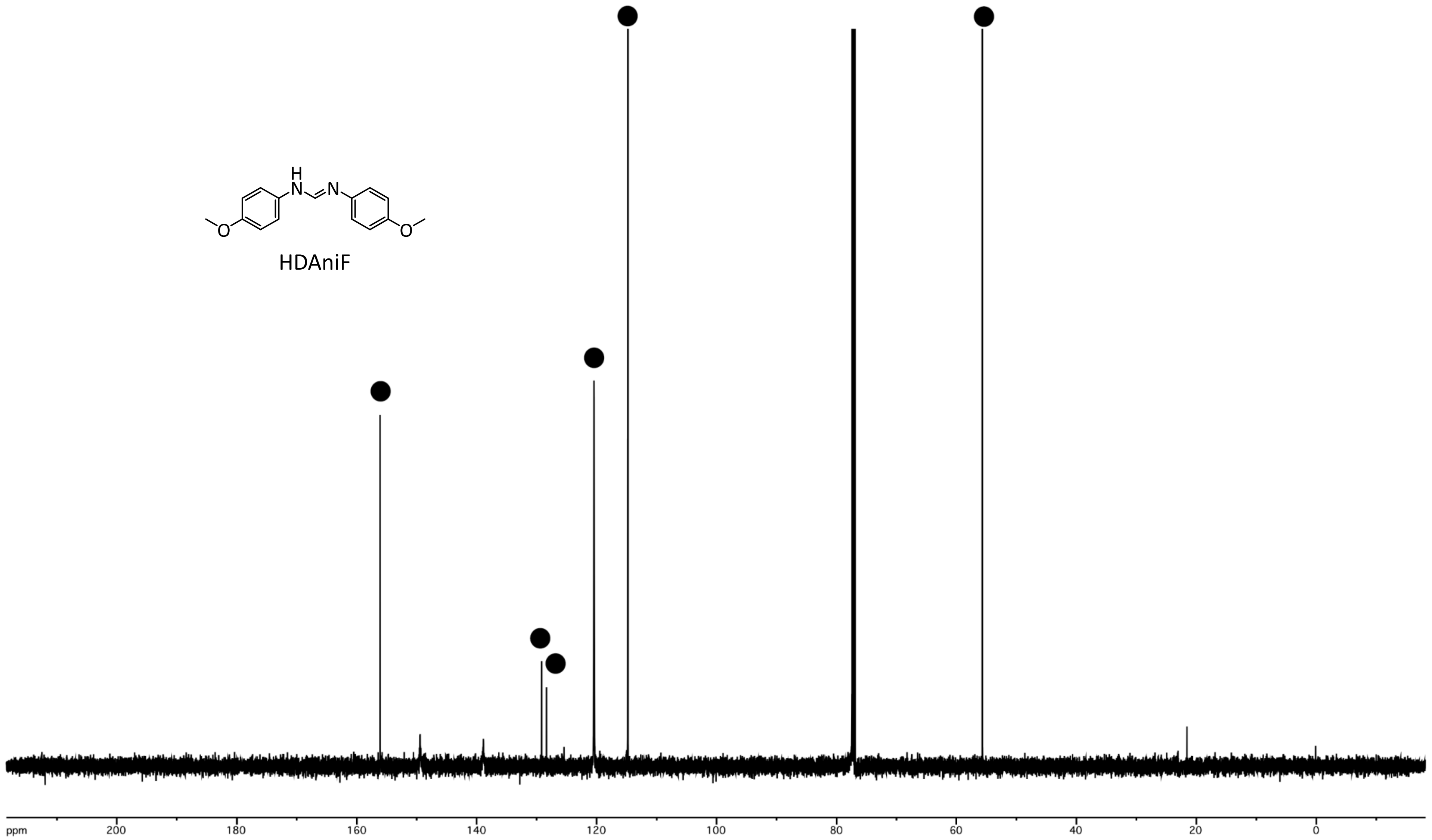

^{1}H NMR spectrum of ***Rh***(dcb)$_2$ (500 MHz, CD_3Cl)

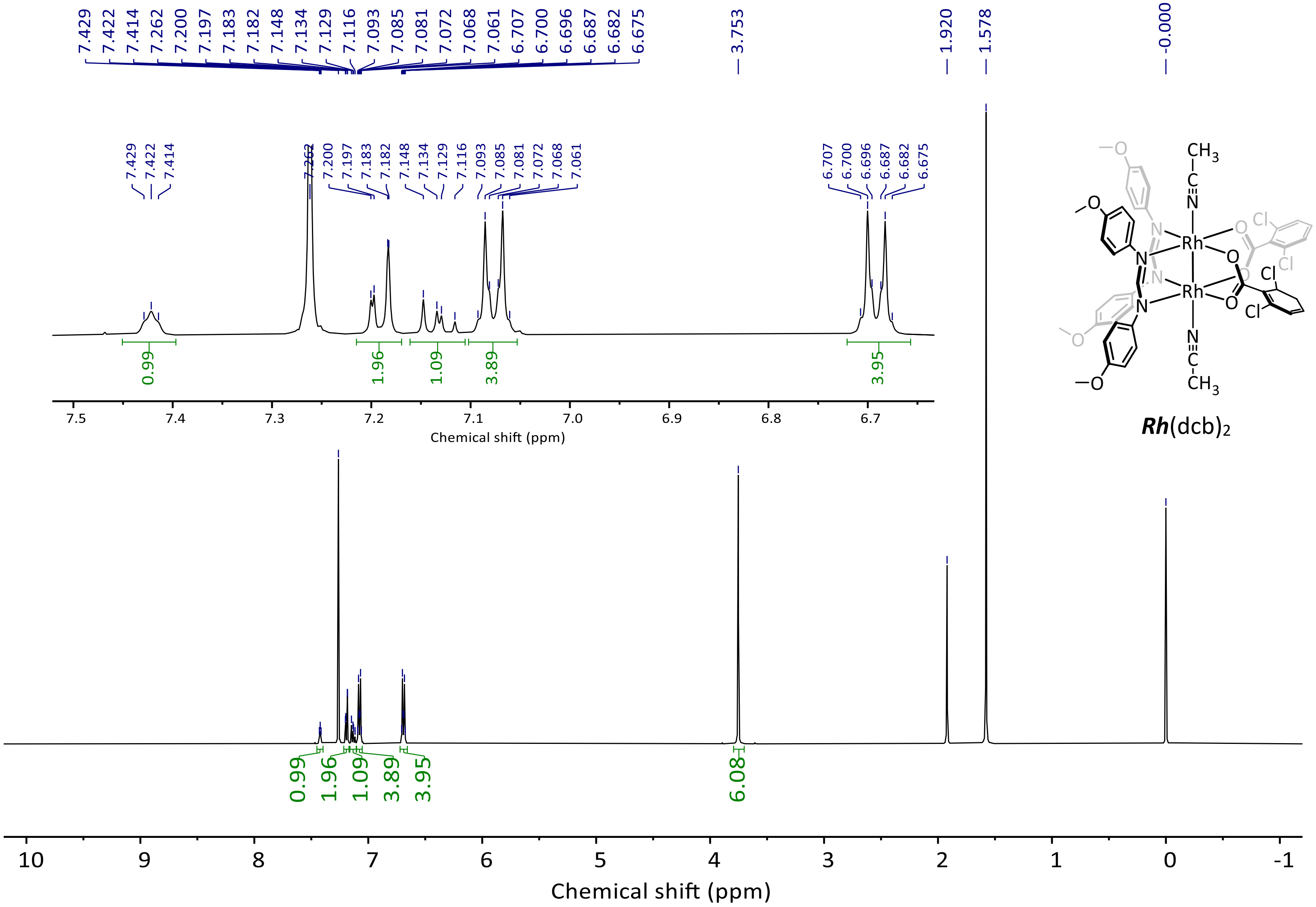

Rh(dcb)$_2$

$^{13}C\{^{1}H\}$ NMR spectrum of ***Rh***(dcb)$_2$ (126 MHz, CD_3Cl)

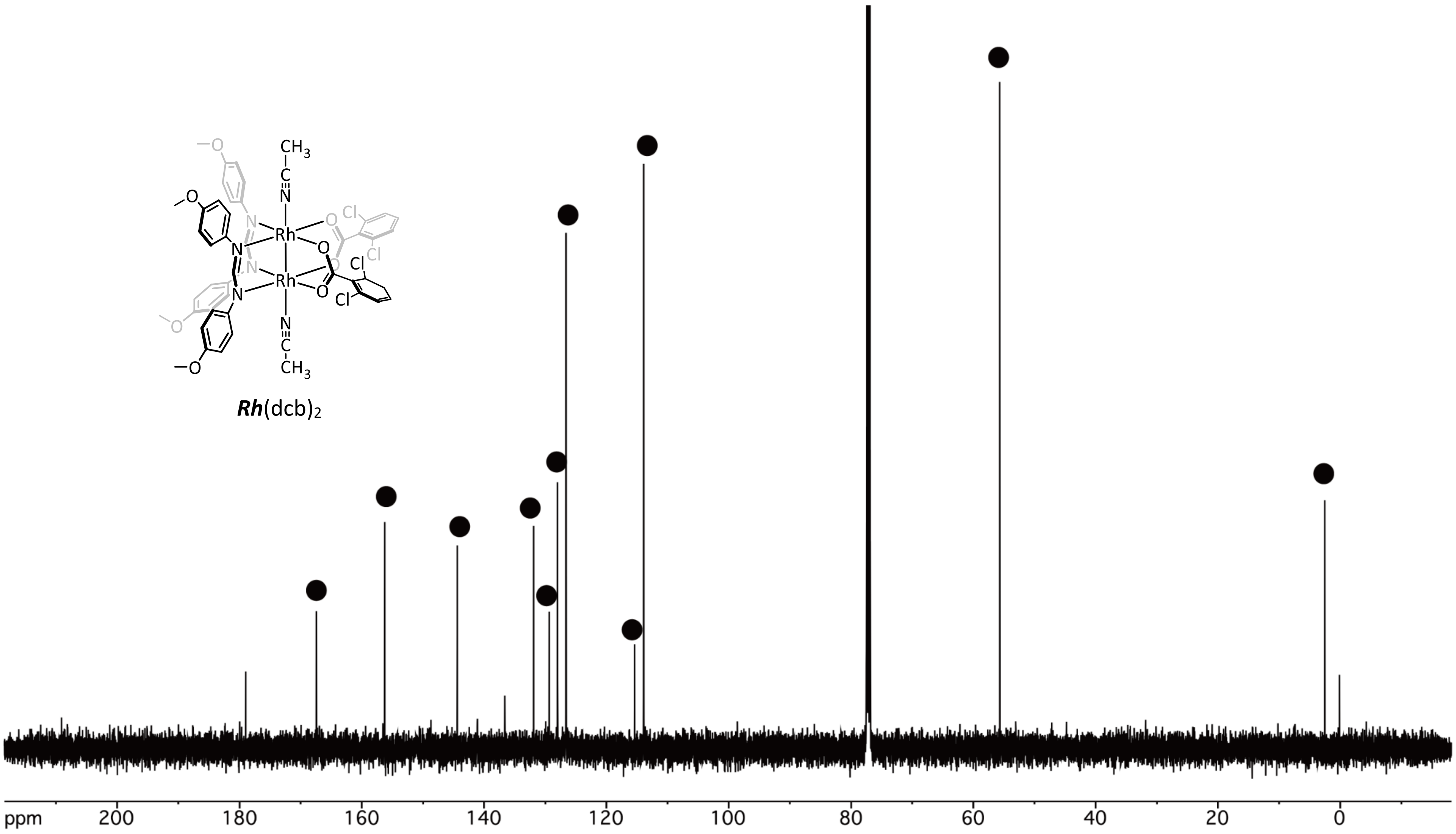

^{1}H NMR spectrum of *n*-Bu_4N·dcb (500 MHz, DMSO-d_6)

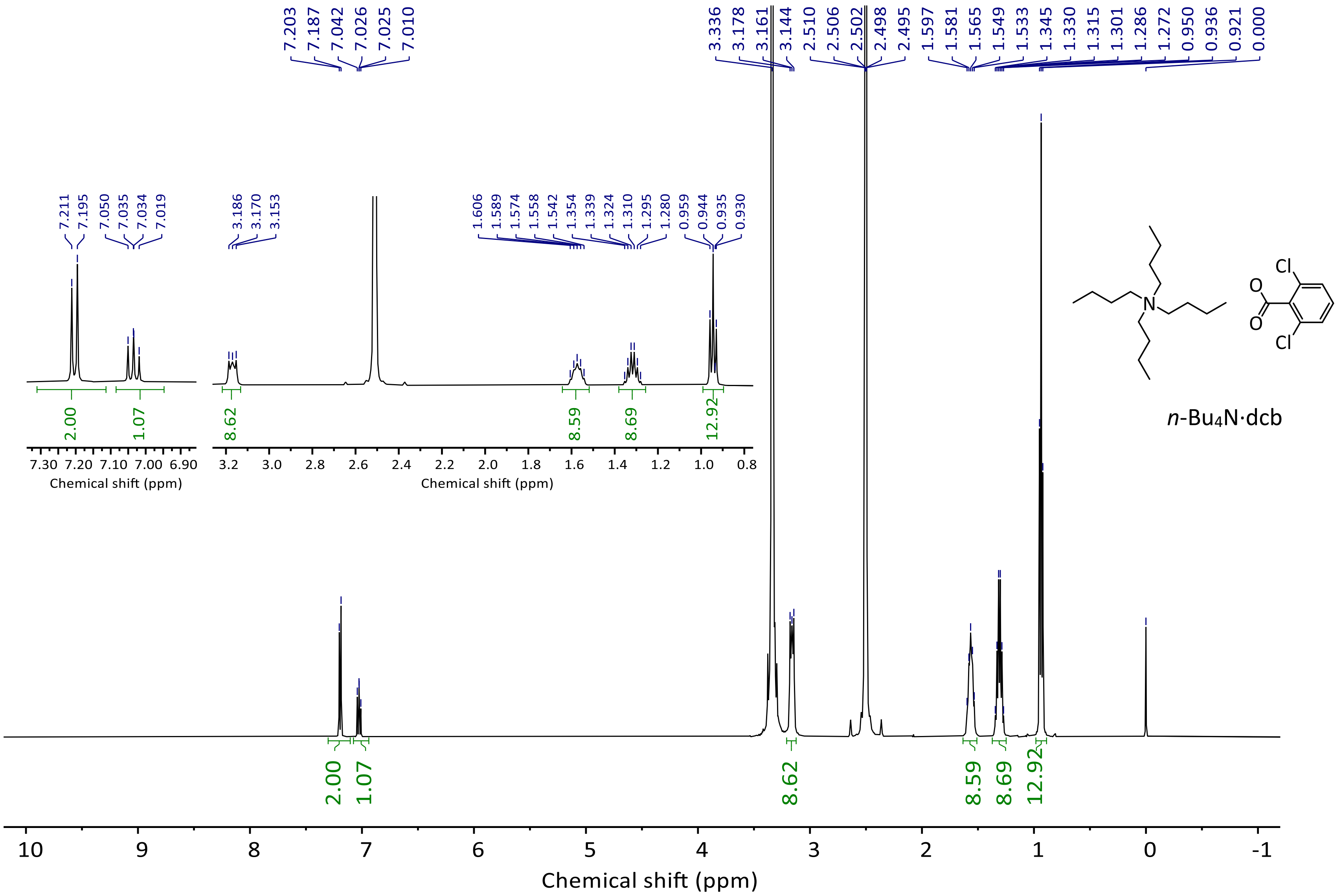

$^{13}C\{^{1}H\}$ NMR spectrum of *n*-Bu_4N·dcb (126 MHz, $CDCl_3$)

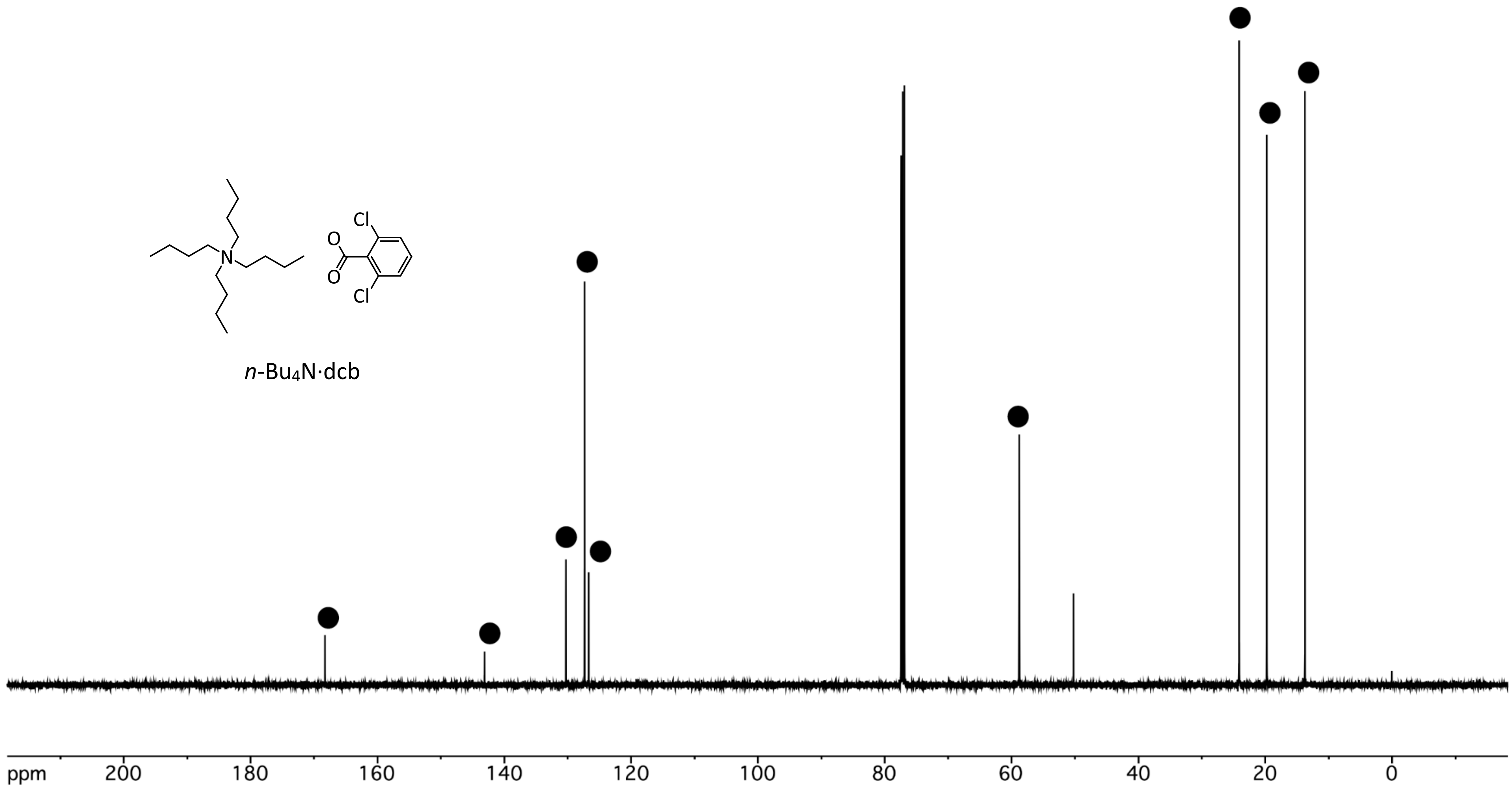

^{1}H NMR spectrum of $(n\text{-}Bu_4N)_2\cdot\mathbf{1}$ (500 MHz, $CDCl_3$)

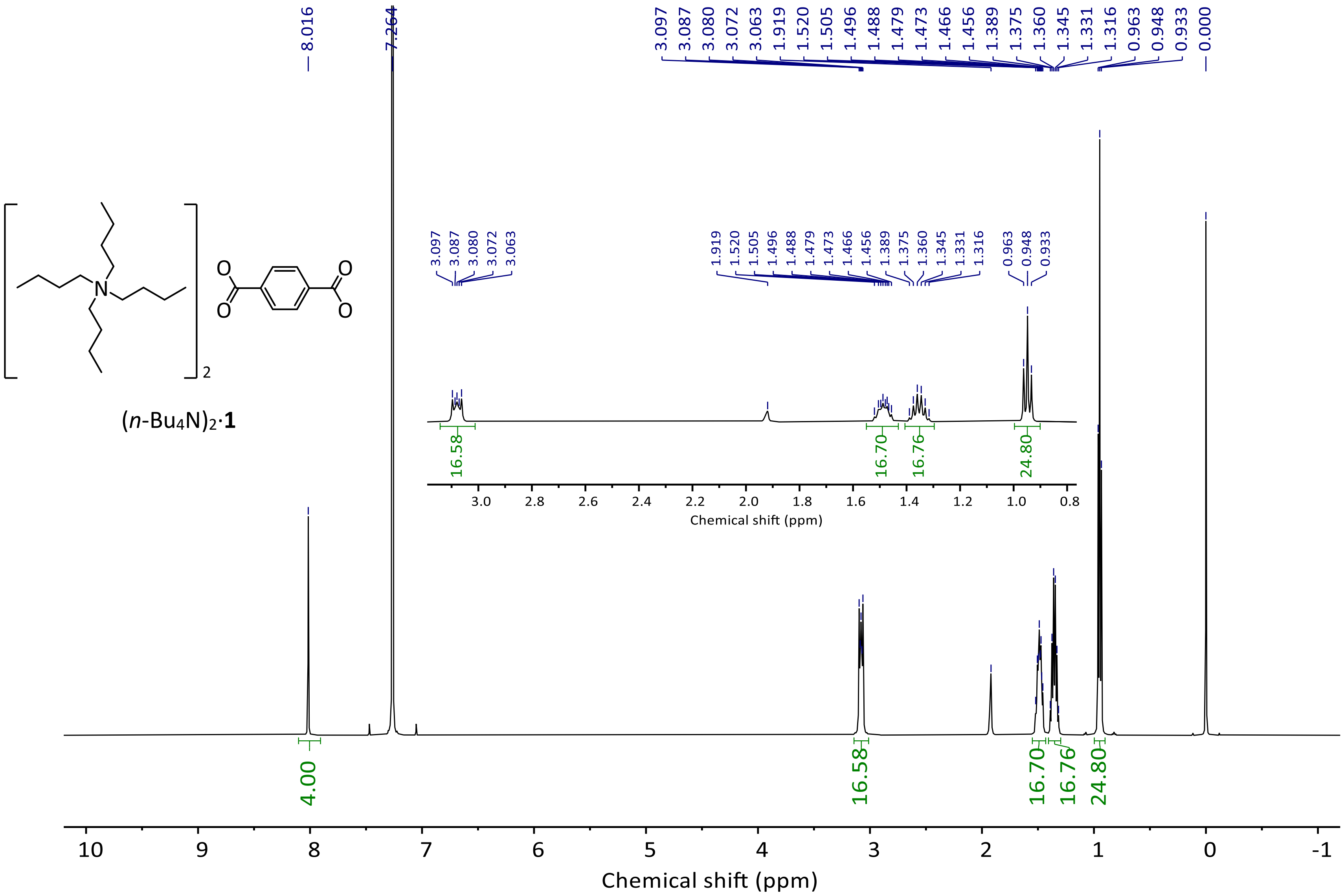

$^{13}C\{^1H\}$ NMR spectrum of (n-$Bu_4N)_2$·**1** (126 MHz, $CDCl_3$)

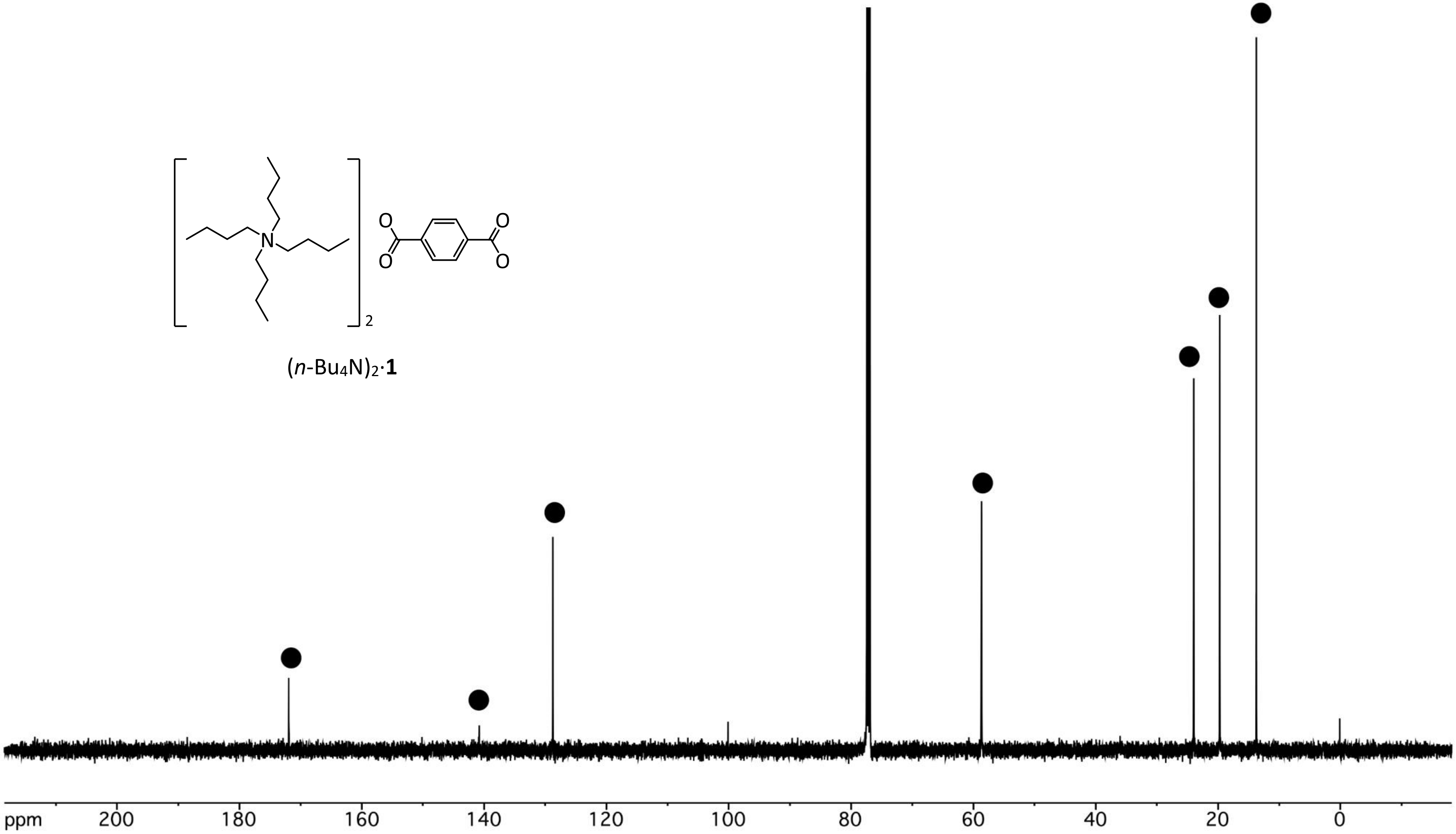

^{1}H NMR spectrum of $(n\text{-}Bu_4N)_2\cdot$**2** (500 MHz, $CDCl_3$)

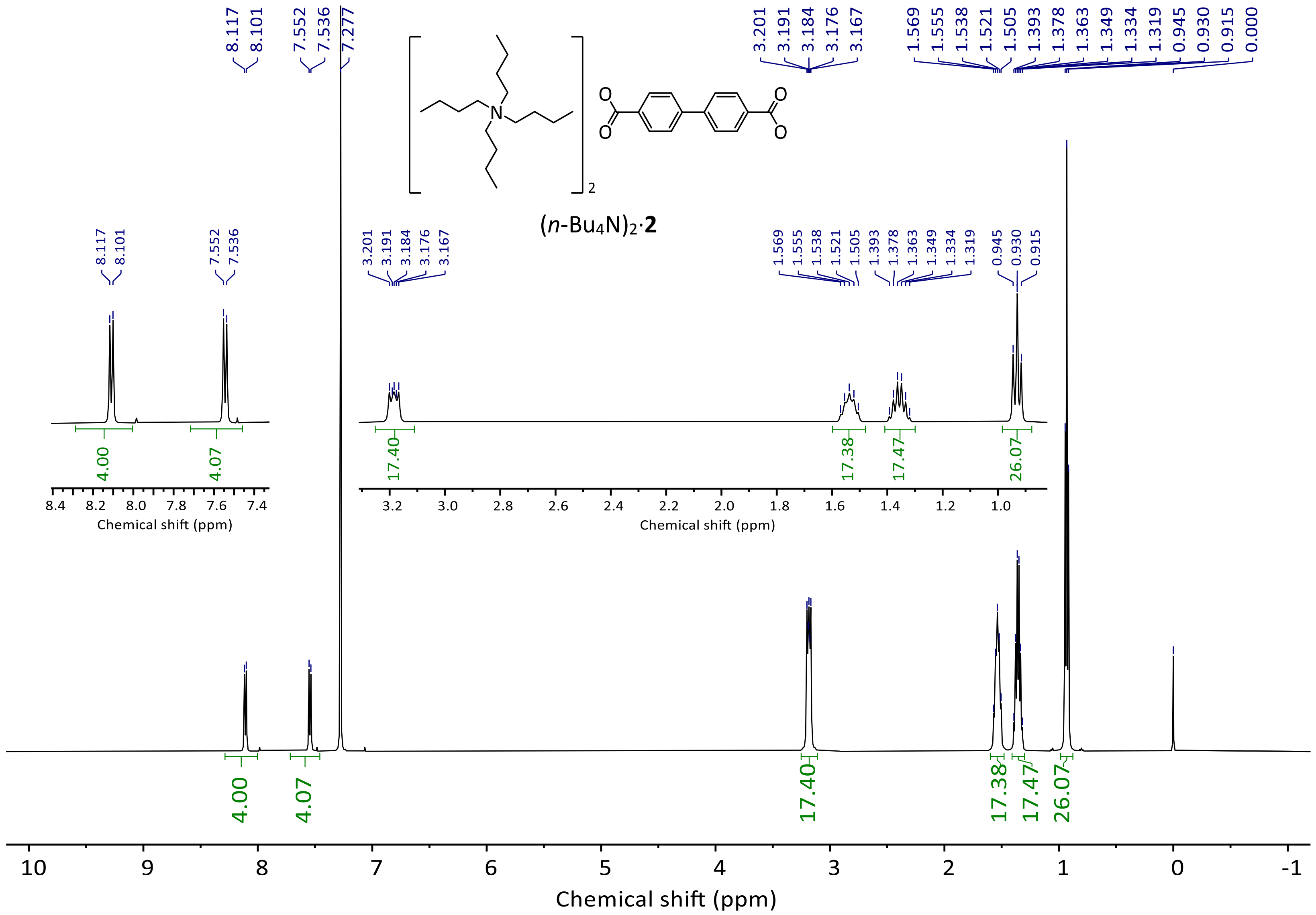

$^{13}C\{^1H\}$ NMR spectrum of $(n\text{-}Bu_4N)_2\cdot\mathbf{2}$ (126 MHz, $CDCl_3$)

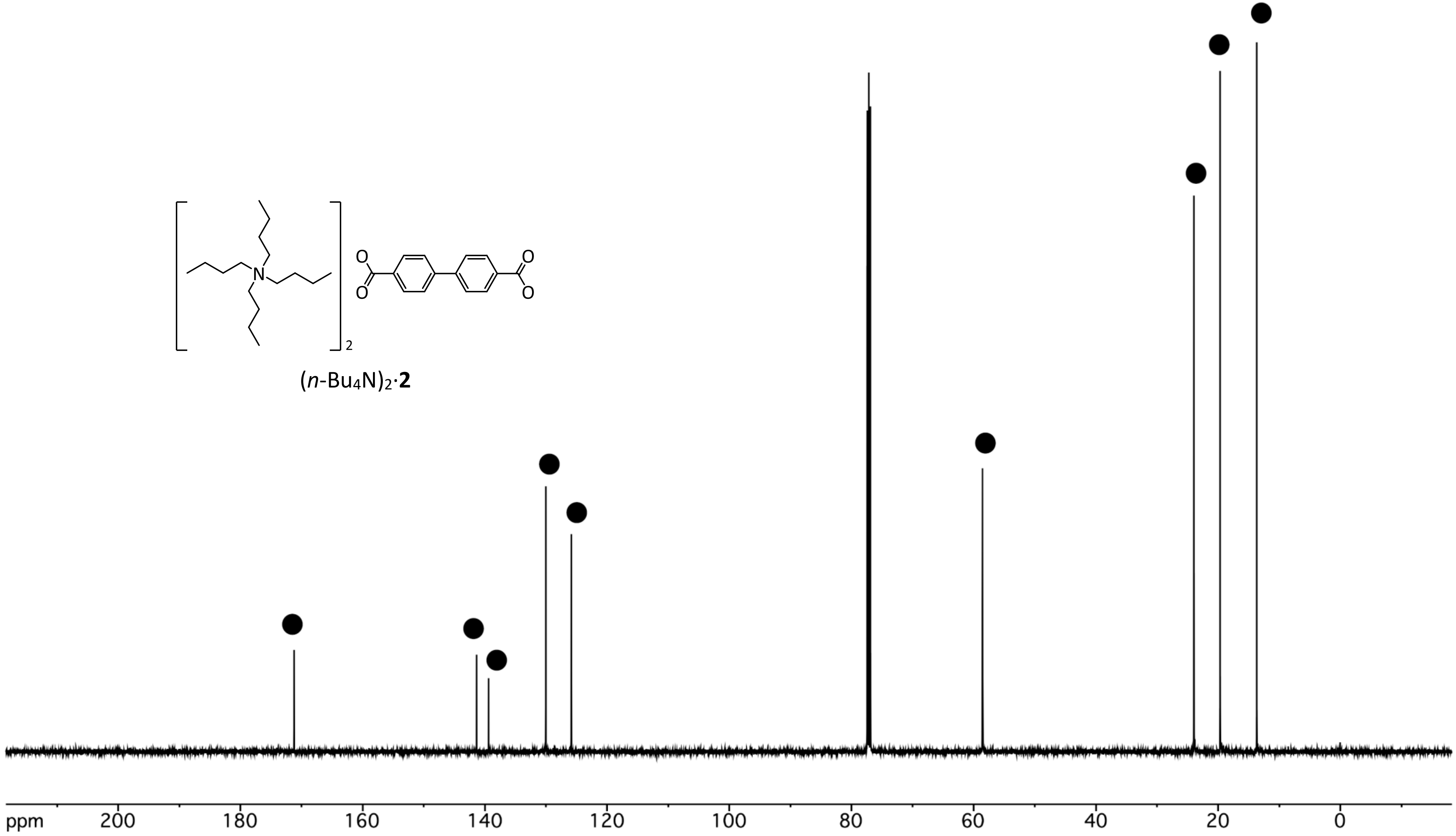

^{1}H NMR spectrum of *n*-Bu_4N·tol (500 MHz, $CDCl_3$)

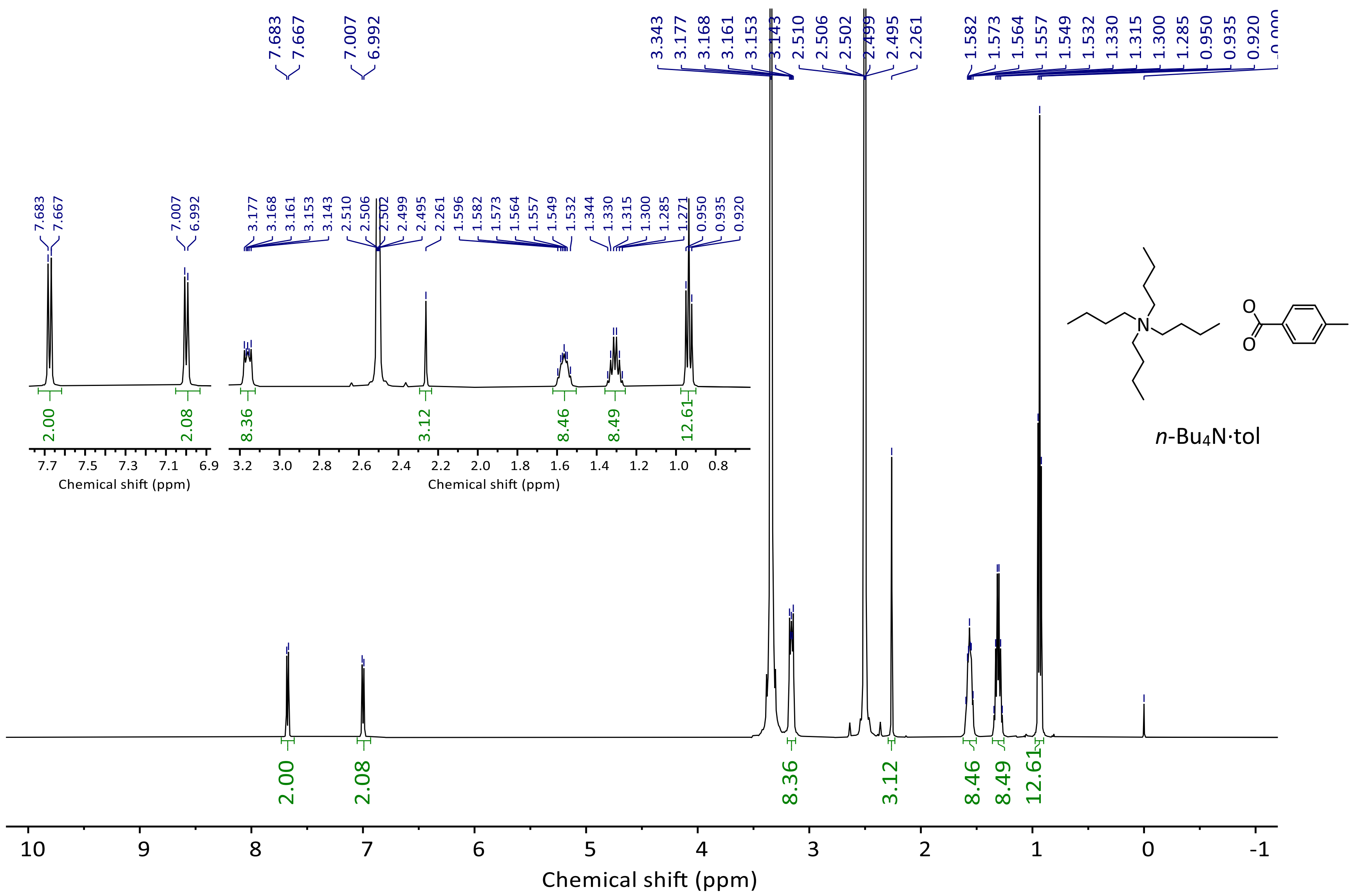

$^{13}C\{^1H\}$ NMR spectrum of *n*-Bu_4N·tol (126 MHz, $CDCl_3$)

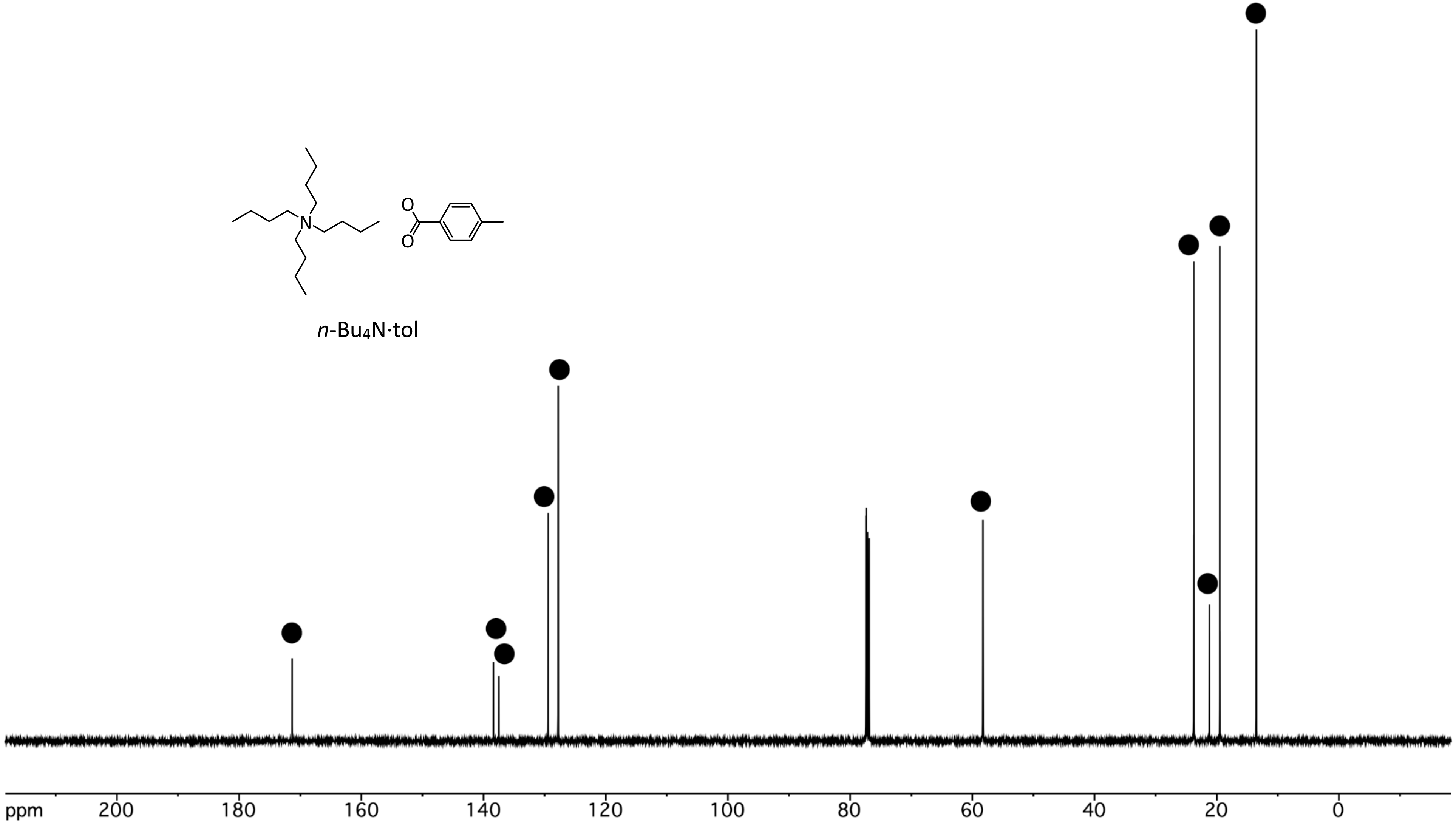

Supplement: Supplementary file 4 — Supplementary data 1 [file 42004_2023_1053_MOESM4_ESM.pdf]
